# Supplementary material for: Selective cargo and membrane recognition by SNX17 regulates its interaction with Retriever
Source: EMBO Rep. 2024 Dec 9;26(2):470–93. doi: 10.1038/s44319-024-00340-1 (PMC11772769; doi:10.1038/s44319-024-00340-1)
Supplement: Supplementary file 1 — Appendix [file 44319_2024_340_MOESM1_ESM.pdf]

Selective cargo and membrane recognition by SNX17 regulates its interaction with Retriever

Aurora Martín-González, Iván Méndez-Guzmán, Maialen Zabala-Zearreta, Andrea Quintanilla, Arturo García-López, Eva Martínez-Lombardía, Juan Carlos Acosta, David Albesa-Jové, María Lucas

Appendix

Table of contents:

Appendix Figure S1 ____________________________ 2

Appendix Figure S2 ____________________________ 2

Appendix Figure S3 ____________________________ 3

Appendix Figure S4 ____________________________ 4

Appendix Figure S5 ____________________________ 6

Appendix Figure S6 ____________________________ 7

Appendix Figure S7 ____________________________ 8-9

Appendix Figure S8 ____________________________ 9

Appendix Figure S9 ____________________________ 9

Appendix Figure S10 ___________________________ 10

Appendix Figure S11 ___________________________ 11

Appendix Figure S12 ___________________________ 12

Appendix Table S1 ____________________________ 13

Appendix Table S2 ____________________________ 14

Appendix Table S3 ____________________________ 16

Appendix Table S4 ____________________________ 20

Appendix Table S5 ____________________________ 27

Appendix Table S6 ____________________________ 28

References ___________________________________ 29

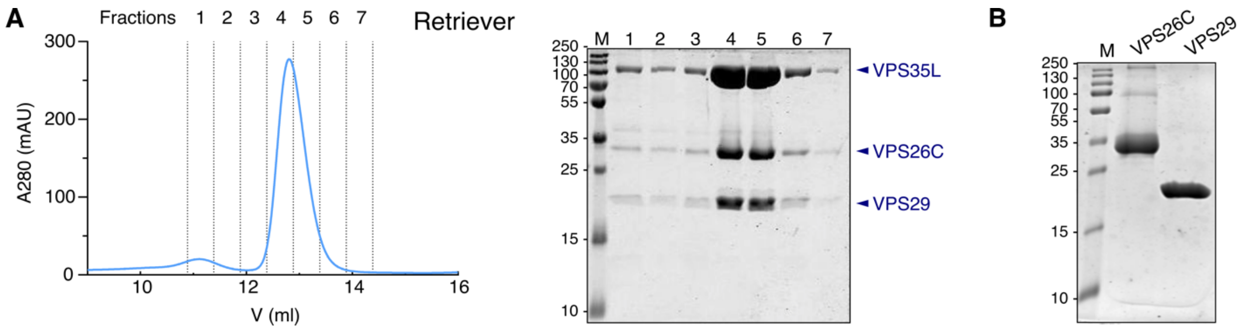

Appendix Figure S1. Purification of the Retriever complex and its individual subunits.

(**A**) Coomassie-stained SDS-PAGE analysis of the purified Retriever complex composed of VPS35L, VPS26C, and VPS29 (right) from the fractions obtained in a Superdex 200 Increase 10/300 gel filtration chromatography (left). Two peaks containing the three proteins are observed, which correspond to the dimeric (first peak) and monomeric (second peak) forms of the complex. M, protein marker.

(**B**) Coomassie-stained SDS-PAGE gel of purified VPS26C and VPS29 subunits of the Retriever complex.

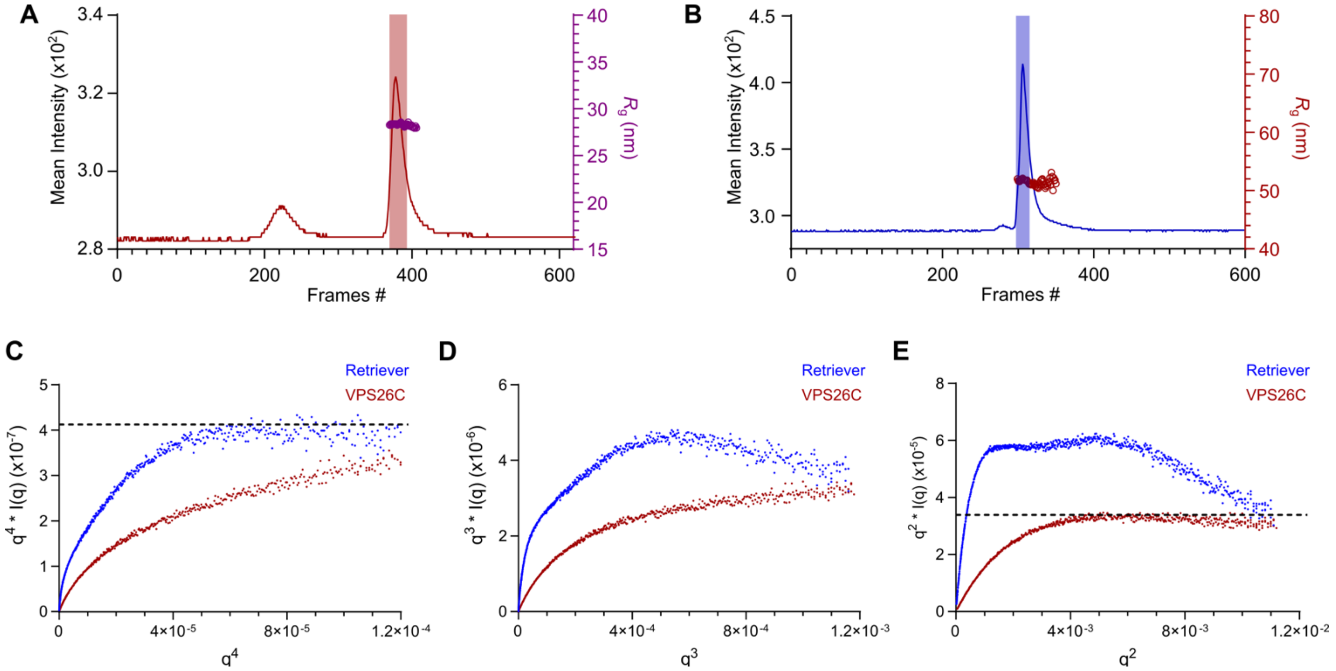

Appendix Figure S2. SAXS analysis of VPS26C and Retriever.

(**A-B**) SEC-SAXS elution profiles of VPS26C (**A**) and the Retriever complex (**B**). Frames are plotted as a function of the mean intensity. The selected range of frames to average the data, marked with a shaded area, exhibited a flat distribution of Rg values, indicating that the sample was monodisperse.

(**C-E**) Porod-Debye plots illustrating the highly flexible nature of VPS26C. Comparison of the changes in the Porod-Debye regions between $q^4 I(q) vs. q^4$ (**C**), $q^3 I(q) vs. q^3$ (**D**), $q^2 I(q) vs. q^2$ (**E**) plots for both VPS26C and the Retriever complex. A discontinuous line has been drawn to indicate the asymptotic region.

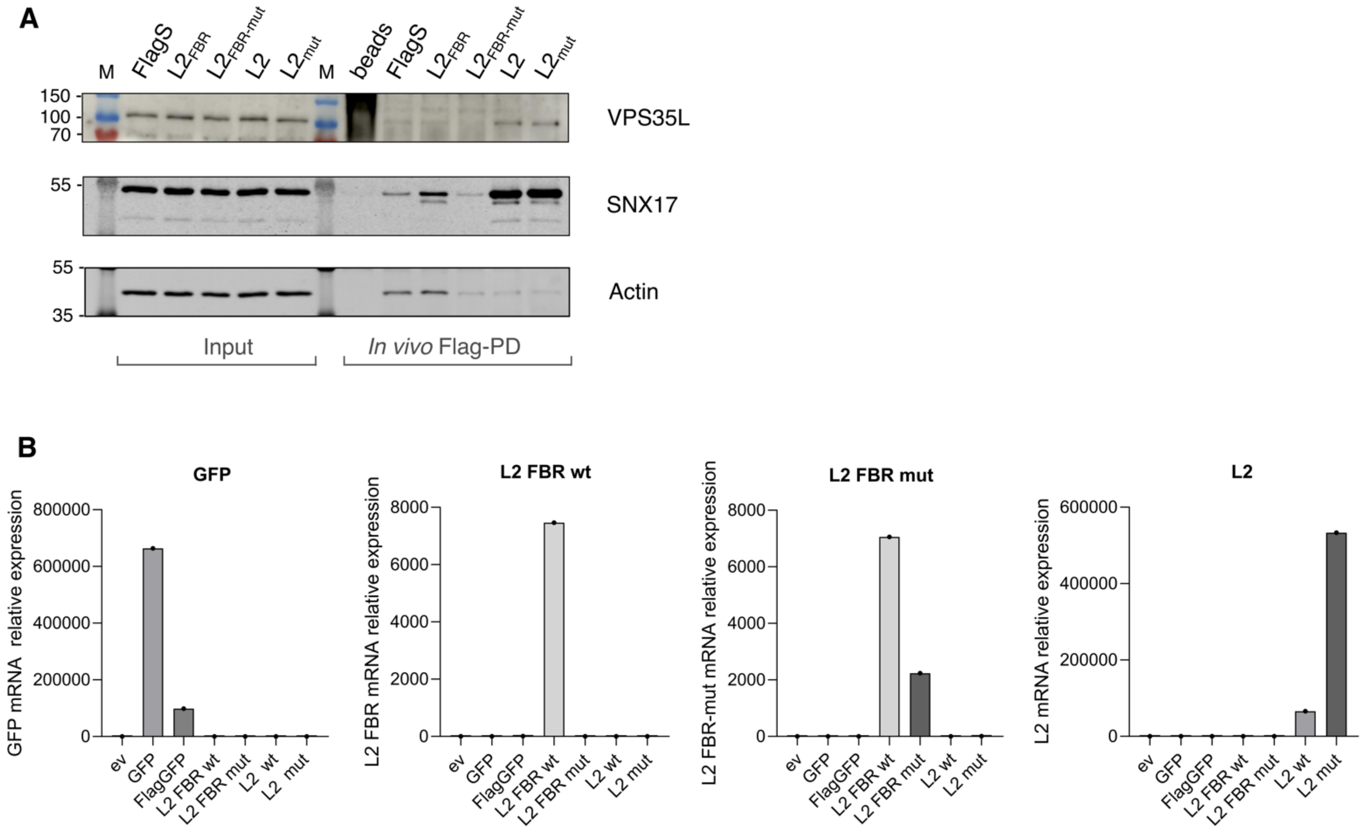

Appendix Figure S3. Detection of VPS35L and SNX17 following Flag pull-down of overexpressed L2 proteins.

(**A**) HEK293T cells expressing either the wild-type or the mutant version (lacking the NPxY motif) of the Flag-tagged L2FBR or L2FL protein were lysed. The supernatants were incubated with M2 anti-Flag beads, followed by pull-down and immunoblot analysis using specific antibodies against VPS35L and SNX17. Cells expressing the empty vector served as a negative pull-down control. Actin was used as loading control for the input samples. Due to non-specific detection by the anti-Flag antibody, mRNA expression levels of the transfected plasmids were quantified by RT-qPCR.

(**B**) Relative transcript expression levels of the GFP vector control, wild-type, and mutant versions (lacking the NPxY motif) of the L2FBR or L2FL proteins were measured by quantitative reverse transcription PCR (RT-qPCR, n=1).

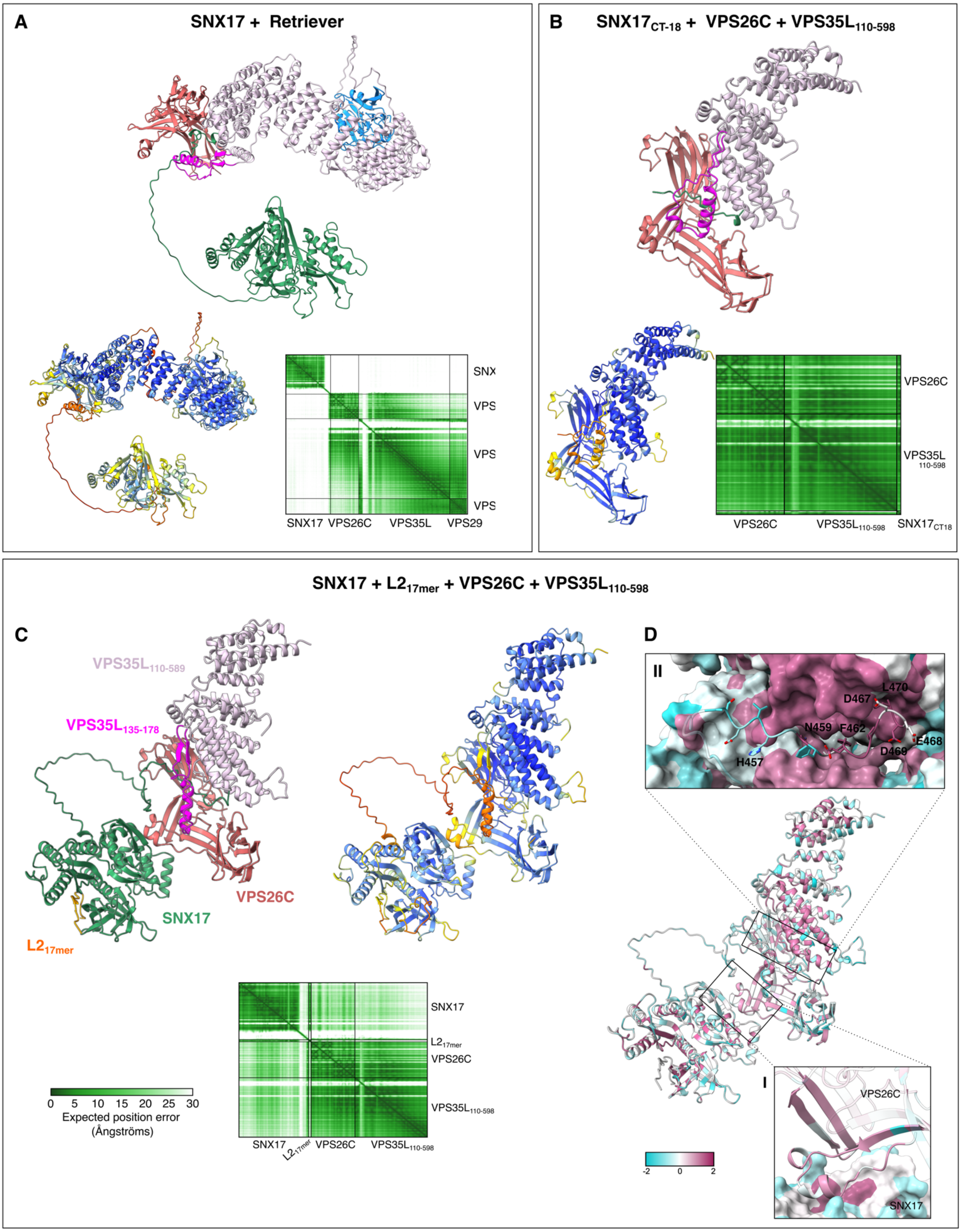

Appendix Figure S4. AlphaFold2-multimer prediction of Retriever-SNX17 interaction.

(**A-C**) Two cartoon representations of AF2-multimer predicted structures of SNX17:Retriever (A), $SNX17_{CT-18}$:VPS26C:$VPS35L_{110-598}$ (B), and SNX17:$L2_{17mer}$:VPS26C:$VPS35L_{110-598}$ (C). In the first representation, SNX17 is colored green, L2 orange, VPS26C red and $VPS35L_{110-598}$ pink. The hinge region ($VPS35L_{135-178}$) is highlighted in magenta. In the second representation, the models are colored according to the pLDDT confidence score (blue, very high confidence pLDDT ≥ 90; cyan, high confidence 70 ≤ pLDDT

< 90; yellow, low confidence 50 ≤ pLDDT < 70; orange, very low confidence pLDDT < 50). The graph at the bottom displays the corresponding PAE (predicted aligned error) plot. The color key of the PAE plot in panel C applies to panels A and B.
(**D**) Depiction of the complex SNX17:L2$_{17mer}$:VPS26C:VPS35L$_{110\text{-}598}$ in cartoon, colored according to the evolutionary conservation calculated by AL2CO (Pei & Grishin, 2001) implemented in ChimeraX using the alignments of SNX17 (Appendix Fig. S5), VPS26C (Appendix Fig. S6) and VPS35L (Appendix Fig. S7), with blue-to-purple color scale indicating variable-to-conserved positions. Zoomed-in view I focused on the interaction interface between VPS26C (in cartoon) and SNX17 (as surface). Zoomed-in view II shows the conservation of the SNX17$_{CT\text{-}18}$ binding pocket, with SNX17 residues as sticks, VPS26C and VPS35L$_{110\text{-}598}$ as surface. The orientation is the same as in Fig. 3B. To facilitate visualization of the binding pocket the residues 135-178 of VPS35L were omitted.

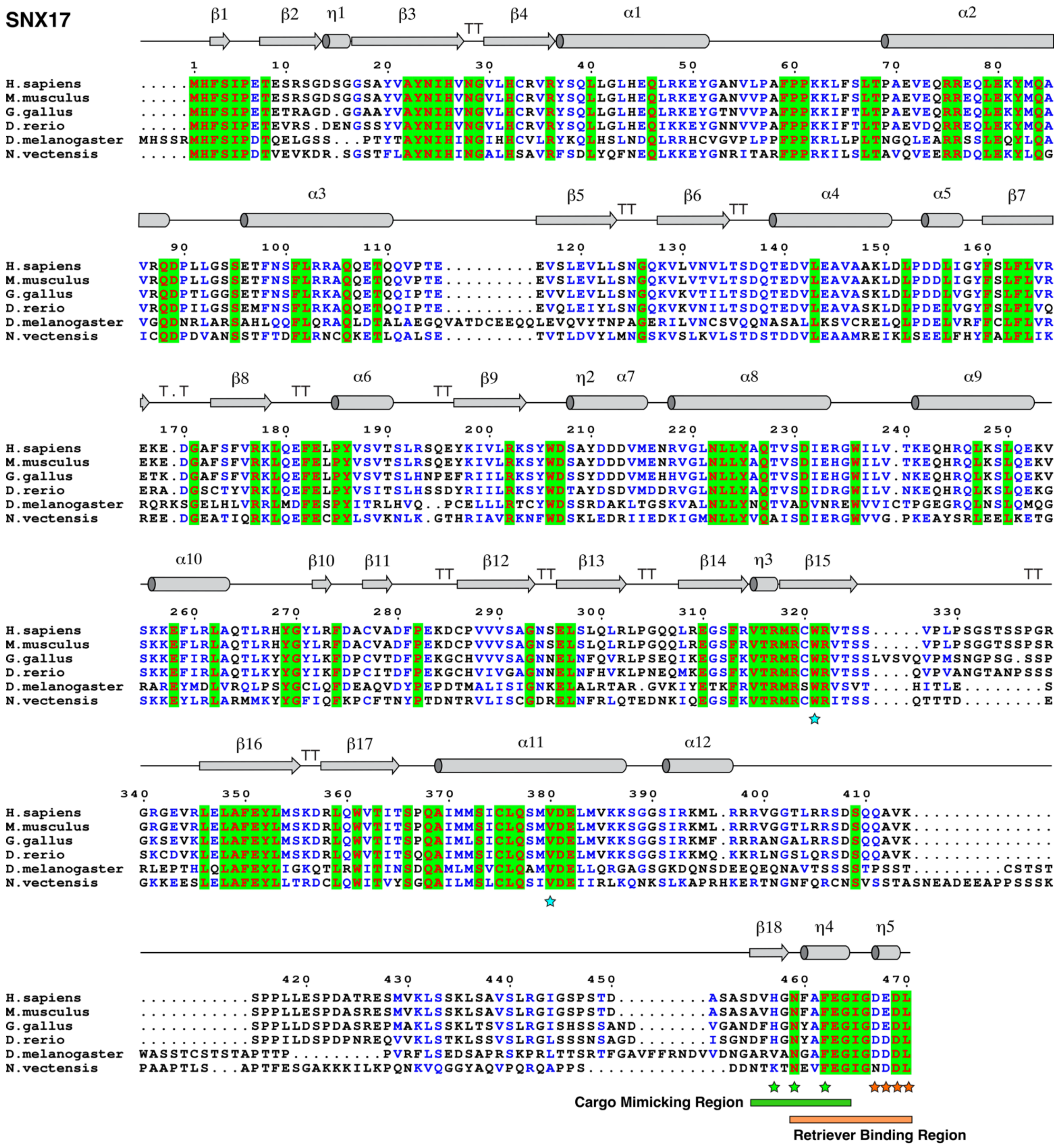

Appendix Figure S5. Conservation of contact residues across SNX17 orthologs.

Structure-based sequence alignment of human SNX17 and orthologs generated with the PROMALS3D server and plotted with ESPript 3. The UniProt accession numbers of the AF2 models used in the sequence alignment are as follows: *Homo sapiens* (Q15036), *Mus musculus* (Q8BVL3), *Gallus gallus* (A0A1D5NVF3), *Danio rerio* (Q5RID7), *Drosophila melanogaster* (Q9VL28), and *Nematostella vectensis* (A7SGL2). The numbering of the residues and secondary structure annotations refer to SNX17 from *Homo sapiens*. a refers to alpha-helices and h to 3_{10} helices (both represented as cylinders). b refers to beta-strands (represented as arrows) and TT to strict b-turns. Invariant residues are colored red on a green background, and conserved residues are in blue. Mutated residues implicated in the autoinhibition mechanism are marked with cyan stars (for the cargo-binding pocket) or green stars (for the cargo-mimicking region). Mutations within the Retriever-binding region are marked with orange stars.

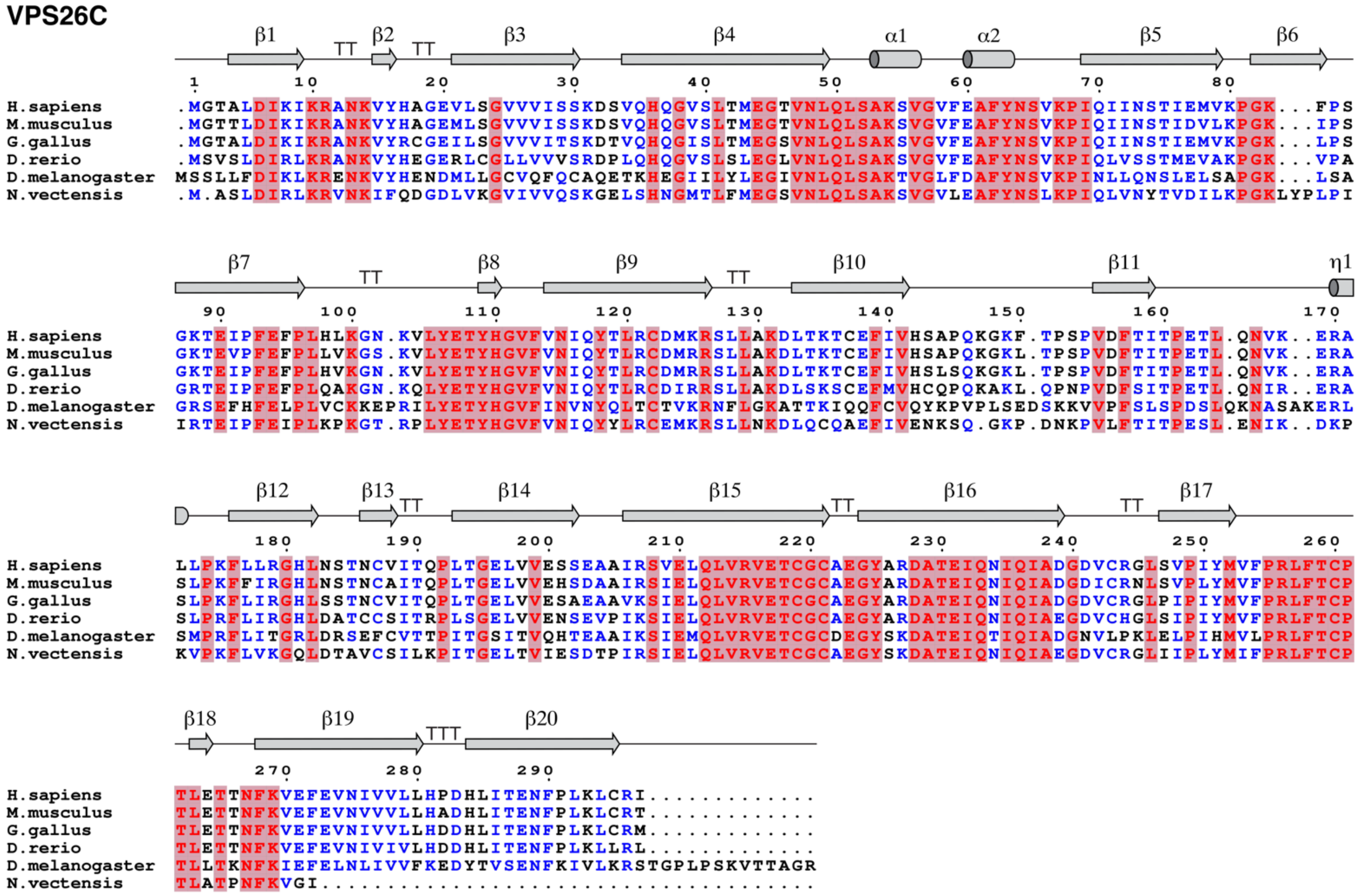

Appendix Figure S6. Conservation of contact residues across VPS26C orthologs.
Structure-based sequence alignment of human VPS26C and orthologs. The alignment was generated as detailed in Appendix Fig. S5. The color code is the same as in Appendix Fig. S5, except for invariant residues, which are highlighted with a red background. The residue numbering and the secondary structure elements shown above the alignment correspond to human VPS26C. The UniProt accession numbers of the AF2 models used in the alignment are as follows: *Homo sapiens* (O14972), *Mus musculus* (O35075), *Gallus gallus* (E1BSI1), *Danio rerio* (Q6DHL2), *Drosophila melanogaster* (Q9VPC3), and *Nematostella vectensis* (A7S8I4).

VPS35L

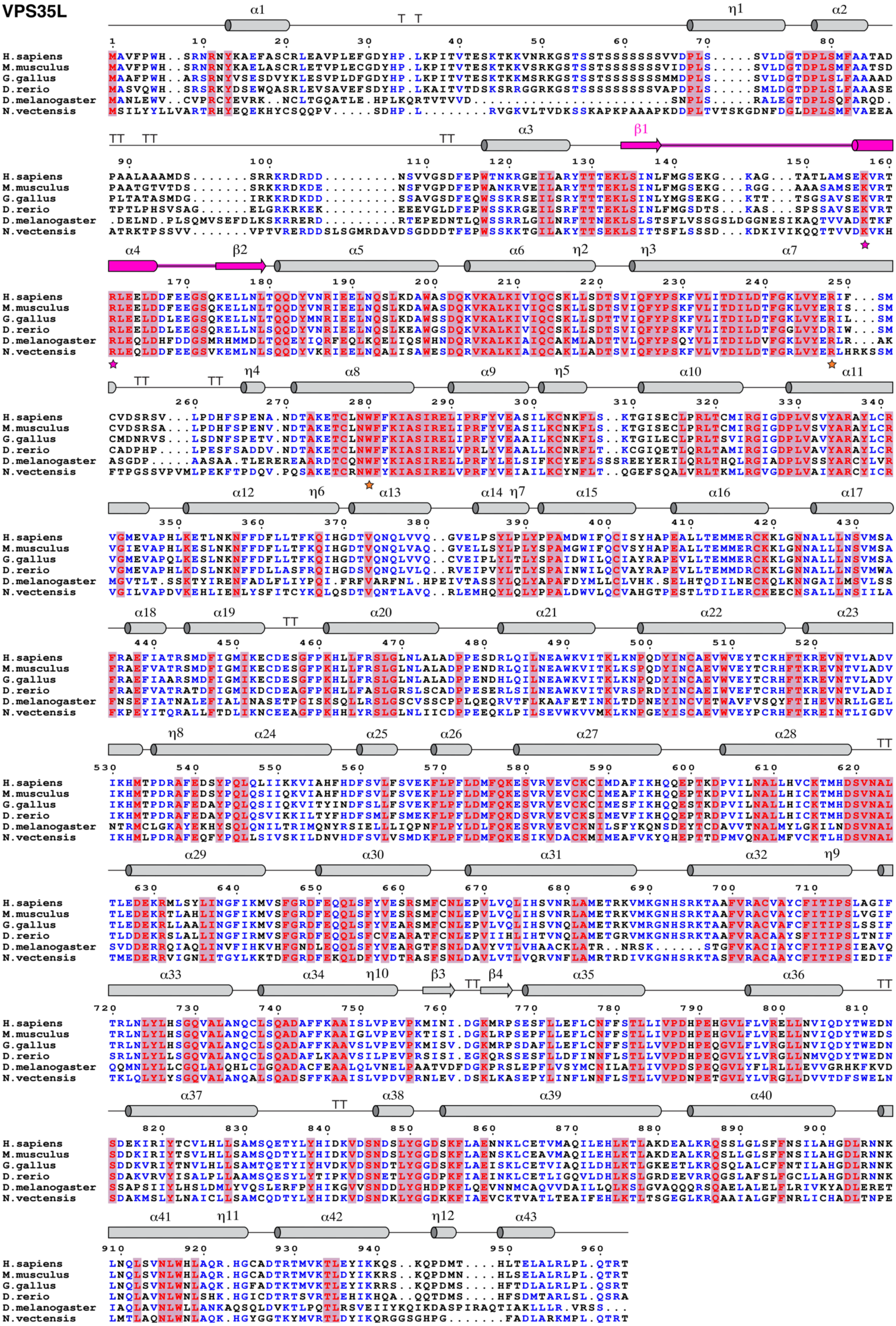
H.sapiens
M.musculus
G.gallus
D.rerio
D.melanogaster
N.vectensis

Appendix Figure S7. Conservation of contact residues across VPS35L orthologs.
Structure-based sequence alignment of human VPS35L with its orthologs. The alignment generation process and the color-coding scheme are the same as those described in Appendix Fig. S5, except for invariant residues, which are highlighted with a pink background. The residue numbering and the secondary structure elements shown above the alignment correspond to human VPS35L. The UniProt accession numbers of the AF2 models used in the alignment are as follows: *Homo sapiens* (Q7Z3J2), *Mus musculus* (Q8BWQ6), *Gallus gallus* (A0A1D5PY39), *Danio rerio* (A4VCH4), *Drosophila melanogaster* (Q9VHM2), and *Nematostella vectensis* (XP_048575353). AF2 models were downloaded from the AlphaFold Protein Structure Database, with the exception of the model for *Nematostella vectensis*, which was obtained using ColabFold. The secondary structure elements of the hinge region are colored in magenta. Residues mutated in this work located in the SNX17 L470 binding pocket are marked with orange stars (R248 and W280), and those within the hinge region are marked with magenta stars (K157 and R161).

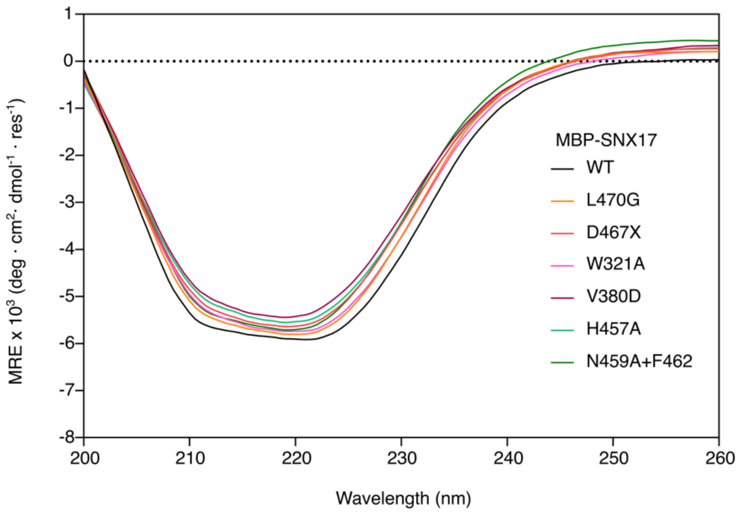

Appendix Figure S8. Conformational properties of MBP-SNX17 mutants.
Far-UV CD spectra of WT MBP-SNX17 and its mutants used in Figs. 3 and 4. MRE, mean residual ellipticity.

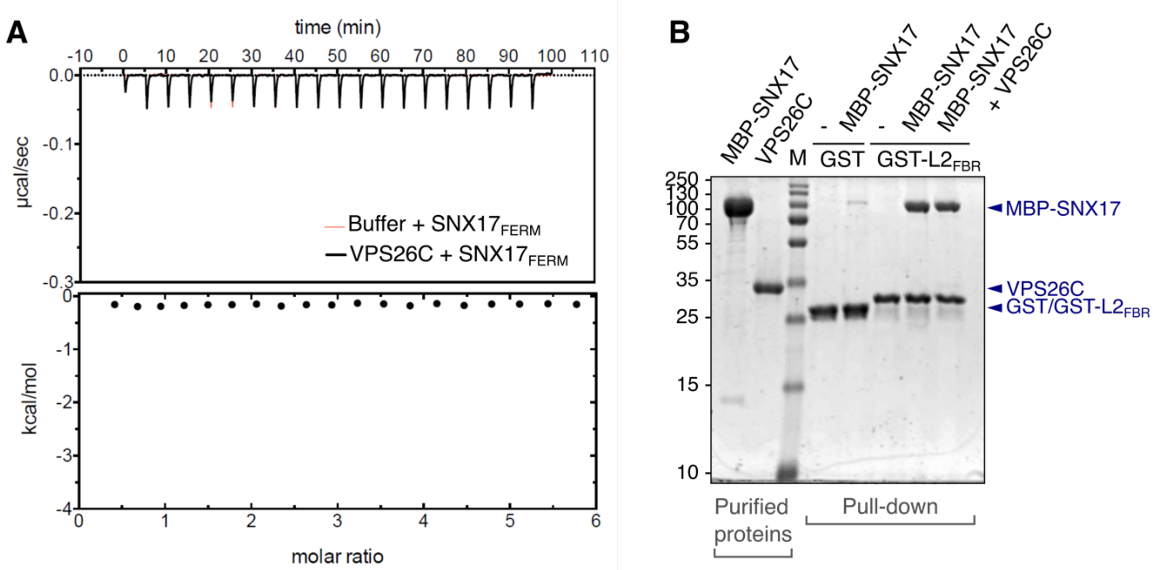

Appendix Figure S9. Analysis of the interaction between VPS26C and SNX17.
(A) ITC assay of 30 µM VPS26C and 300 µM $SNX17_{FERM\text{-}CT}$. No significant interaction was detected, with values comparable to the negative control.
(B) VPS26C was incubated with MBP-SNX17 in the presence of GST-$L2_{FBR}$ in GST pull-down assays. Non-fused GST protein was used as a negative control. Purified proteins and pull-down samples were separated by SDS-PAGE and visualized by Coomassie Blue staining. M, protein marker.

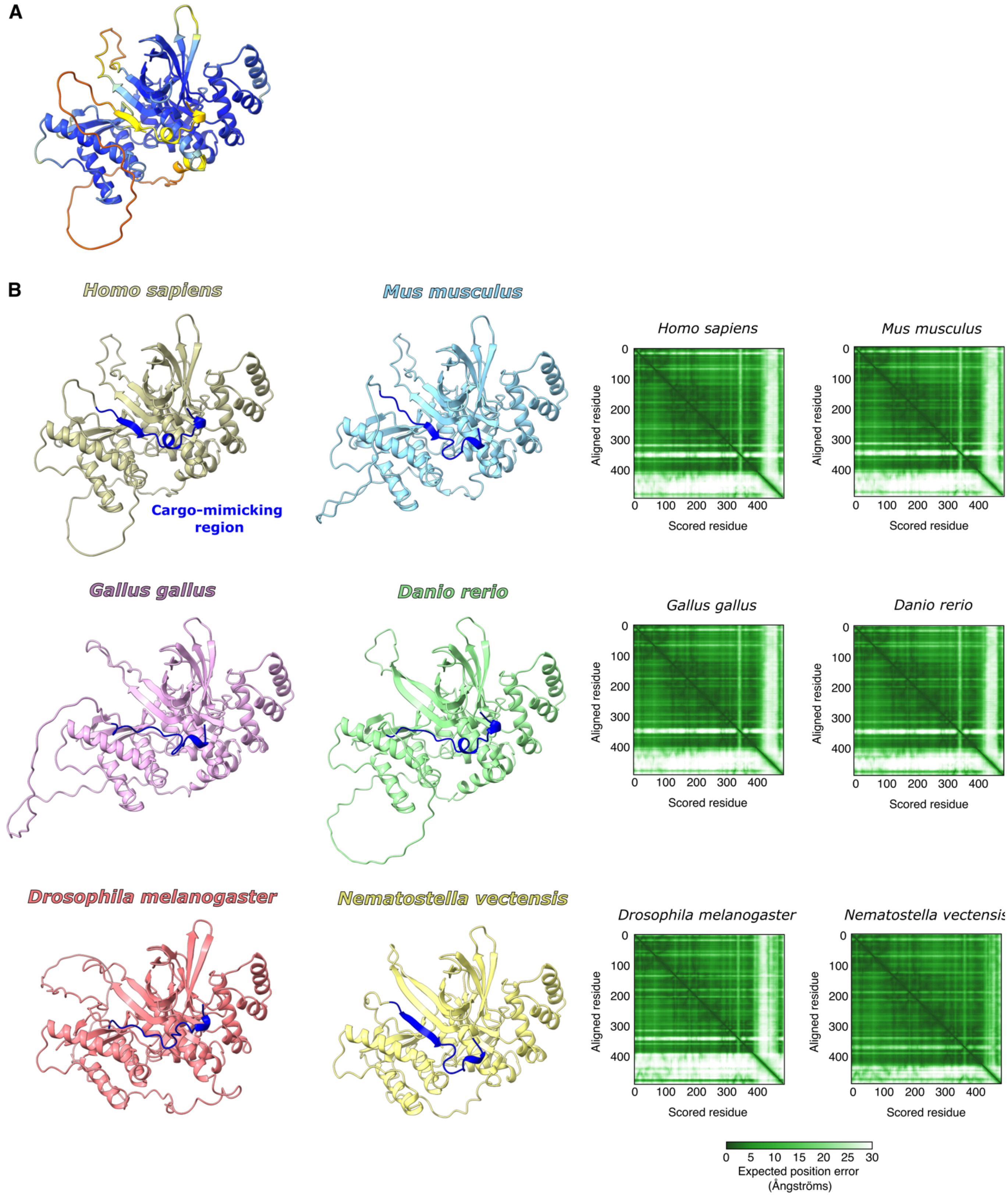

Appendix Figure S10. Conservation of the autoinhibitory mechanism across SNX17 orthologs.
(**A**) Cartoon representation of the AF2 model for human SNX17 (UniProt Q15036), colored by pLDDT confidence score. The prediction confidence color-coding is the same as in Appendix Fig. S4.
(**B**) AF2 models of human SNX17 and orthologs with the corresponding PAE plots. The cargo-mimicking region (from 453 to 470 in the human sequence) is highlighted in dark blue. Models were downloaded from the AlphaFold Protein Structure Database, with the exception of the *Nematostella vectensis* model, which was obtained using ColabFold. The UniProt accession numbers for these proteins are detailed in Appendix Fig. S5.

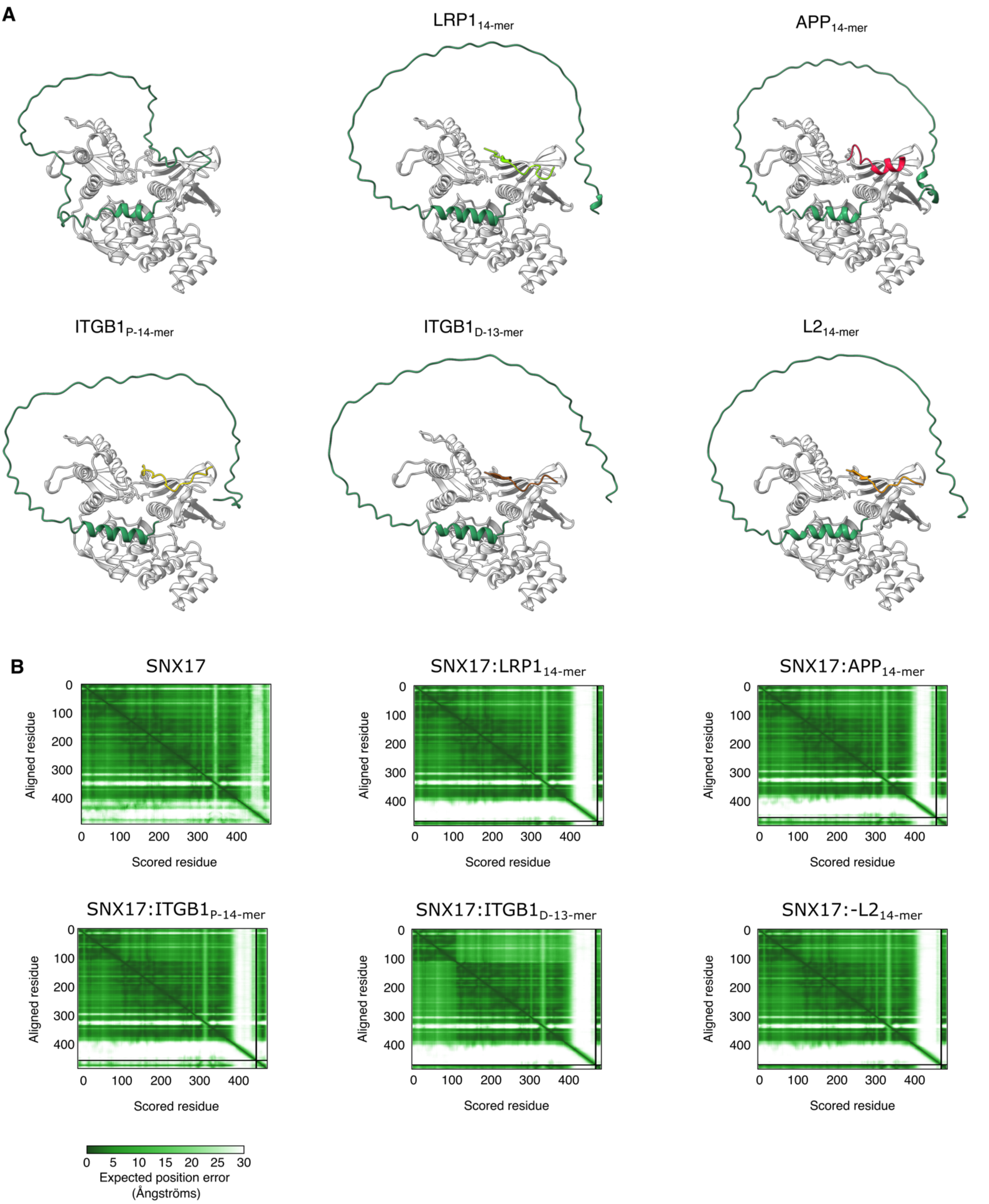

Appendix Figure S11. Assessment of the disruption of SNX17 autoinhibited conformation through cargo binding using AF2-multimer modeling.

(**A**) AF2-multimer prediction of the complex between SNX17 (grey) and the cargo peptides used in fluorescence anisotropy assays: $LRP1_{14\text{-}mer}$ (light green), $APP_{14\text{-}mer}$ (red), $ITG\beta1_{P\text{-}14\text{-}mer}$ (yellow), $ITG\beta1_{D\text{-}13\text{-}mer}$ (brown), and $L2_{14\text{-}mer}$ (orange). Note the displacement of the CT region (green) of SNX17 when cargo peptides are present.

(**B**) PAE plots of the models shown in A.

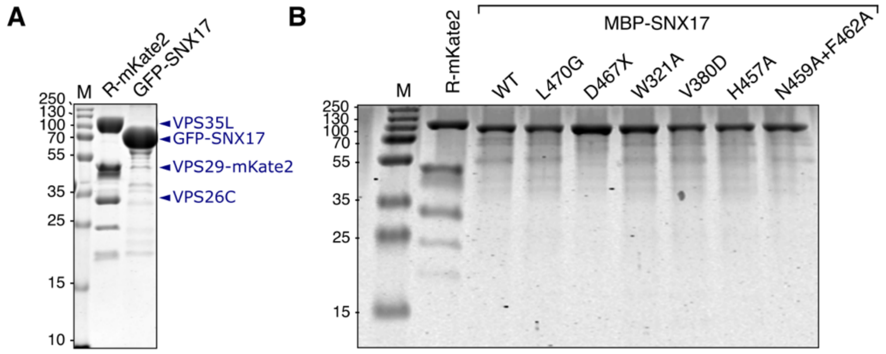

Appendix Figure S12. Purified proteins used for studying SNX17 and Retriever interaction with membranes using GUVs. Coomassie stained SDS-PAGE gels showing the purified proteins Retriever-mKate2 and GFP-SNX17 (**A**), as well as MBP-SNX17 wild-type and mutants (**B**), used for confocal microscopy assays.

Appendix Table S1. SAXS

	**VPS26C**	**Retriever**
**Data collection parameters**		
Instrument	B21 (Diamond Light Source, United Kingdom)	B21 (Diamond Light Source, United Kingdom)
Buffer	50 mM Tris pH 7.5, 150 mM NaCl, 1 mM TCEP	50 mM Tris pH 7.5, 200 mM NaCl, 1 mM TCEP
Beam size at sample (mm)	1.10 x 0.24	1.0 x 0.25
Wavelength (Å)	0.954	0.954
q range (Å^{-1})	0.0045 - 0.34	0.0045 - 0.34
Exposure Time (s/frame)	1.000	3.000
Exposure Temperature (ºC)	25.000	15.000
Concentration used in gel filtration chromatography (mg/ml)	5.000	5.000
**Structural Parameters**		
Forward scattering intensity *[I*(0)] (from Guinier)	0.020	0.128
Forward scattering intensity [*I*(0)] [from *P*(r)]	0.020	0.129
R_g (Å) [from Guinier)	26.626	52.073
R_g (Å) [from *P*(r)]	27.340	53.550
D_{max} (Å)	94.000	215.000
Porod Volume estimate (Å^3) [from *P*(r)]	71164.694	434649.887
Porod Volume estimate corrected (Å^3) [from *P*(r)]	42379.132	241477.927
c^2 [from *P*(r)]	1.035	1.098
Total estimate [from *P*(r)]	0.801	0.614
**Molecular weight determination (kDa)**		
Theoretical molecular weight	33.010	169.830
From volume of correlation (V_c)	31.373	162.499
From Bayesian assessment [credibility interval], probability	33.1 [31.3,34.2], 94.80	169.625 [162.650,194.950], 52.970
From Shape&Size assessment	32.762	193.038
From Porod volume assessment	35.175	200.427
**Software employed**		
Data processing	BioXSTAS RAW 2.2.1	BioXSTAS RAW 2.2.1
Ab initio (analysis)	ATSAS-3.2.0	ATSAS-3.2.0
Validation, averaging and final refinement	DAMMIN refinement of DAMSTART from GASBOR	DAMMIN refinement of DAMSTART from GASBOR
3D structure prediction	AF2	AF2-multimer
Computation of model intensities	CRYSOL	CRYSOL
3D graphics representations	UCSF ChimeraX	UCSF ChimeraX

Appendix Table S2. DNA constructs for recombinant protein production used in this study.

Identifier	Plasmid construct	Source/reference
	pET28-Sumo3	EMBL, Heidelberg
	pGST-P2	(Sheffield et al, 1999)
	pET28NStrep	KP Hopfner Lab (Gene Center)
	pCDH-CMV-fmkate2-EF1-puro	I. Varela Lab (IBBTEC)
	pLIB	(Weissmann et al, 2016)
	pBIG1a	(Weissmann *et al*, 2016)
	pHis-MBP-P4	(Sheffield et al, 1999)
	pBABE-Flag-S vector	(Gammoh *et al*, 2013)
pMLG30	pIA-His	This study
pMLG31	pIA-GST	This study
pMLG32	pIA-His-MBP	This study
pMLG34	pIA-Strep	This study
pMLG73	pIA-TwinStrep	This study
pMLG23	pMK-VPS26C (opt*)	Invitrogen GeneArt
pMLG21	pET28-His-Sumo3-VPS26C (opt)	This study
pMLG6	pGST-VPS29	(Romano-Moreno et al, 2017)
pMLG11	pECE-H2-SNX17	Addgene # 69811
pMLG13	pET28-His-Sumo3-SNX17	This study
pMLG135	pET28-His-Sumo3-$SNX17_{PX}$	This study
pMLG1	pET28-His-Sumo3-$SNX17_{FERM\text{-}CT}$	This study
pMLG14	pET28-His-Sumo3-EGFP-SNX17	This study
pMLG159	pIA-His-MBP-SNX17	This study
pMLG179	pIA-His-MBP-$SNX17_{D467X}$	This study
pMLG178	pIA-His-MBP-$SNX17_{L470G}$	This study
pMLG203	pIA-His-MBP-$SNX17_{W321A}$	This study
pMLG204	pIA-His-MBP-$SNX17_{V380D}$	This study
pMLG205	pIA-His-MBP-$SNX17_{H457A}$	This study
pMLG206	pIA-His-MBP-$SNX17_{N459A+F462A}$	This study
pMLG25	pMK-$LRP1_{ICD}$ (opt)	Invitrogen GeneArt
pMLG18	pIA-GST-$LRP1_{ICD}$ (opt)	This study
pMLG275	pIA-GST-$LRP1_{ICD\text{-}mut}$ (N4470A+Y4473A)	This study
pMLG124	pIA-GST-3C-His_{10}-$LRP1_{ICD}$ (opt)	This study
pMLG165	pIA-His-MBP-His_{10}-$LRP1_{ICD}$ (opt)	This study
pMLG173	pIA-MBP-His_{10}-$LRP1_{ICD}$ (opt)	This study
pMLG48	pGST-APP_{ICD}	A. Hierro Lab (CICbioGUNE)
pMLG49	pIA-GST-APP_{ICD}	This study
pMLG167	gBlock-$ITGB1_{ICD}$	IDT gBlock
pMLG168	pIA-GST-$ITGB1_{ICD}$	This study
pMLG28	pIA-GST-$L2_{FBR}$	This study
pMLG276	pIA-GST-$L2_{FBR\text{-}mut}$ (N254A+Y257A)	This study
pMLG131	pIA-GST-3C-His_{10}-$L2_{FBR}$	This study
pMLG22	pMK-VPS35L (opt)	Invitrogen GeneArt
pMLG44	pIA-His-VPS35L	This study
pMLG37	pLIB-His-VPS35L (opt)	This study
pMLG05	pET28-His-Sumo3-VPS26C	A. Hierro Lab (CICbioGUNE)
pMLG83	pIA-TwinStrep-VPS26C	This study
pMLG84	pLIB-TwinStrep-VPS26C (opt)	This study
pMLG7	pET28-Sumo3-VPS29	A. Hierro Lab (CICbioGUNE)
pMLG39	pLIB-VPS29	This study

pMLG85	pBIG1a-His-VPS35L-TwinStrep-VPS26C-VPS29	This study
pMLG121	pLIB-GST-HRV3C-VPS29	This study
pMLG122	pBIG1a-His-VPS35L-TwinStrep-VPS26C-GST-VPS29	This study
pMLG109	pIA-His-VPS35L$_{110\text{-}963}$	This study
pMLG113	pLIB-His-VPS35L$_{110\text{-}963}$	This study
pMLG118	pBIG1a-His-VPS35L$_{110\text{-}963}$-TwinStrep-VPS26C-VPS29	This study
pMLG107	pIA-His-VPS35L$_{110\text{-}598}$	This study
pMLG111	pLIB-His-VPS35L$_{110\text{-}598}$	This study
pMLG116	pBIG1a-His-VPS35L$_{110\text{-}598}$-TwinStrep-VPS26C-VPS29	This study
pMLG137	pLIB-His-VPS35L$_{1\text{-}598}$	This study
pMLG138	pBIG1a-His-VPS35L$_{1\text{-}598}$-TwinStrep-VPS26C-VPS29	This study
pMLG141	pLIB-His-VPS35L$_{1\text{-}436}$	This study
pMLG144	pBIG1a-His-VPS35L$_{1\text{-}436}$-TwinStrep-VPS26C-VPS29	This study
pMLG151	pBIG1a-His-VPS35L-GST-VPS29	This study
pMLG228	pLIB-HisVPS35L$_{R248E+W280D}$	This study
pMLG231	pBIG1a-HisVPS35L$_{R248E+W280D}$-TwinStrep-VPS26C-VPS29	This study
pMLG229	pLIB-HisVPS35L$_{K157E+\ R161E}$	This study
pMLG232	pBIG1a-HisVPS35L$_{K157E+R161E}$-TwinStrep-VPS26C-VPS29	This study
pMLG15	pET28-Sumo3-VPS29-GFP	This study
pMLG59	pET28-Sumo3-VPS29-mKate2	This study
pMLG115	pLIB-VPS29-mkate2	This study
pMLG120	pBIG1a-His-VPS35L-TwinStrep-VPS26C-VPS29-mKate2	This study
pMLG223	cDNA L2 (HPV16)	Invitrogen GeneArt
pMLG278	pMSCV-FlagS	This study
pMLG279	pMSCV-FlagS-EGFP	This study
pMLG284	pMSCV-FlagS-L2$_{FBR}$	This study
pMLG285	pMSCV-FlagS-L2$_{FBR(N254A+Y257A)}$	This study
pMLG286	pMSCV-FlagS-L2	This study
pMLG287	pMSCV-FlagS-L2$_{(N254A+Y257A)}$	This study

*Opt: Optimized for *E. coli* expression.

Appendix Table S3. DNA oligos used in this study.
ID: pMLG Plasmid identification number.

ID	Amplification	Template	Primer Identifier and sequence, all 5' to 3'
**30**	His-TEV	pET28-Sumo3	088: CCGATGAAACGAGAGAGGATGCTCACGATACGGGTTACTG<br>071: GCCCTGGAAGTACAGGTTTTCGCCGCTGCTGTGATGATGATGATGATG
	His-TEV	pET28-Sumo3	070: GGCGAAAACCTGTACTTCCAGGGCGTGAGCAAGGGCGAGGAGCTG<br>089: CAGTAACCCGTATCGTGAGCATCCTCTCTCGTTTCATCGG
**31**	Insert GST	pGST-P2	072: GAAGGAGATATACCATGGGCAGCAGCATGTCCCCTATACTAGGTTATTGGAAAATTAAGGGCC<br>073: GGAAGTACAGGTTTTCGCCGCTGCTTTTTGGAGGATGGTCGCCACCACC
	Vector	pIA-His	095: GCTGCTGCCCATGGTATATCTCCTTCTTAAAGTTAAAC<br>096: AGCAGCGGCGAAAACCTGTACTTCCAGGGC
**32**	His-MBP-TEV	pHis-MBP-P4	088: CCGATGAAACGAGAGAGGATGCTCACGATACGGGTTACTG<br>074: GGAAGTACAGGTTTTCGCCGCTGCTCCCGAGGTTGTTGTTATTGTTATTGTTGTTGTTG
**34**	Strep	pET28NStrep	088: CCGATGAAACGAGAGAGGATGCTCACGATACGGGTTACTG<br>076: GGAAGTACAGGTTTTCGCCGCTGCTAGCGCCTTTTTCGAACTGCGGGTGG
	Vector	pMLG30	096: AGCAGCGGCGAAAACCTGTACTTCCAGGGC<br>089: CAGTAACCCGTATCGTGAGCATCCTCTCTCGTTTCATCGG
**73**	Half of the vector	pMLG34	089: CAGTAACCCGTATCGTGAGCATCCTCTCTCGTTTCATCGG<br>150:CGGTGGATCAGGTGGAAGTGCATGGTCTCATCCTCAGTTTGAGAAAAGCAGCGGCGAAAACCTGTACTTCCAGG
	Half of the vector	pMLG34	088: CCGATGAAACGAGAGAGGATGCTCACGATACGGGTTACTG<br>151:GGATGAGACCATGCACTTCCACCTGATCCACCGCCAGAACCTCCGCCTTTTTCGAACTGCGGGTGGCTCC
**21**	Insert VPS26C	pMLG23	053: CGTGTTCCAGCAGCAGACCGGTGGAATGGGCACCGCACTGGATATCAAAATCAAACG<br>054: CTCAGTGGTGGTGGTGGTGGTGTTAAATGCGGCACAGTTTCAGCGGAAAGTTTTCGG
	Vector	pET28-Sumo3	002: CACCACCACCACCACCACTGAGATCCGGCTGCTAAC<br>001: TCCACCGGTCTGCTGCTGGAACACGTCGATGGTGTCCTCG
**13**	Insert SNX17	pMLG11	032: CGACGTGTTCCAGCAGCAGACCGGTGGAATGCACTTTTCCATTCCCGAAACCGAGTCCCGC<br>007: CCGGATCTCAGTGGTGGTGGTGGTGGTGTTACAGATCCTCATCTCCAATGCCCTCGAAGG
	Vector	pET28-Sumo3	As in 21.
**135**	Insert $SNX17_{PX}$	pMLG11	032: CGACGTGTTCCAGCAGCAGACCGGTGGAATGCACTTTTCCATTCCCGAAACCGAGTCCCGC<br>184: GATCTCAGTGGTGGTGGTGGTGGTGTTACTCCTGTTGTGCCCGACGCAGGAAAC
	Vector	pET28-Sumo3	As in 21.
**1**	Insert $SNX17_{109C}$	pMLG11	006: CGACGTGTTCCAGCAGCAGACCGGTGGAACACAGCAGGTCCCCACAGAGGAAGTG<br>007: CCGGATCTCAGTGGTGGTGGTGGTGGTGTTACAGATCCTCATCTCCAATGCCCTCGAAGG
	Vector	pET28-Sumo3	As in 21.
**14**	Insert SNX17	pMLG13	007: CCGGATCTCAGTGGTGGTGGTGGTGGTGTTACAGATCCTCATCTCCAATGCCCTCGAAGG<br>034:GGGATCACTCTCGGCATGGACGAGCTCTACAAGGGCGGCGGCGGCAGCATGCACTTTTCCATTCCCGAAACCGAGTCCCGC
	Vector	pET28-Sumo3	002: CACCACCACCACCACCACTGAGATCCGGCTGCTAAC<br>036: CTTGTAGAGCTCGTCCATGCCGAGAGTGATCCCGGCG
**159**	Insert SNX17	pMLG11	007: CCGGATCTCAGTGGTGGTGGTGGTGGTGTTACAGATCCTCATCTCCAATGCCCTCGAAGG<br>208: GACGCGCAGACTAATTCGAGCTCTATGCACTTTTCCATTCCCGAAACCGAGTCC
	Insert MBP	pMLG32	203: GCGAAAACCTGTACTTCCAGGGCATGATCGAAGAAGGTAAACTGGTAATCTGGATTAACG<br>204: AGAGCTCGAATTAGTCTGCGCGTCTTTCAG
	Vector	pMLG30	002: CACCACCACCACCACCACTGAGATCCGGCTGCTAAC<br>094: GCCCTGGAAGTACAGGTTTTCGCCGCTGC
**179**	Insert MBP-$SNX17_{D467X}$	pMLG159	203: GCGAAAACCTGTACTTCCAGGGCATGATCGAAGAAGGTAAACTGGTAATCTGGATTAACG<br>240: GATCTCAGTGGTGGTGGTGGTGGTGTTATCCAATGCCCTCGAAGGCGAAATTGC
	Vector	pMLG30	As in 159.
**178**	Insert MBP-$SNX17_{L470G}$	pMLG159	203: GCGAAAACCTGTACTTCCAGGGCATGATCGAAGAAGGTAAACTGGTAATCTGGATTAACG<br>239: GATCTCAGTGGTGGTGGTGGTGGTGTTAACCATCCTCATCTCCAATGCCCTCGAAGG
	Vector	pMLG30	As in 159.
**203**	pIA-HisMBP-$SNX17_{W321A}$_1	pMLG159	246: GTCACCCGCATGCGATGCGCGCGGGTCACCTCCTCTGTAC<br>245: CAGTAACCCGTATCGTGAGCATCCTCTCTC
	pIA-HisMBP-$SNX17_{W321A}$_2	pMLG159	244: GAGAGAGGATGCTCACGATACGGGTTACTG<br>247: GTACAGAGGAGGTGACCCGCGCGCATCGCATGCGGGTGAC
**204**	pIA-HisMBP-$SNX17_{V380D}$_1	pMLG159	248: GCAGTCCATGGATGATGAACTGATGGTGAAGAAATCTGGC<br>245: CAGTAACCCGTATCGTGAGCATCCTCTCTC
	pIA-HisMBP-$SNX17_{V380D}$_2	pMLG159	244: GAGAGAGGATGCTCACGATACGGGTTACTG<br>249: CACCATCAGTTCATCATCCATGGACTGCAAGCAGATGCTC
**205**	pIA-HisMBP-$SNX17_{H457A}$_1	pMLG159	250: TGCCAGTGATGTCGCCGGCAATTTCGCCTTCGAGG<br>245: CAGTAACCCGTATCGTGAGCATCCTCTCTC
	pIA-HisMBP-$SNX17_{H457A}$_2	pMLG159	244: GAGAGAGGATGCTCACGATACGGGTTACTG<br>251: AAGGCGAAATTGCCGGCGACATCACTGGCACTGGCATCTG
**206**	pIA-HisMBP-$SNX17_{N459A+F462A}$_1	pMLG159	252: GGTGCTTTCGCTGCTGAGGGCATTGGAGATGAGGATCTG<br>245: CAGTAACCCGTATCGTGAGCATCCTCTCTC

	pIA-HisMBP-SNX17 $_{N459A+F462A_}$2	pMLG159	244: GAGAGAGGATGCTCACGATACGGGTTACTG<br>253: CCCTCAGCAGCGAAAGCACCGTGGACATCACTGGCAC
**18**	Insert LRP1 $_{ICD}$	pMLG25	107: GGCGAAAACCTGTACTTCCAGGGCTATAAACGTCGTGTTCAGGGTGCAAAAGG<br>108: GATCTCAGTGGTGGTGGTGGTGGTGTTATGCCAGCGGATCACCAATTTCATCTTC
	Vector	pMLG31	002: CACCACCACCACCACCACTGAGATCCGGCTGCTAAC<br>094: GCCCTGGAAGTACAGGTTTTCGCCGCTGC
**275**	pIA-GST-LRP1 $_{ICD-mut_}$1	pMLG18	339: GAAATTGGTGCCCCGACCGCCAAAATGTATGAAGGTGGTGAACC<br>245: CAGTAACCCGTATCGTGAGCATCCTCTCTC
	pIA-GST-LRP1 $_{ICD-mut_}$2	pMLG18	244: GAGAGAGGATGCTCACGATACGGGTTACTG<br>340: CATACATTTTGGCGGTCGGGGCACCAATTTCCACATTCATTGC
**124**	Insert His $_{10}$-LRP1 $_{ICD}$	pMLG18	175: CACCATCATCATCACCATCATCATCATCATTATAAACGTCGTGTTCAGGGTGCAAAAGG<br>089: CAGTAACCCGTATCGTGAGCATCCTCTCTCGTTTCATCGG
	Vector pIA-GST-HRV3C	pMLG18	088: CCGATGAAACGAGAGAGGATGCTCACGATACGGGTTACTG<br>174:ATGATGATGATGATGGTGATGATGATGGTGTGGACCTTGAAACAAAACTTCTAAGCCGCTGCTTTTTG GAGGATGGTCGCC
**165**	Insert His $_{10}$-LRP1 $_{ICD}$	pMLG124	212: GGCGAAAACCTGTACTTCCAGGGCCACCATCATCATCACCATCATCATCATC<br>108: GATCTCAGTGGTGGTGGTGGTGGTGTTATGCCAGCGGATCACCAATTTCATCTTC
	Vector	pMLG32	002: CACCACCACCACCACCACTGAGATCCGGCTGCTAAC<br>094: GCCCTGGAAGTACAGGTTTTCGCCGCTGC
**173**	Vector pIA-MBP-TEV-His $_{10}$-LRP1 $_{ICD_}$1	pMLG165	088: CCGATGAAACGAGAGAGGATGCTCACGATACGGGTTACTG<br>216: GTAGTACGACATATGTATTCTCCTTCTTAAAGTTAAAC
	Vector pIA-MBP-TEV-His $_{10}$-LRP1 $_{ICD_}$2	pMLG165	089: CAGTAACCCGTATCGTGAGCATCCTCTCTCGTTTCATCGG<br>215:GTTTAACTTTAAGAAGGAGAATACATATGTCGTACTACATGATCGAAGAAGGTAAACTGGTAATCTGG
**49**	Insert APP $_{ICD}$	pMLG48	102: GGCGAAAACCTGTACTTCCAGGGCAAGAAAAAGCAGTACACCTCTATCCACCACGG<br>103: GATCTCAGTGGTGGTGGTGGTGGTGTTAGTTCTGCATCTGTTCGAAGAATTTGTAGGTCGGG
	Vector	pMLG31	As in 18.
**168**	Insert ITGB1 $_{ICD}$	pMLG167	214: CACCATCATCATCACCATCATCATCATCATAAACTGCTCATGATTATCCACGACCGTCGT
	Vector	pMLG31	As in 18.
**28**	Insert L2 $_{FBR}$	Overlapping oligos	132: AGCAGCGGCGAAAACCTGTACTTCCAGGGCGATCCG<br>129: GATTATCATAGGTGATCAGTTTGGTCGGGGTGGTCACAAAGGCCGGATCGCCCTGGAAGT<br>84: ACCAAACTGATCACCTATGATAATCCGGCGTACGAAGGCATTGATGTGGATAACACCCTG<br>130: GATCTCAGTGGTGGTGGTGGTGGTGTTAGAAGTACAGGGTGTTATCCACATCAATG
	Vector	pMLG31	As in 18.
**276**	pIA-GST-L2 $_{FBR-mut_}$1	pMLG28	341: CCTATGATGCTCCGGCGGCAGAAGGCATTGATGTGGATAAC<br>245: CAGTAACCCGTATCGTGAGCATCCTCTCTC
	pIA-GST-L2 $_{FBR-mut_}$2	pMLG28	244: GAGAGAGGATGCTCACGATACGGGTTACTG<br>342: CAATGCCTTCTGCCGCCGGAGCATCATAGGTGATCAGTTTGGTCG
**131**	Insert GST and L2 $_{FBR}$	pMLG126	072: GAAGGAGATATACCATGGGCAGCAGCATGTCCCCTATACTAGGTTATTGGAAAATTAAGGGCC<br>110: GGGTTATGCTAGTTATTGCTCAGCGGTGGC
	Vector	pMLG31	167: GCCACCGCTGAGCAATAACTAGCATAACCC<br>095: GCTGCTGCCCATGGTATATCTCCTTCTTAAAGTTAAAC
**44**	Insert VPS35L	pMLG22	065: GGCGAAAACCTGTACTTCCAGGGCATGGCAGTTTTTCCGTGGCATAGCC<br>066: GATCTCAGTGGTGGTGGTGGTGGTGTTAGGTACGGGTCTGCAGCGGC
	Vector	pMLG30	As in 159.
**37**	Insert His-VPS35L	pMLG44	063: CCACCATCGGGCGCGGATCCAATGGGCAGCAGCCATCATCATCATCATC<br>118:GGTATGGCTGATTATGATCCTCTAGTACTTCTCGACAAGCTTTTAGGTACGGGTCTGCAGCGGCAGG C
	Vector	pLIB	115: GGATCCGCGCCCGATGGTGGG<br>116: AAGCTTGTCGAGAAGTACTAGAGGATCATAATCAGCCATACCAC
**83**	Vector	pMLG73	002: CACCACCACCACCACCACTGAGATCCGGCTGCTAAC<br>094: GCCCTGGAAGTACAGGTTTTCGCCGCTGC
	Insert VPS26C	pMLG05	013: CGGATCTCAGTGGTGGTGGTGGTGGTGTTATATCCTGCAGAGCTTCAGCGGGAAGTTCTCC<br>154: GGCGAAAACCTGTACTTCCAGGGCATGGGGACCGCCCTGGACATCAAGATTAAAAGAG
**84**	Insert VPS26C	pMLG83	152: CCACCATCGGGCGCGGATCCATGGCTAGCTGGAGCCACCCGCAG<br>153:GGTATGGCTGATTATGATCCTCTAGTACTTCTCGACAAGCTTCGGATCTCAGTGGTGGTGGTGGTGGT G
**39**	Insert VPS29	pMLG7	061: CCACCATCGGGCGCGGATCCAATGTTGGTGTTGGTATTAGGAGATCTGCAC<br>062:GATGATGTGAAAGTAGAACGAATCGAATACAAAAAACCTTAAAAGCTTGTCGAGAAGTACTAGAGGA
**85**	Insert VPS35L	pMLG37	055: AACGCTCTATGGTCTAAAGATTTAAATCGACCTACTCCGGAATATTAATAGATC<br>056: AAACGTGCAATAGTATCCAGTTTATTTAAATGGTTATGATAGTTATTGCTCAGCG
	Insert VPS26	pMLG84	057: AAACTGGATACTATTGCACGTTTAAATCGACCTACTCCGGAATATTAATAGATC<br>058: AAACATCAGGCATCATTAGGTTTATTTAAATGGTTATGATAGTTATTGCTCAGCG
	Insert VPS29	pMLG39	059: AAACCTAATGATGCCTGATGTTTAAATCGACCTACTCCGGAATATTAATAGATC<br>060: AACCCCGATTAAGATATAGATTTATTTAAATGGTTATGATAGTTATTGCTCAGCG
**121**	Insert GST	pGEX-6P-1-H(RBS)	172: CCCACCATCGGGCGCGGATCCAATGTCCCCTATACTAGGTTATTGGAAAATTAAGGGCC<br>173:GTGCAGATCTCCTAATACCAACACCAACATGGGCCCCTGGAACAGAACTTCCAGATCCGATTTTGG
	Vector	pMLG39	171: ATGTTGGTGTTGGTATTAGGAGATCTGCACATCCCACACCGG<br>115: GGATCCGCGCCCGATGGTGGG
**122**	Insert VPS35L	pMLG37	As in 85.
	Insert VPS26C	pMLG84	As in 85.
	Insert GST-VPS29	pMLG121	059: AAACCTAATGATGCCTGATGTTTAAATCGACCTACTCCGGAATATTAATAGATC<br>060: AACCCCGATTGAGATATAGATTTATTTAAATGGTTATGATAGTTATTGCTCAGCG

**109**	Insert VPS35L$_{110-963}$	pMLG22	119: GGCGAAAACCTGTACTTCCAGGGCGTGGGTAGCGATTTTGAACCGTGGACC<br>066: GATCTCAGTGGTGGTGGTGGTGGTGTTAGGTACGGGTCTGCAGCGGC
	Vector	pMLG30	As in 159
**113**	Insert VPS35L$_{110-963}$	pMLG109	063: CCACCATCGGGCGCGGATCCAATGGGCAGCAGCCATCATCATCATCATC<br>153:<br>GGTATGGCTGATTATGATCCTCTAGTACTTCTCGACAAGCTTCGGATCTCAGTGGTGGTGGTGGTGGTG
**118**	Insert VPS35L$_{110-963}$	pMLG113	055: AACGCTCTATGGTCTAAAGATTTAAATCGACCTACTCCGGAATATTAATAGATC<br>056: AAACGTGCAATAGTATCCAGTTTATTTAAATGGTTATGATAGTTATTGCTCAGCG
	Insert VPS26C	pMLG84	As in 85.
	Insert VPS29	pMLG39	As in 85.
**107**	Insert VPS35L$_{110-598}$	pMLG22	119: GGCGAAAACCTGTACTTCCAGGGCGTGGGTAGCGATTTTGAACCGTGGACC<br>126:GATCTCAGTGGTGGTGGTGGTGGTGTTATTGCTGATGTTTGATAAAGGCGTCCATAATACATTTGCAC
	Vector	pMLG30	As in 159
**111**	Insert VPS35L$_{110-598}$	pMLG107	063: CCACCATCGGGCGCGGATCCAATGGGCAGCAGCCATCATCATCATCATC<br>153:<br>GGTATGGCTGATTATGATCCTCTAGTACTTCTCGACAAGCTTCGGATCTCAGTGGTGGTGGTGGTGGTG
**116**	Insert VPS35L$_{110-598}$	pMLG111	055: AACGCTCTATGGTCTAAAGATTTAAATCGACCTACTCCGGAATATTAATAGATC<br>056: AAACGTGCAATAGTATCCAGTTTATTTAAATGGTTATGATAGTTATTGCTCAGCG
	Insert VPS26C	pMLG84	As in 85.
	Insert VPS29	pMLG39	As in 85.
**137**	Insert VPS35L$_{1-598}$	pMLG37	063: CCACCATCGGGCGCGGATCCAATGGGCAGCAGCCATCATCATCATCATC<br>126:GATCTCAGTGGTGGTGGTGGTGGTGTTATTGCTGATGTTTGATAAAGGCGTCCATAATACATTTGCAC
	Insert VPS35L$_{1-598}$	PCR VPS35L$_{1-598}$	063: CCACCATCGGGCGCGGATCCAATGGGCAGCAGCCATCATCATCATCATC<br>153:GGTATGGCTGATTATGATCCTCTAGTACTTCTCGACAAGCTTCGGATCTCAGTGGTGGTGGTGGTGGT<br>G
	Vector	pLIB	115: AAGCTTGTCGAGAAGTACTAGAGGATCATAATCAGCCATACCAC<br>116: GGATCCGCGCCCGATGGTGGG
**138**	Insert VPS35L$_{1-598}$	pMLG137	055: AACGCTCTATGGTCTAAAGATTTAAATCGACCTACTCCGGAATATTAATAGATC<br>056: AAACGTGCAATAGTATCCAGTTTATTTAAATGGTTATGATAGTTATTGCTCAGCG
	Insert VPS26C	pMLG84	As in 85.
	Insert VPS29	pMLG39	As in 85.
**141**	Insert VPS35L $_{1-436}$	pMLG37	063: CCACCATCGGGCGCGGATCCAATGGGCAGCAGCCATCATCATCATCATC<br>196: GATCTCAGTGGTGGTGGTGGTGGTGTTAACGAAATGCGCTCATAACGCTATTCAGCAGC
	Insert VPS35L $_{1-436}$	PCR VPS35L$_{1-436}$	063: CCACCATCGGGCGCGGATCCAATGGGCAGCAGCCATCATCATCATCATC<br>153:<br>GGTATGGCTGATTATGATCCTCTAGTACTTCTCGACAAGCTTCGGATCTCAGTGGTGGTGGTGGTGGTG
	Vector	pLIB	As in 137.
**144**	Insert VPS35L $_{1-436}$	pMLG141	055: AACGCTCTATGGTCTAAAGATTTAAATCGACCTACTCCGGAATATTAATAGATC<br>056: AAACGTGCAATAGTATCCAGTTTATTTAAATGGTTATGATAGTTATTGCTCAGCG
	Insert VPS26C	pMLG84	As in 85.
	Insert VPS29	pMLG39	As in 85.
**151**	Insert VPS35L	pMLG37	As in 85.
	Insert GST-VPS29	pMLG121	057: AAACTGGATACTATTGCACGTTTAAATCGACCTACTCCGGAATATTAATAGATC<br>060: AACCCCGATTGAGATATAGATTTATTTAAATGGTTATGATAGTTATTGCTCAGCG
**228**	Insert VPS35L$_{R248E+W280D}$	pMLG37	274: CCTTTGGTAAACTGGTGTATGAAGAAATTTTTAGCATGTGTGTTGATAGC<br>273: CGAATGCTGGCAATTTTGAAAAAATCATTCAGGCAGGTTTCTTTGG
	Vector pLIB-His-VPS35L	pMLG37	272: CCAAAGAAACCTGCCTGAATGATTTTTTCAAAATTGCCAGCATTCG<br>275: GCTATCAACACACATGCTAAAAATTTCTTCATACACCAGTTTACCAAAGG
**231**	Insert VPS35L $_{R248E+W280D}$	pMLG228	055: AACGCTCTATGGTCTAAAGATTTAAATCGACCTACTCCGGAATATTAATAGATC<br>056: AAACGTGCAATAGTATCCAGTTTATTTAAATGGTTATGATAGTTATTGCTCAGCG
	Insert VPS26C	pMLG84	As in 85.
	Insert VPS29	pMLG39	As in 85.
**229**	Insert VPS35L$_{K157E+R161E}$	pMLG37	280: CCCTGGCAATGAGTGAAGAAGTTCGTACAGAACTGGAAGAACTGGATGATTTTG<br>093: CTCATGGTTATGGCAGCACTGCATAATTCTCTTACTGTC
	Vector pLIB-His-VPS35L	pMLG37	092: GACAGTAAGAGAATTATGCAGTGCTGCCATAACCATGAG<br>281: CAAAATCATCCAGTTCTTCCAGTTCTGTACGAACTTCTTCACTCATTGCCAGGG
**232**	Insert VPS35L$_{K157E+R161E}$	pMLG229	055: AACGCTCTATGGTCTAAAGATTTAAATCGACCTACTCCGGAATATTAATAGATC<br>056: AAACGTGCAATAGTATCCAGTTTATTTAAATGGTTATGATAGTTATTGCTCAGCG
	Insert VPS26C	pMLG84	As in 85.
	Insert VPS29	pMLG39	As in 85.
**15**	Insert VPS29	pMLG6	033: ATCGACGTGTTCCAGCAGCAGACCGGTGGAATGTTGGTGTTGGTATTAGGAGATCTGCACA<br>035:GAACAGCTCCTCGCCCTTGCTCACGGAGCTGCCGCCGCCGCCAGGTTTTTTGTATTCGATTCGTTCT<br>ACTTTCACATC
	Vector	pET28-Sumo3	037: TCCACCGGTCTGCTGCTGGAACACGTCGATGGTGTCC<br>038: TCCGTGAGCAAGGGCGAGGAGCTGTTCACCGGG
**59**	Insert mkate2	pCDH-CMV-fmkate2-EF1-puro	127: GGCGGCGGCGGCAGCTCCATGGTGAGCGAGCTGATTAAGGAGAACATG<br>128: GATCTCAGTGGTGGTGGTGGTGGTGTTATCTGTGCCCCAGTTTGCTAGGGAGG
	Vector	pMLG15	002: CACCACCACCACCACCACTGAGATCCGGCTGCTAAC

			131: GGAGCTGCCGCCGCCGCC
	Vector	pMLG7	002: CACCACCACCACCACCACTGAGATCCGGCTGCTAAC<br>143:GCTCGCTCACCATGGAGCTGCCGCCGCCGCCAGGTTTTTTGTATTCGATTCGTTCTACTTTCACATCATCTCC
**115**	Insert VPS29 and mkate2	pMLG59	061: CCACCATCGGGCGCGGATCCAATGTTGGTGTTGGTATTAGGAGATCTGCAC<br>153:GGTATGGCTGATTATGATCCTCTAGTACTTCTCGACAAGCTTCGGATCTCAGTGGTGGTGGTGGTGGTG
**120**	Insert VPS29-mkate2	pMLG115	059: AAACCTAATGATGCCTGATGTTTAAATCGACCTACTCCGGAATATTAATAGATC<br>060:AACCCCGATTGAGATATAGATTTATTTAAATGGTTATGATAGTTATTGCTCAGCG
	Insert VPS35L	pMLG37	As in 85.
	Insert VPS26C	pMLG84	As in 85.
**278**	Vector	MSCV-puro BglII-XhoI	
	Insert FlagS	pBABE-Flag-S vector	
**279**	Vector	pMLG278 XhoI-EcoRI	
	Insert EGFP		
**284**	Vector	pMLG278 XhoI+EcoRI	
	Insert L2$_{FBR}$	pMLG28	361: GGACAGCGGAGGTGGAGGTACTAGTCCCGATCCGGCCTTTGTGACC<br>344: GAAAAGCGCCTCCCCTACCCGGTAGTTAGAAGTACAGGGTGTTATCCACATC
**285**	Vector	pMLG278 XhoI+EcoRI	
	Insert L2$_{FBR(N254A+Y257A)}$	pMLG276	As in 284.
**286**	Vector	pMLG278 XhoI+EcoRI	
	Insert L2	pMLG223	362: GGACAGCGGAGGTGGAGGTACTAGTCCCATGCGTCATAAACGCAGCGC<br>346: GAAAAGCGCCTCCCCTACCCGGTAGTTAGGCAGCAAGAGAGACGTCTGAG
**287**	Vector	pMLG278 XhoI+EcoRI	
	Mutagenesis of L2$_{(N254A+Y257A)}$_1	pMLG223	345: GGACAGCGGGGTGGAGGTACTAGTCATGCGTCATAAACGCAGCGC<br>348: CCCTTCAGCGGCTGGAGCATCATAGGTAATTAACTTGGTAGGAGTTG
	Mutagenesis of L2$_{(N254A+Y257A)}$_2	pMLG223	347: CCTATGATGCTCCAGCCGCTGAAGGGATTGACGTTGATAACACC<br>346: GAAAAGCGCCTCCCCTACCCGGTAGTTAGGCAGCAAGAGAGACGTCTGAG

Appendix Table S4. Sequences of recombinant proteins used in this work.

ID: This column lists the pMLG plasmid identification numbers.

The listed sequences correspond to the cloned proteins before the removal of the affinity tag by protease cleavage. The tag sequences are annotated in blue, the protease recognition sites in orange, the extra sequences resulting from cloning in green and mutations from the wild-type sequence in red.

ID	Sequence
31	> GST<br>MGSSMSPILGYWKIKGLVQPTRLLLEYLEEKYEEHLYERDEGDKWRNKKFELGLEFPNLPYYIDGDVKLTQSMAIIRYI<br>ADKHNMLGGCPKERAEISMLEGAVLDIRYGVSRIAYSKDFETLKVDFLSKLPEMLKMFEDRLCHKTYLNGDHVTHPDF<br>MLYDALDVVLYMDPMCLDAFPKLVCFKKRIEAIPQIDKYLKSSKYIAWPLQGWQATFGGGDHPPK
21	> His-Sumo3-VPS26C<br>MGSSHHHHHHSSGLVPRGSHMNDHINLKVAGQDGSVVQFKIKRHTPLSKLMKAYCERQGLSMRQIRFRFDGQPINE<br>TDTPAQLEMEDEDTIDVFQQQTGGMGTALDIKIKRANKVYHAGEVLSGVVVISSKDSVQHQGVSLTMEGTVNLQLSAK<br>SVGVFEAFYNSVKPIQIINSTIEMVKPGKFPSGKTEIPFEFPLHLKGNKVLYETYHGVFVNIQYTLRCDMKRSLLAKDLTK<br>TCEFIVHSAPQKGKFTPSPVDFTITPETLQNVKERALLPKFLLRGHLNSTNCVITQPLTGELVVESSEAAIRSVELQLVR<br>VETCGCAEGYARDATEIQNIQIADGDVCRGLSVPIYMVFPRLFTCPTLETTNFKVEFEVNIVVLLHPDHLITENFPLKLCR<br>I
6	> GST-TEV-VPS29<br>MSPILGYWKIKGLVQPTRLLLEYLEEKYEEHLYERDEGDKWRNKKFELGLEFPNLPYYIDGDVKLTQSMAIIRYIADKHN<br>MLGGCPKERAEISMLEGAVLDIRYGVSRIAYSKDFETLKVDFLSKLPEMLKMFEDRLCHKTYLNGDHVTHPDFMLYDA<br>LDVVLYMDPMCLDAFPKLVCFKKRIEAIPQIDKYLKSSKYIAWPLQGWQATFGGGDHPPKIDTTENLYFQGAMGSMLV<br>LVLGDLHIPHRCNSLPAKFKKLLVPGKIQHILCTGNLCTKESYDYLKTLAGDVHIVRGDFDENLNYPEQKVVTVGQFKIG<br>LIHGHQVIPWGDMASLALLQRQFDVDILISGHTHKFEAFEHENKFYINPGSATGAYNALETNIIPSFVLMDIQASTVVTYV<br>YQLIGDDVKVERIEYKKP*
13	> His-Sumo3-SNX17<br>MGSSHHHHHHSSGLVPRGSHMNDHINLKVAGQDGSVVQFKIKRHTPLSKLMKAYCERQGLSMRQIRFRFDGQPINE<br>TDTPAQLEMEDEDTIDVFQQQTGGMHFSIPETESRSGDSGGSAYVAYNIHVNGVLHCRVRYSQLLGLHEQLRKEYGA<br>NVLPAFPPKKLFSLTPAEVEQRREQLEKYMQAVRQDPLLGSSETFNSFLRRAQQETQQVPTEEVSLEVLLSNGQKVL<br>VNVLTSDQTEDVLEAVAAKLDLPDDLIGYFSLFLVREKEDGAFSFVRKLQEFELPYVSVTSLRSQEYKIVLRKSYWDSA<br>YDDDVMENRVGLNLLYAQTVSDIERGWILVTKEQHRQLKSLQEKVSKKEFLRLAQTLRHYGYLRFDACVADFPEKDC<br>PVVVSAGNSELSLQLRLPGQQLREGSFRVTRMRCWRVTSSVPLPSGSTSSPGRGRGEVRLELAFEYLMSKDRLQW<br>VTITSPQAIMMSICLQSMVDELMVKKSGGSIRKMLRRRVGGTLRRSDSQQAVKSPPLLESPDATRESMVKLSSKLSAV<br>SLRGIGSPSTDASASDVHGNFAFEGIGDEDL
135	> His-Sumo3-SNX17$_{PX}$<br>MGSSHHHHHHSSGLVPRGSHMNDHINLKVAGQDGSVVQFKIKRHTPLSKLMKAYCERQGLSMRQIRFRFDGQPINE<br>TDTPAQLEMEDEDTIDVFQQQTGGMHFSIPETESRSGDSGGSAYVAYNIHVNGVLHCRVRYSQLLGLHEQLRKEYGA<br>NVLPAFPPKKLFSLTPAEVEQRREQLEKYMQAVRQDPLLGSSETFNSFLRRAQQE
1	> His-Sumo3-SNX17$_{109C}$<br>MGSSHHHHHHSSGLVPRGSHMNDHINLKVAGQDGSVVQFKIKRHTPLSKLMKAYCERQGLSMRQIRFRFDGQPINE<br>TDTPAQLEMEDEDTIDVFQQQTGGTQQVPTEEVSLEVLLSNGQKVLVNVLTSDQTEDVLEAVAAKLDLPDDLIGYFSL<br>FLVREKEDGAFSFVRKLQEFELPYVSVTSLRSQEYKIVLRKSYWDSAYDDDVMENRVGLNLLYAQTVSDIERGWILVT<br>KEQHRQLKSLQEKVSKKEFLRLAQTLRHYGYLRFDACVADFPEKDCPVVVSAGNSELSLQLRLPGQQLREGSFRVTR<br>MRCWRVTSSVPLPSGSTSSPGRGRGEVRLELAFEYLMSKDRLQWVTITSPQAIMMSICLQSMVDELMVKKSGGSIRK<br>MLRRRVGGTLRRSDSQQAVKSPPLLESPDATRESMVKLSSKLSAVSLRGIGSPSTDASASDVHGNFAFEGIGDEDL
14	> His-Sumo3-EGFP-SNX17<br>MGSSHHHHHHSSGLVPRGSHMNDHINLKVAGQDGSVVQFKIKRHTPLSKLMKAYCERQGLSMRQIRFRFDGQPINE<br>TDTPAQLEMEDEDTIDVFQQQTGGSVSKGEELFTGVVPILVELDGDVNGHKFSVSGEGEGDATYGKLTLKFICTTGKL<br>PVPWPTLVTTLTYGVQCFSRYPDHMKQHDFFKSAMPEGYVQERTIFFKDDGNYKTRAEVKFEGDTLVNRIELKGIDFK<br>EDGNILGHKLEYNYNSHNVYIMADKQKNGIKVNFKIRHNIEDGSVQLADHYQQNTPIGDGPVLLPDNHYLSTQSALSKD<br>PNEKRDHMVLLEFVTAAGITLGMDELYKGGGGSMHFSIPETESRSGDSGGSAYVAYNIHVNGVLHCRVRYSQLLGLH<br>EQLRKEYGANVLPAFPPKKLFSLTPAEVEQRREQLEKYMQAVRQDPLLGSSETFNSFLRRAQQETQQVPTEEVSLEV<br>LLSNGQKVLVNVLTSDQTEDVLEAVAAKLDLPDDLIGYFSLFLVREKEDGAFSFVRKLQEFELPYVSVTSLRSQEYKIVL<br>RKSYWDSAYDDDVMENRVGLNLLYAQTVSDIERGWILVTKEQHRQLKSLQEKVSKKEFLRLAQTLRHYGYLRFDACV<br>ADFPEKDCPVVVSAGNSELSLQLRLPGQQLREGSFRVTRMRCWRVTSSVPLPSGSTSSPGRGRGEVRLELAFEYLM<br>SKDRLQWVTITSPQAIMMSICLQSMVDELMVKKSGGSIRKMLRRRVGGTLRRSDSQQAVKSPPLLESPDATRESMVK<br>LSSKLSAVSLRGIGSPSTDASASDVHGNFAFEGIGDEDL
159	> His-TEV-MBP-SNX17<br>MGSSHHHHHHSSGENLYFQGMIEEGKLVIWINGDKGYNGLAEVGKKFEKDTGIKVTVEHPDKLEEKFPQVAATGDGP<br>DIIFWAHDRFGGYAQSGLLAEITPDKAFQDKLYPFTWDAVRYNGKLIAYPIAVEALSLIYNKDLLPNPPKTWEEIPALDK<br>ELKAKGKSALMFNLQEPYFTWPLIAADGGYAFKYENGKYDIKDVGVDNAGAKAGLTFLVDLIKNKHMNADTDYSIAEA<br>AFNKGETAMTINGPWAWSNIDTSKVNYGVTVLPTFKGQPSKPFVGVLSAGINAASPNKELAKEFLENYLLTDEGLEAV

	NKDKPLGAVALKSYEEELAKDPRIAATMENAQKGEIMPNIPQMSAFWYAVRTAVINAASGRQTVDEALKDAQTNSSS<br>MHFSIPETESRSGDSGGSAYVAYNIHVNGVLHCRVRYSQLLGLHEQLRKEYGANVLPAFPPKKLFSLTPAEVEQRRE<br>QLEKYMQAVRQDPLLGSSETFNSFLRRAQQETQQVPTEEVSLEVLLSNGQKVLVNVLTSDQTEDVLEAVAAKLDLPD<br>DLIGYFSLFLVREKEDGAFSFVRKLQEFELPYVSVTSLRSQEYKIVLRKSYWDSAYDDDVMENRVGLNLLYAQTVSDIE<br>RGWILVTKEQHRQLKSLQEKVSKKEFLRLAQTLRHYGYLRFDACVADFPEKDCPVVVSAGNSELSLQLRLPGQQLRE<br>GSFRVTRMRCWRVTSSVPLPSGSTSSPGRGRGEVRLELAFEYLMSKDRLQWVTITSPQAIMMSICLQSMVDELMVK<br>KSGGSIRKMLRRRVGGTLRRSDSQQAVKSPPLLESPDATRESMVKLSSKLSAVSLRGIGSPSTDASASDVHGNFAFE<br>GIGDEDL
**179**	> His-TEV-MBP-SNX17$_{D467X}$<br>MGSSHHHHHHSSGENLYFQGMIEEGKLVIWINGDKGYNGLAEVGKKFEKDTGIKVTVEHPDKLEEKFPQVAATGDGP<br>DIIFWAHDRFGGYAQSGLLAEITPDKAFQDKLYPFTWDAVRYNGKLIAYPIAVEALSLIYNKDLLPNPPKTWEEIPALDK<br>ELKAKGKSALMFNLQEPYFTWPLIAADGGYAFKYENGKYDIKDVGVDNAGAKAGLTFLVDLIKNKHMNADTDYSIAEA<br>AFNKGETAMTINGPWAWSNIDTSKVNYGVTVLPTFKGQPSKPFVGVLSAGINAASPNKELAKEFLENYLLTDEGLEAV<br>NKDKPLGAVALKSYEEELAKDPRIAATMENAQKGEIMPNIPQMSAFWYAVRTAVINAASGRQTVDEALKDAQTNSSS<br>MHFSIPETESRSGDSGGSAYVAYNIHVNGVLHCRVRYSQLLGLHEQLRKEYGANVLPAFPPKKLFSLTPAEVEQRRE<br>QLEKYMQAVRQDPLLGSSETFNSFLRRAQQETQQVPTEEVSLEVLLSNGQKVLVNVLTSDQTEDVLEAVAAKLDLPD<br>DLIGYFSLFLVREKEDGAFSFVRKLQEFELPYVSVTSLRSQEYKIVLRKSYWDSAYDDDVMENRVGLNLLYAQTVSDIE<br>RGWILVTKEQHRQLKSLQEKVSKKEFLRLAQTLRHYGYLRFDACVADFPEKDCPVVVSAGNSELSLQLRLPGQQLRE<br>GSFRVTRMRCWRVTSSVPLPSGSTSSPGRGRGEVRLELAFEYLMSKDRLQWVTITSPQAIMMSICLQSMVDELMVK<br>KSGGSIRKMLRRRVGGTLRRSDSQQAVKSPPLLESPDATRESMVKLSSKLSAVSLRGIGSPSTDASASDVHGNFAFE<br>GIG
**178**	> His-TEV-MBP-SNX17$_{L470G}$<br>MGSSHHHHHHSSGENLYFQGMIEEGKLVIWINGDKGYNGLAEVGKKFEKDTGIKVTVEHPDKLEEKFPQVAATGDGP<br>DIIFWAHDRFGGYAQSGLLAEITPDKAFQDKLYPFTWDAVRYNGKLIAYPIAVEALSLIYNKDLLPNPPKTWEEIPALDK<br>ELKAKGKSALMFNLQEPYFTWPLIAADGGYAFKYENGKYDIKDVGVDNAGAKAGLTFLVDLIKNKHMNADTDYSIAEA<br>AFNKGETAMTINGPWAWSNIDTSKVNYGVTVLPTFKGQPSKPFVGVLSAGINAASPNKELAKEFLENYLLTDEGLEAV<br>NKDKPLGAVALKSYEEELAKDPRIAATMENAQKGEIMPNIPQMSAFWYAVRTAVINAASGRQTVDEALKDAQTNSSS<br>MHFSIPETESRSGDSGGSAYVAYNIHVNGVLHCRVRYSQLLGLHEQLRKEYGANVLPAFPPKKLFSLTPAEVEQRRE<br>QLEKYMQAVRQDPLLGSSETFNSFLRRAQQETQQVPTEEVSLEVLLSNGQKVLVNVLTSDQTEDVLEAVAAKLDLPD<br>DLIGYFSLFLVREKEDGAFSFVRKLQEFELPYVSVTSLRSQEYKIVLRKSYWDSAYDDDVMENRVGLNLLYAQTVSDIE<br>RGWILVTKEQHRQLKSLQEKVSKKEFLRLAQTLRHYGYLRFDACVADFPEKDCPVVVSAGNSELSLQLRLPGQQLRE<br>GSFRVTRMRCWRVTSSVPLPSGSTSSPGRGRGEVRLELAFEYLMSKDRLQWVTITSPQAIMMSICLQSMVDELMVK<br>KSGGSIRKMLRRRVGGTLRRSDSQQAVKSPPLLESPDATRESMVKLSSKLSAVSLRGIGSPSTDASASDVHGNFAFE<br>GIGDEDG
**203**	> His-TEV-MBP-SNX17$_{W321A}$<br>MGSSHHHHHHSSGENLYFQGMIEEGKLVIWINGDKGYNGLAEVGKKFEKDTGIKVTVEHPDKLEEKFPQVAATGDGP<br>DIIFWAHDRFGGYAQSGLLAEITPDKAFQDKLYPFTWDAVRYNGKLIAYPIAVEALSLIYNKDLLPNPPKTWEEIPALDK<br>ELKAKGKSALMFNLQEPYFTWPLIAADGGYAFKYENGKYDIKDVGVDNAGAKAGLTFLVDLIKNKHMNADTDYSIAEA<br>AFNKGETAMTINGPWAWSNIDTSKVNYGVTVLPTFKGQPSKPFVGVLSAGINAASPNKELAKEFLENYLLTDEGLEAV<br>NKDKPLGAVALKSYEEELAKDPRIAATMENAQKGEIMPNIPQMSAFWYAVRTAVINAASGRQTVDEALKDAQTNSSS<br>MHFSIPETESRSGDSGGSAYVAYNIHVNGVLHCRVRYSQLLGLHEQLRKEYGANVLPAFPPKKLFSLTPAEVEQRRE<br>QLEKYMQAVRQDPLLGSSETFNSFLRRAQQETQQVPTEEVSLEVLLSNGQKVLVNVLTSDQTEDVLEAVAAKLDLPD<br>DLIGYFSLFLVREKEDGAFSFVRKLQEFELPYVSVTSLRSQEYKIVLRKSYWDSAYDDDVMENRVGLNLLYAQTVSDIE<br>RGWILVTKEQHRQLKSLQEKVSKKEFLRLAQTLRHYGYLRFDACVADFPEKDCPVVVSAGNSELSLQLRLPGQQLRE<br>GSFRVTRMRCARVTSSVPLPSGSTSSPGRGRGEVRLELAFEYLMSKDRLQWVTITSPQAIMMSICLQSMVDELMVKK<br>SGGSIRKMLRRRVGGTLRRSDSQQAVKSPPLLESPDATRESMVKLSSKLSAVSLRGIGSPSTDASASDVHGNFAFEGI<br>GDEDL
**204**	> His-TEV-MBP-SNX17$_{V380D}$<br>MGSSHHHHHHSSGENLYFQGMIEEGKLVIWINGDKGYNGLAEVGKKFEKDTGIKVTVEHPDKLEEKFPQVAATGDGP<br>DIIFWAHDRFGGYAQSGLLAEITPDKAFQDKLYPFTWDAVRYNGKLIAYPIAVEALSLIYNKDLLPNPPKTWEEIPALDK<br>ELKAKGKSALMFNLQEPYFTWPLIAADGGYAFKYENGKYDIKDVGVDNAGAKAGLTFLVDLIKNKHMNADTDYSIAEA<br>AFNKGETAMTINGPWAWSNIDTSKVNYGVTVLPTFKGQPSKPFVGVLSAGINAASPNKELAKEFLENYLLTDEGLEAV<br>NKDKPLGAVALKSYEEELAKDPRIAATMENAQKGEIMPNIPQMSAFWYAVRTAVINAASGRQTVDEALKDAQTNSSS<br>MHFSIPETESRSGDSGGSAYVAYNIHVNGVLHCRVRYSQLLGLHEQLRKEYGANVLPAFPPKKLFSLTPAEVEQRRE<br>QLEKYMQAVRQDPLLGSSETFNSFLRRAQQETQQVPTEEVSLEVLLSNGQKVLVNVLTSDQTEDVLEAVAAKLDLPD<br>DLIGYFSLFLVREKEDGAFSFVRKLQEFELPYVSVTSLRSQEYKIVLRKSYWDSAYDDDVMENRVGLNLLYAQTVSDIE<br>RGWILVTKEQHRQLKSLQEKVSKKEFLRLAQTLRHYGYLRFDACVADFPEKDCPVVVSAGNSELSLQLRLPGQQLRE<br>GSFRVTRMRCWRVTSSVPLPSGSTSSPGRGRGEVRLELAFEYLMSKDRLQWVTITSPQAIMMSICLQSMDDELMVK<br>KSGGSIRKMLRRRVGGTLRRSDSQQAVKSPPLLESPDATRESMVKLSSKLSAVSLRGIGSPSTDASASDVHGNFAFE<br>GIGDEDL
**205**	> His-TEV-MBP-SNX17$_{H457A}$<br>MGSSHHHHHHSSGENLYFQGMIEEGKLVIWINGDKGYNGLAEVGKKFEKDTGIKVTVEHPDKLEEKFPQVAATGDGP<br>DIIFWAHDRFGGYAQSGLLAEITPDKAFQDKLYPFTWDAVRYNGKLIAYPIAVEALSLIYNKDLLPNPPKTWEEIPALDK<br>ELKAKGKSALMFNLQEPYFTWPLIAADGGYAFKYENGKYDIKDVGVDNAGAKAGLTFLVDLIKNKHMNADTDYSIAEA<br>AFNKGETAMTINGPWAWSNIDTSKVNYGVTVLPTFKGQPSKPFVGVLSAGINAASPNKELAKEFLENYLLTDEGLEAV

	NKDKPLGAVALKSYEEELAKDPRIAATMENAQKGEIMPNIPQMSAFWYAVRTAVINAASGRQTVDEALKDAQTNSSS<br>MHFSIPETESRSGDSGGSAYVAYNIHVNGVLHCRVRYSQLLGLHEQLRKEYGANVLPAFPPKKLFSLTPAEVEQRRE<br>QLEKYMQAVRQDPLLGSSETFNSFLRRAQQETQQVPTEEVSLEVLLSNGQKVLVNVLTSDQTEDVLEAVAAKLDLPD<br>DLIGYFSLFLVREKEDGAFSFVRKLQEFELPYVSVTSLRSQEYKIVLRKSYWDSAYDDDVMENRVGLNLLYAQTVSDIE<br>RGWILVTKEQHRQLKSLQEKVSKKEFLRLAQTLRHYGYLRFDACVADFPEKDCPVVVSAGNSELSLQLRLPGQQLRE<br>GSFRVTRMRCWRVTSSVPLPSGSTSSPGRGRGEVRLELAFEYLMSKDRLQWVTITSPQAIMMSICLQSMVDELMVK<br>KSGGSIRKMLRRRVGGTLRRSDSQQAVKSPPLLESPDATRESMVKLSSKLSAVSLRGIGSPSTDASASDVAGNFAFE<br>GIGDEDL
**206**	> His-TEV-MBP-SNX17$_{N459A+F462A}$<br>MGSSHHHHHHSSGENLYFQGMIEEGKLVIWINGDKGYNGLAEVGKKFEKDTGIKVTVEHPDKLEEKFPQVAATGDGP<br>DIIFWAHDRFGGYAQSGLLAEITPDKAFQDKLYPFTWDAVRYNGKLIAYPIAVEALSLIYNKDLLPNPPKTWEEIPALDK<br>ELKAKGKSALMFNLQEPYFTWPLIAADGGYAFKYENGKYDIKDVGVDNAGAKAGLTFLVDLIKNKHMNADTDYSIAEA<br>AFNKGETAMTINGPWAWSNIDTSKVNYGVTVLPTFKGQPSKPFVGVLSAGINAASPNKELAKEFLENYLLTDEGLEAV<br>NKDKPLGAVALKSYEEELAKDPRIAATMENAQKGEIMPNIPQMSAFWYAVRTAVINAASGRQTVDEALKDAQTNSSS<br>MHFSIPETESRSGDSGGSAYVAYNIHVNGVLHCRVRYSQLLGLHEQLRKEYGANVLPAFPPKKLFSLTPAEVEQRRE<br>QLEKYMQAVRQDPLLGSSETFNSFLRRAQQETQQVPTEEVSLEVLLSNGQKVLVNVLTSDQTEDVLEAVAAKLDLPD<br>DLIGYFSLFLVREKEDGAFSFVRKLQEFELPYVSVTSLRSQEYKIVLRKSYWDSAYDDDVMENRVGLNLLYAQTVSDIE<br>RGWILVTKEQHRQLKSLQEKVSKKEFLRLAQTLRHYGYLRFDACVADFPEKDCPVVVSAGNSELSLQLRLPGQQLRE<br>GSFRVTRMRCWRVTSSVPLPSGSTSSPGRGRGEVRLELAFEYLMSKDRLQWVTITSPQAIMMSICLQSMVDELMVK<br>KSGGSIRKMLRRRVGGTLRRSDSQQAVKSPPLLESPDATRESMVKLSSKLSAVSLRGIGSPSTDASASDVHGAFAAE<br>GIGDEDL
**18**	> GST-TEV-LRP1$_{ICD}$<br>MGSSMSPILGYWKIKGLVQPTRLLLEYLEEKYEEHLYERDEGDKWRNKKFELGLEFPNLPYYIDGDVKLTQSMAIIRYI<br>ADKHNMLGGCPKERAEISMLEGAVLDIRYGVSRIAYSKDFETLKVDFLSKLPEMLKMFEDRLCHKTYLNGDHVTHPDF<br>MLYDALDVVLYMDPMCLDAFPKLVCFKKRIEAIPQIDKYLKSSKYIAWPLQGWQATFGGGDHPPKSSGENLYFQGYK<br>RRVQGAKGFQHQRMTNGAMNVEIGNPTYKMYEGGEPDDVGGLLDADFALDPDKPTNFTNPVYATLYMGGHGSRHS<br>LASTDEKRELLGRGPEDEIGDPLA
**275**	>GST-TEV-LRP1$_{ICD-mut}$ (N4470A+Y4473A)<br>MGSSMSPILGYWKIKGLVQPTRLLLEYLEEKYEEHLYERDEGDKWRNKKFELGLEFPNLPYYIDGDVKLTQSMAIIRYI<br>ADKHNMLGGCPKERAEISMLEGAVLDIRYGVSRIAYSKDFETLKVDFLSKLPEMLKMFEDRLCHKTYLNGDHVTHPDF<br>MLYDALDVVLYMDPMCLDAFPKLVCFKKRIEAIPQIDKYLKSSKYIAWPLQGWQATFGGGDHPPKSSGENLYFQGYK<br>RRVQGAKGFQHQRMTNGAMNVEIGAPTAKMYEGGEPDDVGGLLDADFALDPDKPTNFTNPVYATLYMGGHGSRHS<br>LASTDEKRELLGRGPEDEIGDPLA
**173**	> MBP-TEV-His$_{10}$-LRP1$_{ICD}$<br>MSYYMIEEGKLVIWINGDKGYNGLAEVGKKFEKDTGIKVTVEHPDKLEEKFPQVAATGDGPDIIFWAHDRFGGYAQSG<br>LLAEITPDKAFQDKLYPFTWDAVRYNGKLIAYPIAVEALSLIYNKDLLPNPPKTWEEIPALDKELKAKGKSALMFNLQEP<br>YFTWPLIAADGGYAFKYENGKYDIKDVGVDNAGAKAGLTFLVDLIKNKHMNADTDYSIAEAAFNKGETAMTINGPWAW<br>SNIDTSKVNYGVTVLPTFKGQPSKPFVGVLSAGINAASPNKELAKEFLENYLLTDEGLEAVNKDKPLGAVALKSYEEEL<br>AKDPRIAATMENAQKGEIMPNIPQMSAFWYAVRTAVINAASGRQTVDEALKDAQTENLYFQGHHHHHHHHHHYKRR<br>VQGAKGFQHQRMTNGAMNVEIGNPTYKMYEGGEPDDVGGLLDADFALDPDKPTNFTNPVYATLYMGGHGSRHSLA<br>STDEKRELLGRGPEDEIGDPLA
**49**	> GST-TEV-APP$_{ICD}$<br>MGSSMSPILGYWKIKGLVQPTRLLLEYLEEKYEEHLYERDEGDKWRNKKFELGLEFPNLPYYIDGDVKLTQSMAIIRYI<br>ADKHNMLGGCPKERAEISMLEGAVLDIRYGVSRIAYSKDFETLKVDFLSKLPEMLKMFEDRLCHKTYLNGDHVTHPDF<br>MLYDALDVVLYMDPMCLDAFPKLVCFKKRIEAIPQIDKYLKSSKYIAWPLQGWQATFGGGDHPPKSSGENLYFQGKKK<br>QYTSIHHGVVEVDAAVTPEERHLSKMQQNGYENPTYKFFEQMQN
**168**	> GST-TEV-ITGB1$_{ICD}$<br>MGSSMSPILGYWKIKGLVQPTRLLLEYLEEKYEEHLYERDEGDKWRNKKFELGLEFPNLPYYIDGDVKLTQSMAIIRYI<br>ADKHNMLGGCPKERAEISMLEGAVLDIRYGVSRIAYSKDFETLKVDFLSKLPEMLKMFEDRLCHKTYLNGDHVTHPDF<br>MLYDALDVVLYMDPMCLDAFPKLVCFKKRIEAIPQIDKYLKSSKYIAWPLQGWQATFGGGDHPPKSSGENLYFQGKLL<br>MIIHDRREFAKFEKEKMNAKWDTGENPIYKSAVTTVVNPKYEGK
**28**	> GST-TEV-L2$_{FBR}$<br>MGSSMSPILGYWKIKGLVQPTRLLLEYLEEKYEEHLYERDEGDKWRNKKFELGLEFPNLPYYIDGDVKLTQSMAIIRYI<br>ADKHNMLGGCPKERAEISMLEGAVLDIRYGVSRIAYSKDFETLKVDFLSKLPEMLKMFEDRLCHKTYLNGDHVTHPDF<br>MLYDALDVVLYMDPMCLDAFPKLVCFKKRIEAIPQIDKYLKSSKYIAWPLQGWQATFGGGDHPPKSSGENLYFQGDP<br>AFVTTPTKLITYDNPAYEGIDVDNTLYF
**276**	>GST-TEV-L2$_{FBR-mut}$ (N254A+Y257A)<br>MGSSMSPILGYWKIKGLVQPTRLLLEYLEEKYEEHLYERDEGDKWRNKKFELGLEFPNLPYYIDGDVKLTQSMAIIRYI<br>ADKHNMLGGCPKERAEISMLEGAVLDIRYGVSRIAYSKDFETLKVDFLSKLPEMLKMFEDRLCHKTYLNGDHVTHPDF<br>MLYDALDVVLYMDPMCLDAFPKLVCFKKRIEAIPQIDKYLKSSKYIAWPLQGWQATFGGGDHPPKSSGENLYFQGDP<br>AFVTTPTKLITYDAPAAEGIDVDNTLYF
**131**	> GST-HRV3C-His$_{10}$-L2$_{FBR}$<br>MSPILGYWKIKGLVQPTRLLLEYLEEKYEEHLYERDEGDKWRNKKFELGLEFPNLPYYIDGDVKLTQSMAIIRYIADKHN<br>MLGGCPKERAEISMLEGAVLDIRYGVSRIAYSKDFETLKVDFLSKLPEMLKMFEDRLCHKTYLNGDHVTHPDFMLYDA

	LDVVLYMDPMCLDAFPKLVCFKKRIEAIPQIDKYLKSSKYIAWPLQGWQATFGGGDHPPKSSGLEVLFQGPHHHHHH HHHHDPAFVTTPTKLITYDNPAYEGIDVDNTLYF
**85**	> His-TEV-VPS35L-TwinStrep-TEV-VPS26C-VPS29<br>MGSSHHHHHHSSGENLYFQGMAVFPWHSRNRNYKAEFASCRLEAVPLEFGDYHPLKPITVTESKTKKVNRKGSTSS TSSSSSSSVVDPLSSVLDGTDPLSMFAATADPAALAAAMDSSRRKRDRDDNSVVGSDFEPWTNKRGEILARYTTTEK LSINLFMGSEKGKAGTATLAMSEKVRTRLEELDDFEEGSQKELLNLTQQDYVNRIEELNQSLKDAWASDQKVKALKIVI QCSKLLSDTSVIQFYPSKFVLITDILDTFGKLVYERIFSMCVDSRSVLPDHFSPENANDTAKETCLNWFFKIASIRELIPRF YVEASILKCNKFLSKTGISECLPRLTCMIRGIGDPLVSVYARAYLCRVGMEVAPHLKETLNKNFFDFLLTFKQIHGDTVQ NQLVVQGVELPSYLPLYPPAMDWIFQCISYHAPEALLTEMMERCKKLGNNALLLNSVMSAFRAEFIATRSMDFIGMIKE CDESGFPKHLLFRSLGLNLALADPPESDRLQILNEAWKVITKLKNPQDYINCAEVWVEYTCKHFTKREVNTVLADVIKH MTPDRAFEDSYPQLQLIIKKVIAHFHDFSVLFSVEKFLPFLDMFQKESVRVEVCKCIMDAFIKHQQEPTKDPVILNALLH VCKTMHDSVNALTLEDEKRMLSYLINGFIKMVSFGRDFEQQLSFYVESRSMFCNLEPVLVQLIHSVNRLAMETRKVMK GNHSRKTAAFVRACVAYCFITIPSLAGIFTRLNLYLHSGQVALANQCLSQADAFFKAAISLVPEVPKMINIDGKMRPSES FLLEFLCNFFSTLLIVPDHPEHGVLFLVRELLNVIQDYTWEDNSDEKIRIYTCVLHLLSAMSQETYLYHIDKVDSNDSLYG GDSKFLAENNKLCETVMAQILEHLKTLAKDEALKRQSSLGLSFFNSILAHGDLRNNKLNQLSVNLWHLAQRHGCADTR TMVKTLEYIKKQSKQPDMTHLTELALRLPLQTRT<br>MASWSHPQFEKGGGSGGGSGGSAWSHPQFEKSSGENLYFQGMGTALDIKIKRANKVYHAGEVLSGVVVISSKDSV QHQGVSLTMEGTVNLQLSAKSVGVFEAFYNSVKPIQIINSTIEMVKPGKFPSGKTEIPFEFPLHLKGNKVLYETYHGVFV NIQYTLRCDMKRSLLAKDLTKTCEFIVHSAPQKGKFTPSPVDFTITPETLQNVKERALLPKFLLRGHLNSTNCVITQPLT GELVVESSEAAIRSVELQLVRVETCGCAEGYARDATEIQNIQIADGDVCRGLSVPIYMVFPRLFTCPTLETTNFKVEFEV NIVVLLHPDHLITENFPLKLCRI<br>MLVLVLGDLHIPHRCNSLPAKFKKLLVPGKIQHILCTGNLCTKESYDYLKTLAGDVHIVRGDFDENLNYPEQKVVTVGQ FKIGLIHGHQVIPWGDMASLALLQRQFDVDILISGHTHKFEAFEHENKFYINPGSATGAYNALETNIIPSFVLMDIQASTV VTYVYQLIGDDVKVERIEYKKP
**122**	> His-TEV-VPS35L-TwinStrep-TEV-VPS26C-GST-HRV3C-VPS29<br>MGSSHHHHHHSSGENLYFQGMAVFPWHSRNRNYKAEFASCRLEAVPLEFGDYHPLKPITVTESKTKKVNRKGSTSS TSSSSSSSVVDPLSSVLDGTDPLSMFAATADPAALAAAMDSSRRKRDRDDNSVVGSDFEPWTNKRGEILARYTTTEK LSINLFMGSEKGKAGTATLAMSEKVRTRLEELDDFEEGSQKELLNLTQQDYVNRIEELNQSLKDAWASDQKVKALKIVI QCSKLLSDTSVIQFYPSKFVLITDILDTFGKLVYERIFSMCVDSRSVLPDHFSPENANDTAKETCLNWFFKIASIRELIPRF YVEASILKCNKFLSKTGISECLPRLTCMIRGIGDPLVSVYARAYLCRVGMEVAPHLKETLNKNFFDFLLTFKQIHGDTVQ NQLVVQGVELPSYLPLYPPAMDWIFQCISYHAPEALLTEMMERCKKLGNNALLLNSVMSAFRAEFIATRSMDFIGMIKE CDESGFPKHLLFRSLGLNLALADPPESDRLQILNEAWKVITKLKNPQDYINCAEVWVEYTCKHFTKREVNTVLADVIKH MTPDRAFEDSYPQLQLIIKKVIAHFHDFSVLFSVEKFLPFLDMFQKESVRVEVCKCIMDAFIKHQQEPTKDPVILNALLH VCKTMHDSVNALTLEDEKRMLSYLINGFIKMVSFGRDFEQQLSFYVESRSMFCNLEPVLVQLIHSVNRLAMETRKVMK GNHSRKTAAFVRACVAYCFITIPSLAGIFTRLNLYLHSGQVALANQCLSQADAFFKAAISLVPEVPKMINIDGKMRPSES FLLEFLCNFFSTLLIVPDHPEHGVLFLVRELLNVIQDYTWEDNSDEKIRIYTCVLHLLSAMSQETYLYHIDKVDSNDSLYG GDSKFLAENNKLCETVMAQILEHLKTLAKDEALKRQSSLGLSFFNSILAHGDLRNNKLNQLSVNLWHLAQRHGCADTR TMVKTLEYIKKQSKQPDMTHLTELALRLPLQTRT<br>MASWSHPQFEKGGGSGGGSGGSAWSHPQFEKSSGENLYFQGMGTALDIKIKRANKVYHAGEVLSGVVVISSKDSV QHQGVSLTMEGTVNLQLSAKSVGVFEAFYNSVKPIQIINSTIEMVKPGKFPSGKTEIPFEFPLHLKGNKVLYETYHGVFV NIQYTLRCDMKRSLLAKDLTKTCEFIVHSAPQKGKFTPSPVDFTITPETLQNVKERALLPKFLLRGHLNSTNCVITQPLT GELVVESSEAAIRSVELQLVRVETCGCAEGYARDATEIQNIQIADGDVCRGLSVPIYMVFPRLFTCPTLETTNFKVEFEV NIVVLLHPDHLITENFPLKLCRI<br>MSPILGYWKIKGLVQPTRLLLEYLEEKYEEHLYERDEGDKWRNKKFELGLEFPNLPYYIDGDVKLTQSMAIIRYIADKHN MLGGCPKERAEISMLEGAVLDIRYGVSRIAYSKDFETLKVDFLSKLPEMLKMFEDRLCHKTYLNGDHVTHPDFMLYDA LDVVLYMDPMCLDAFPKLVCFKKRIEAIPQIDKYLKSSKYIAWPLQGWQATFGGGDHPPKSDLEVLFQGPMLVLVLGD LHIPHRCNSLPAKFKKLLVPGKIQHILCTGNLCTKESYDYLKTLAGDVHIVRGDFDENLNYPEQKVVTVGQFKIGLIHGH QVIPWGDMASLALLQRQFDVDILISGHTHKFEAFEHENKFYINPGSATGAYNALETNIIPSFVLMDIQASTVVTYVYQLIG DDVKVERIEYKKP
**118**	> His-TEV-VPS35L$_{110\text{-}963}$-TwinStrep-TEV-VPS26C-VPS29<br>MGSSHHHHHHSSGENLYFQGVGSDFEPWTNKRGEILARYTTTEKLSINLFMGSEKGKAGTATLAMSEKVRTRLEELD DFEEGSQKELLNLTQQDYVNRIEELNQSLKDAWASDQKVKALKIVIQCSKLLSDTSVIQFYPSKFVLITDILDTFGKLVYE RIFSMCVDSRSVLPDHFSPENANDTAKETCLNWFFKIASIRELIPRFYVEASILKCNKFLSKTGISECLPRLTCMIRGIGD PLVSVYARAYLCRVGMEVAPHLKETLNKNFFDFLLTFKQIHGDTVQNQLVVQGVELPSYLPLYPPAMDWIFQCISYHA PEALLTEMMERCKKLGNNALLLNSVMSAFRAEFIATRSMDFIGMIKECDESGFPKHLLFRSLGLNLALADPPESDRLQI LNEAWKVITKLKNPQDYINCAEVWVEYTCKHFTKREVNTVLADVIKHMTPDRAFEDSYPQLQLIIKKVIAHFHDFSVLFS VEKFLPFLDMFQKESVRVEVCKCIMDAFIKHQQEPTKDPVILNALLHVCKTMHDSVNALTLEDEKRMLSYLINGFIKMV SFGRDFEQQLSFYVESRSMFCNLEPVLVQLIHSVNRLAMETRKVMKGNHSRKTAAFVRACVAYCFITIPSLAGIFTRLN LYLHSGQVALANQCLSQADAFFKAAISLVPEVPKMINIDGKMRPSESFLLEFLCNFFSTLLIVPDHPEHGVLFLVRELLN VIQDYTWEDNSDEKIRIYTCVLHLLSAMSQETYLYHIDKVDSNDSLYGGDSKFLAENNKLCETVMAQILEHLKTLAKDE ALKRQSSLGLSFFNSILAHGDLRNNKLNQLSVNLWHLAQRHGCADTRTMVKTLEYIKKQSKQPDMTHLTELALRLPLQ TRT<br>MASWSHPQFEKGGGSGGGSGGSAWSHPQFEKSSGENLYFQGMGTALDIKIKRANKVYHAGEVLSGVVVISSKDSV QHQGVSLTMEGTVNLQLSAKSVGVFEAFYNSVKPIQIINSTIEMVKPGKFPSGKTEIPFEFPLHLKGNKVLYETYHGVFV NIQYTLRCDMKRSLLAKDLTKTCEFIVHSAPQKGKFTPSPVDFTITPETLQNVKERALLPKFLLRGHLNSTNCVITQPLT

	GELVVESSEAAIRSVELQLVRVETCGCAEGYARDATEIQNIQIADGDVCRGLSVPIYMVFPRLFTCPTLETTNFKVEFEV<br>NIVVLLHPDHLITENFPLKLCRI<br>MLVLVLGDLHIPHRCNSLPAKFKKLLVPGKIQHILCTGNLCTKESYDYLKTLAGDVHIVRGDFDENLNYPEQKVVTVGQ<br>FKIGLIHGHQVIPWGDMASLALLQRQFDVDILISGHTHKFEAFEHENKFYINPGSATGAYNALETNIIPSFVLMDIQASTV<br>VTYVYQLIGDDVKVERIEYKKP
**116**	> His-TEV-VPS35L$_{110-598}$-TwinStrep-TEV-VPS26C-VPS29<br>MGSSHHHHHHSSGENLYFQGVGSDFEPWTNKRGEILARYTTTEKLSINLFMGSEKGKAGTATLAMSEKVRTRLEELD<br>DFEEGSQKELLNLTQQDYVNRIEELNQSLKDAWASDQKVKALKIVIQCSKLLSDTSVIQFYPSKFVLITDILDTFGKLVYE<br>RIFSMCVDSRSVLPDHFSPENANDTAKETCLNWFFKIASIRELIPRFYVEASILKCNKFLSKTGISECLPRLTCMIRGIGD<br>PLVSVYARAYLCRVGMEVAPHLKETLNKNFFDFLLTFKQIHGDTVQNQLVVQGVELPSYLPLYPPAMDWIFQCISYHA<br>PEALLTEMMERCKKLGNNALLLNSVMSAFRAEFIATRSMDFIGMIKECDESGFPKHLLFRSLGLNLALADPPESDRLQI<br>LNEAWKVITKLKNPQDYINCAEVWVEYTCKHFTKREVNTVLADVIKHMTPDRAFEDSYPQLQLIIKKVIAHFHDFSVLFS<br>VEKFLPFLDMFQKESVRVEVCKCIMDAFIKHQQ<br>MASWSHPQFEKGGGSGGGSGGSAWSHPQFEKSSGENLYFQGMGTALDIKIKRANKVYHAGEVLSGVVISSKDSV<br>QHQGVSLTMEGTVNLQLSAKSVGVFEAFYNSVKPIQIINSTIEMVKPGKFPSGKTEIPFEFPLHLKGNKVLYETYHGVFV<br>NIQYTLRCDMKRSLLAKDLTKTCEFIVHSAPQKGKFTPSPVDFTITPETLQNVKERALLPKFLLRGHLNSTNCVITQPLT<br>GELVVESSEAAIRSVELQLVRVETCGCAEGYARDATEIQNIQIADGDVCRGLSVPIYMVFPRLFTCPTLETTNFKVEFEV<br>NIVVLLHPDHLITENFPLKLCRI<br>MLVLVLGDLHIPHRCNSLPAKFKKLLVPGKIQHILCTGNLCTKESYDYLKTLAGDVHIVRGDFDENLNYPEQKVVTVGQ<br>FKIGLIHGHQVIPWGDMASLALLQRQFDVDILISGHTHKFEAFEHENKFYINPGSATGAYNALETNIIPSFVLMDIQASTV<br>VTYVYQLIGDDVKVERIEYKKP
**138**	> His-TEV-VPS35L$_{1-598}$-TwinStrep-TEV-VPS26C-VPS29<br>MGSSHHHHHHSSGENLYFQGMAVFPWHSRNRNYKAEFASCRLEAVPLEFGDYHPLKPITVTESKTKKVNRKGSTSS<br>TSSSSSSSVVDPLSSVLDGTDPLSMFAATADPAALAAAMDSSRRKRDRDDNSVVGSDFEPWTNKRGEILARYTTTEK<br>LSINLFMGSEKGKAGTATLAMSEKVRTRLEELDDFEEGSQKELLNLTQQDYVNRIEELNQSLKDAWASDQKVKALKIVI<br>QCSKLLSDTSVIQFYPSKFVLITDILDTFGKLVYERIFSMCVDSRSVLPDHFSPENANDTAKETCLNWFFKIASIRELIPRF<br>YVEASILKCNKFLSKTGISECLPRLTCMIRGIGDPLVSVYARAYLCRVGMEVAPHLKETLNKNFFDFLLTFKQIHGDTVQ<br>NQLVVQGVELPSYLPLYPPAMDWIFQCISYHAPEALLTEMMERCKKLGNNALLLNSVMSAFRAEFIATRSMDFIGMIKE<br>CDESGFPKHLLFRSLGLNLALADPPESDRLQILNEAWKVITKLKNPQDYINCAEVWVEYTCKHFTKREVNTVLADVIKH<br>MTPDRAFEDSYPQLQLIIKKVIAHFHDFSVLFSVEKFLPFLDMFQKESVRVEVCKCIMDAFIKHQQ<br>MASWSHPQFEKGGGSGGGSGGSAWSHPQFEKSSGENLYFQGMGTALDIKIKRANKVYHAGEVLSGVVISSKDSV<br>QHQGVSLTMEGTVNLQLSAKSVGVFEAFYNSVKPIQIINSTIEMVKPGKFPSGKTEIPFEFPLHLKGNKVLYETYHGVFV<br>NIQYTLRCDMKRSLLAKDLTKTCEFIVHSAPQKGKFTPSPVDFTITPETLQNVKERALLPKFLLRGHLNSTNCVITQPLT<br>GELVVESSEAAIRSVELQLVRVETCGCAEGYARDATEIQNIQIADGDVCRGLSVPIYMVFPRLFTCPTLETTNFKVEFEV<br>NIVVLLHPDHLITENFPLKLCRI<br>MLVLVLGDLHIPHRCNSLPAKFKKLLVPGKIQHILCTGNLCTKESYDYLKTLAGDVHIVRGDFDENLNYPEQKVVTVGQ<br>FKIGLIHGHQVIPWGDMASLALLQRQFDVDILISGHTHKFEAFEHENKFYINPGSATGAYNALETNIIPSFVLMDIQASTV<br>VTYVYQLIGDDVKVERIEYKKP
**144**	> His-TEV-VPS35L$_{1-436}$-TwinStrep-TEV-VPS26C-VPS29<br>MGSSHHHHHHSSGENLYFQGMAVFPWHSRNRNYKAEFASCRLEAVPLEFGDYHPLKPITVTESKTKKVNRKGSTSS<br>TSSSSSSSVVDPLSSVLDGTDPLSMFAATADPAALAAAMDSSRRKRDRDDNSVVGSDFEPWTNKRGEILARYTTTEK<br>LSINLFMGSEKGKAGTATLAMSEKVRTRLEELDDFEEGSQKELLNLTQQDYVNRIEELNQSLKDAWASDQKVKALKIVI<br>QCSKLLSDTSVIQFYPSKFVLITDILDTFGKLVYERIFSMCVDSRSVLPDHFSPENANDTAKETCLNWFFKIASIRELIPRF<br>YVEASILKCNKFLSKTGISECLPRLTCMIRGIGDPLVSVYARAYLCRVGMEVAPHLKETLNKNFFDFLLTFKQIHGDTVQ<br>NQLVVQGVELPSYLPLYPPAMDWIFQCISYHAPEALLTEMMERCKKLGNNALLLNSVMSAFR<br>MASWSHPQFEKGGGSGGGSGGSAWSHPQFEKSSGENLYFQGMGTALDIKIKRANKVYHAGEVLSGVVISSKDSV<br>QHQGVSLTMEGTVNLQLSAKSVGVFEAFYNSVKPIQIINSTIEMVKPGKFPSGKTEIPFEFPLHLKGNKVLYETYHGVFV<br>NIQYTLRCDMKRSLLAKDLTKTCEFIVHSAPQKGKFTPSPVDFTITPETLQNVKERALLPKFLLRGHLNSTNCVITQPLT<br>GELVVESSEAAIRSVELQLVRVETCGCAEGYARDATEIQNIQIADGDVCRGLSVPIYMVFPRLFTCPTLETTNFKVEFEV<br>NIVVLLHPDHLITENFPLKLCRI<br>MLVLVLGDLHIPHRCNSLPAKFKKLLVPGKIQHILCTGNLCTKESYDYLKTLAGDVHIVRGDFDENLNYPEQKVVTVGQ<br>FKIGLIHGHQVIPWGDMASLALLQRQFDVDILISGHTHKFEAFEHENKFYINPGSATGAYNALETNIIPSFVLMDIQASTV<br>VTYVYQLIGDDVKVERIEYKKP
**151**	> His-TEV-VPS35L-GST-HRV3C-VPS29<br>MGSSHHHHHHSSGENLYFQGMAVFPWHSRNRNYKAEFASCRLEAVPLEFGDYHPLKPITVTESKTKKVNRKGSTSS<br>TSSSSSSSVVDPLSSVLDGTDPLSMFAATADPAALAAAMDSSRRKRDRDDNSVVGSDFEPWTNKRGEILARYTTTEK<br>LSINLFMGSEKGKAGTATLAMSEKVRTRLEELDDFEEGSQKELLNLTQQDYVNRIEELNQSLKDAWASDQKVKALKIVI<br>QCSKLLSDTSVIQFYPSKFVLITDILDTFGKLVYERIFSMCVDSRSVLPDHFSPENANDTAKETCLNWFFKIASIRELIPRF<br>YVEASILKCNKFLSKTGISECLPRLTCMIRGIGDPLVSVYARAYLCRVGMEVAPHLKETLNKNFFDFLLTFKQIHGDTVQ<br>NQLVVQGVELPSYLPLYPPAMDWIFQCISYHAPEALLTEMMERCKKLGNNALLLNSVMSAFRAEFIATRSMDFIGMIKE<br>CDESGFPKHLLFRSLGLNLALADPPESDRLQILNEAWKVITKLKNPQDYINCAEVWVEYTCKHFTKREVNTVLADVIKH<br>MTPDRAFEDSYPQLQLIIKKVIAHFHDFSVLFSVEKFLPFLDMFQKESVRVEVCKCIMDAFIKHQQEPTKDPVILNALLH<br>VCKTMHDSVNALTLEDEKRMLSYLINGFIKMVSFGRDFEQQLSFYVESRSMFCNLEPVLVQLIHSVNRLAMETRKVMK<br>GNHSRKTAAFVRACVAYCFITIPSLAGIFTRLNLYLHSGQVALANQCLSQADAFFKAAISLVPEVPKMINIDGKMRPSES<br>FLLEFLCNFFSTLLIVPDHPEHGVLFLVRELLNVIQDYTWEDNSDEKIRIYTCVLHLLSAMSQETYLYHIDKVDSNDSLYG

	GDSKFLAENNKLCETVMAQILEHLKTLAKDEALKRQSSLGLSFFNSILAHGDLRNNKLNQLSVNLWHLAQRHGCADTR<br>TMVKTLEYIKKQSKQPDMTHLTELALRLPLQTRT<br>MSPILGYWKIKGLVQPTRLLLEYLEEKYEEHLYERDEGDKWRNKKFELGLEFPNLPYYIDGDVKLTQSMAIIRYIADKHN<br>MLGGCPKERAEISMLEGAVLDIRYGVSRIAYSKDFETLKVDFLSKLPEMLKMFEDRLCHKTYLNGDHVTHPDFMLYDA<br>LDVVLYMDPMCLDAFPKLVCFKKRIEAIPQIDKYLKSSKYIAWPLQGWQATFGGGDHPPKSDLEVLFQGPMLVLVLGD<br>LHIPHRCNSLPAKFKKLLVPGKIQHILCTGNLCTKESYDYLKTLAGDVHIVRGDFDENLNYPEQKVVTVGQFKIGLIHGH<br>QVIPWGDMASLALLQRQFDVDILISGHTHKFEAFEHENKFYINPGSATGAYNALETNIIPSFVLMDIQASTVVTYVYQLIG<br>DDVKVERIEYKKP
**231**	> His-TEV-VPS35L$_{R248E+W280D}$-TwinStrep-TEV-VPS26C-VPS29<br>MGSSHHHHHHSSGENLYFQGMAVFPWHSRNRNYKAEFASCRLEAVPLEFGDYHPLKPITVTESKTKKVNRKGSTSS<br>TSSSSSSSVVDPLSSVLDGTDPLSMFAATADPAALAAAMDSSRRKRDRDDNSVVGSDFEPWTNKRGEILARYTTTEK<br>LSINLFMGSEKGKAGTATLAMSEKVRTRLEELDDFEEGSQKELLNLTQQDYVNRIEELNQSLKDAWASDQKVKALKIVI<br>QCSKLLSDTSVIQFYPSKFVLITDILDTFGKLVYEEIFSMCVDSRSVLPDHFSPENANDTAKETCLNDFFKIASIRELIPRF<br>YVEASILKCNKFLSKTGISECLPRLTCMIRGIGDPLVSVYARAYLCRVGMEVAPHLKETLNKNFFDFLLTFKQIHGDTVQ<br>NQLVVQGVELPSYLPLYPPAMDWIFQCISYHAPEALLTEMMERCKKLGNNALLLNSVMSAFRAEFIATRSMDFIGMIKE<br>CDESGFPKHLLFRSLGLNLALADPPESDRLQILNEAWKVITKLKNPQDYINCAEVWVEYTCKHFTKREVNTVLADVIKH<br>MTPDRAFEDSYPQLQLIIKKVIAHFHDFSVLFSVEKFLPFLDMFQKESVRVEVCKCIMDAFIKHQQEPTKDPVILNALLH<br>VCKTMHDSVNALTLEDEKRMLSYLINGFIKMVSFGRDFEQQLSFYVESRSMFCNLEPVLVQLIHSVNRLAMETRKVMK<br>GNHSRKTAAFVRACVAYCFITIPSLAGIFTRLNLYLHSGQVALANQCLSQADAFFKAAISLVPEVPKMINIDGKMRPSES<br>FLLEFLCNFFSTLLIVPDHPEHGVLFLVRELLNVIQDYTWEDNSDEKIRIYTCVLHLLSAMSQETYLYHIDKVDSNDSLYG<br>GDSKFLAENNKLCETVMAQILEHLKTLAKDEALKRQSSLGLSFFNSILAHGDLRNNKLNQLSVNLWHLAQRHGCADTR<br>TMVKTLEYIKKQSKQPDMTHLTELALRLPLQTRT<br>MASWSHPQFEKGGGSGGGSGGSAWSHPQFEKSSGENLYFQGMGTALDIKIKRANKVYHAGEVLSGVVVISSKDSV<br>QHQGVSLTMEGTVNLQLSAKSVGVFEAFYNSVKPIQIINSTIEMVKPGKFPSGKTEIPFEFPLHLKGNKVLYETYHGVFV<br>NIQYTLRCDMKRSLLAKDLTKTCEFIVHSAPQKGKFTPSPVDFTITPETLQNVKERALLPKFLLRGHLNSTNCVITQPLT<br>GELVVESSEAAIRSVELQLVRVETCGCAEGYARDATEIQNIQIADGDVCRGLSVPIYMVFPRLFTCPTLETTNFKVEFEV<br>NIVVLLHPDHLITENFPLKLCRI<br>MLVLVLGDLHIPHRCNSLPAKFKKLLVPGKIQHILCTGNLCTKESYDYLKTLAGDVHIVRGDFDENLNYPEQKVVTVGQ<br>FKIGLIHGHQVIPWGDMASLALLQRQFDVDILISGHTHKFEAFEHENKFYINPGSATGAYNALETNIIPSFVLMDIQASTV<br>VTYVYQLIGDDVKVERIEYKKP
**232**	> His-TEV-VPS35L$_{K157E+R161E}$-TwinStrep-TEV-VPS26C-VPS29<br>MGSSHHHHHHSSGENLYFQGMAVFPWHSRNRNYKAEFASCRLEAVPLEFGDYHPLKPITVTESKTKKVNRKGSTSS<br>TSSSSSSSVVDPLSSVLDGTDPLSMFAATADPAALAAAMDSSRRKRDRDDNSVVGSDFEPWTNKRGEILARYTTTEK<br>LSINLFMGSEKGKAGTATLAMSEEVRTELEELDDFEEGSQKELLNLTQQDYVNRIEELNQSLKDAWASDQKVKALKIVI<br>QCSKLLSDTSVIQFYPSKFVLITDILDTFGKLVYERIFSMCVDSRSVLPDHFSPENANDTAKETCLNWFFKIASIRELIPRF<br>YVEASILKCNKFLSKTGISECLPRLTCMIRGIGDPLVSVYARAYLCRVGMEVAPHLKETLNKNFFDFLLTFKQIHGDTVQ<br>NQLVVQGVELPSYLPLYPPAMDWIFQCISYHAPEALLTEMMERCKKLGNNALLLNSVMSAFRAEFIATRSMDFIGMIKE<br>CDESGFPKHLLFRSLGLNLALADPPESDRLQILNEAWKVITKLKNPQDYINCAEVWVEYTCKHFTKREVNTVLADVIKH<br>MTPDRAFEDSYPQLQLIIKKVIAHFHDFSVLFSVEKFLPFLDMFQKESVRVEVCKCIMDAFIKHQQEPTKDPVILNALLH<br>VCKTMHDSVNALTLEDEKRMLSYLINGFIKMVSFGRDFEQQLSFYVESRSMFCNLEPVLVQLIHSVNRLAMETRKVMK<br>GNHSRKTAAFVRACVAYCFITIPSLAGIFTRLNLYLHSGQVALANQCLSQADAFFKAAISLVPEVPKMINIDGKMRPSES<br>FLLEFLCNFFSTLLIVPDHPEHGVLFLVRELLNVIQDYTWEDNSDEKIRIYTCVLHLLSAMSQETYLYHIDKVDSNDSLYG<br>GDSKFLAENNKLCETVMAQILEHLKTLAKDEALKRQSSLGLSFFNSILAHGDLRNNKLNQLSVNLWHLAQRHGCADTR<br>TMVKTLEYIKKQSKQPDMTHLTELALRLPLQTRT<br>MASWSHPQFEKGGGSGGGSGGSAWSHPQFEKSSGENLYFQGMGTALDIKIKRANKVYHAGEVLSGVVVISSKDSV<br>QHQGVSLTMEGTVNLQLSAKSVGVFEAFYNSVKPIQIINSTIEMVKPGKFPSGKTEIPFEFPLHLKGNKVLYETYHGVFV<br>NIQYTLRCDMKRSLLAKDLTKTCEFIVHSAPQKGKFTPSPVDFTITPETLQNVKERALLPKFLLRGHLNSTNCVITQPLT<br>GELVVESSEAAIRSVELQLVRVETCGCAEGYARDATEIQNIQIADGDVCRGLSVPIYMVFPRLFTCPTLETTNFKVEFEV<br>NIVVLLHPDHLITENFPLKLCRI<br>MLVLVLGDLHIPHRCNSLPAKFKKLLVPGKIQHILCTGNLCTKESYDYLKTLAGDVHIVRGDFDENLNYPEQKVVTVGQ<br>FKIGLIHGHQVIPWGDMASLALLQRQFDVDILISGHTHKFEAFEHENKFYINPGSATGAYNALETNIIPSFVLMDIQASTV<br>VTYVYQLIGDDVKVERIEYKKP
**120**	> His-TEV-VPS35L-TwinStrep-TEV-VPS26C-VPS29-mKate2<br>MGSSHHHHHHSSGENLYFQGMAVFPWHSRNRNYKAEFASCRLEAVPLEFGDYHPLKPITVTESKTKKVNRKGSTSS<br>TSSSSSSSVVDPLSSVLDGTDPLSMFAATADPAALAAAMDSSRRKRDRDDNSVVGSDFEPWTNKRGEILARYTTTEK<br>LSINLFMGSEKGKAGTATLAMSEKVRTRLEELDDFEEGSQKELLNLTQQDYVNRIEELNQSLKDAWASDQKVKALKIVI<br>QCSKLLSDTSVIQFYPSKFVLITDILDTFGKLVYERIFSMCVDSRSVLPDHFSPENANDTAKETCLNWFFKIASIRELIPRF<br>YVEASILKCNKFLSKTGISECLPRLTCMIRGIGDPLVSVYARAYLCRVGMEVAPHLKETLNKNFFDFLLTFKQIHGDTVQ<br>NQLVVQGVELPSYLPLYPPAMDWIFQCISYHAPEALLTEMMERCKKLGNNALLLNSVMSAFRAEFIATRSMDFIGMIKE<br>CDESGFPKHLLFRSLGLNLALADPPESDRLQILNEAWKVITKLKNPQDYINCAEVWVEYTCKHFTKREVNTVLADVIKH<br>MTPDRAFEDSYPQLQLIIKKVIAHFHDFSVLFSVEKFLPFLDMFQKESVRVEVCKCIMDAFIKHQQEPTKDPVILNALLH<br>VCKTMHDSVNALTLEDEKRMLSYLINGFIKMVSFGRDFEQQLSFYVESRSMFCNLEPVLVQLIHSVNRLAMETRKVMK<br>GNHSRKTAAFVRACVAYCFITIPSLAGIFTRLNLYLHSGQVALANQCLSQADAFFKAAISLVPEVPKMINIDGKMRPSES<br>FLLEFLCNFFSTLLIVPDHPEHGVLFLVRELLNVIQDYTWEDNSDEKIRIYTCVLHLLSAMSQETYLYHIDKVDSNDSLYG<br>GDSKFLAENNKLCETVMAQILEHLKTLAKDEALKRQSSLGLSFFNSILAHGDLRNNKLNQLSVNLWHLAQRHGCADTR<br>TMVKTLEYIKKQSKQPDMTHLTELALRLPLQTRT

	MASWSHPQFEKGGGSGGGSGGSAWSHPQFEKSSGENLYFQGMGTALDIKIKRANKVYHAGEVLSGVVVISSKDSV QHQGVSLTMEGTVNLQLSAKSVGVFEAFYNSVKPIQIINSTIEMVKPGKFPSGKTEIPFEFPLHLKGNKVLYETYHGVFV NIQYTLRCDMKRSLLAKDLTKTCEFIVHSAPQKGKFTPSPVDFTITPETLQNVKERALLPKFLLRGHLNSTNCVITQPLT GELVVESSEAAIRSVELQLVRVETCGCAEGYARDATEIQNIQIADGDVCRGLSVPIYMVFPRLFTCPTLETTNFKVEFEV NIVVLLHPDHLITENFPLKLCRI MLVLVLGDLHIPHRCNSLPAKFKKLLVPGKIQHILCTGNLCTKESYDYLKTLAGDVHIVRGDFDENLNYPEQKVVTVGQ FKIGLIHGHQVIPWGDMASLALLQRQFDVDILISGHTHKFEAFEHENKFYINPGSATGAYNALETNIIPSFVLMDIQASTV VTYVYQLIGDDVKVERIEYKKPGGGGSSMVSELIKENMHMKLYMEGTVNNHHFKCTSEGEGKPYEGTQTMRIKAVEG GPLPFAFDILATSFMYGSKTFINHTQGIPDFFKQSFPEGFTWERVTTYEDGGVLTATQDTSLQDGCLIYNVKIRGVNFP SNGPVMQKKTLGWEASTETLYPADGGLEGRADMALKLVGGGHLICNLKTTYRSKKPAKNLKMPGVYYVDRRLERIKE ADKETYVEQHEVAVARYCDLPSKLGHR
**279**	> FlagS-EGFP<br>MDYKDDDDKKETAAAKFERQHMDSGGGGTSLEMVSKGEELFTGVVPILVELDGDVNGHKFSVSGEGEGDATYGKLT LKFICTTGKLPVPWPTLVTTLTYGVQCFSRYPDHMKQHDFFKSAMPEGYVQERTIFFKDDGNYKTRAEVKFEGDTLVN RIELKGIDFKEDGNILGHKLEYNYNSHNVYIMADKQKNGIKVNFKIRHNIEDGSVQLADHYQQNTPIGDGPVLLPDNHYL STQSALSKDPNEKRDHMVLLEFVTAAGITLGMDELYK
**284**	> FlagS-L2$_{FBR}$<br>MDYKDDDDKKETAAAKFERQHMDSGGGGTSPDPAFVTTPTKLITYDNPAYEGIDVDNTLYF
**285**	> FlagS-L2$_{FBR(N254A+Y257A)}$<br>MDYKDDDDKKETAAAKFERQHMDSGGGGTSPDPAFVTTPTKLITYDAPAAEGIDVDNTLYF
**286**	> FlagS-L2<br>MDYKDDDDKKETAAAKFERQHMDSGGGGTSPMRHKRSAKRTKRASATQLYKTCKQAGTCPPDIIPKVEGKTIAEQIL QYGSMGVFFGGLGIGTGSGTGGRTGYIPLGTRPPTATDTLAPVRPPLTVDPVGPSDPSIVSLVEETSFIDAGAPTSVPS IPPDVSGFSITTSTDTTPAILDINNTVTTVTTHNNPTFTDPSVLQPPTPAETGGHFTLSSSTISTHNYEEIPMDTFIVSTNP NTVTSSTPIPGSRPVARLGLYSRTTQQVKVVDPAFVTTPTKLITYDNPAYEGIDVDNTLYFSSNDNSINIAPDPDFLDIVA LHRPALTSRRTGIRYSRIGNKQTLRTRSGKSIGAKVHYYYDLSTIDPAEEIELQTITPSTYTTTSHAASPTSINNGLYDIYA DDFITDTSTTPVPSVPSTSLSGYIPANTTIPFGGAYNIPLVSGPDIPINITDQAPSLIPIVPGSPQYTIIADAGDFYLHPSYY MLRKRRKRLPYFFSDVSLAA
**287**	> FlagS-L2$_{(N254A+Y257A)}$<br>MDYKDDDDKKETAAAKFERQHMDSGGGGTSPMRHKRSAKRTKRASATQLYKTCKQAGTCPPDIIPKVEGKTIAEQIL QYGSMGVFFGGLGIGTGSGTGGRTGYIPLGTRPPTATDTLAPVRPPLTVDPVGPSDPSIVSLVEETSFIDAGAPTSVPS IPPDVSGFSITTSTDTTPAILDINNTVTTVTTHNNPTFTDPSVLQPPTPAETGGHFTLSSSTISTHNYEEIPMDTFIVSTNP NTVTSSTPIPGSRPVARLGLYSRTTQQVKVVDPAFVTTPTKLITYDAPAAEGIDVDNTLYFSSNDNSINIAPDPDFLDIVA LHRPALTSRRTGIRYSRIGNKQTLRTRSGKSIGAKVHYYYDLSTIDPAEEIELQTITPSTYTTTSHAASPTSINNGLYDIYA DDFITDTSTTPVPSVPSTSLSGYIPANTTIPFGGAYNIPLVSGPDIPINITDQAPSLIPIVPGSPQYTIIADAGDFYLHPSYY MLRKRRKRLPYFFSDVSLAA

Appendix Table S5. Steps for protein purification for each construct.

Identifier	Protein construct	Steps	Final buffer
pMLG31	GST	GST_1, TEV in roller, GST_2, S200 16/60	Hp150+TCEP
pMLG21	His-Sumo3-VPS26C	Ni_1, SenP2 + dialysis, Ni_2, Q, S75 10/300	Hp150+TCEP
pMLG6	GST-VPS29	GST_1, TEV + dialysis, GST_2, Q y S75 10/300	Hp150+TCEP
pMLG13	His-Sumo3-SNX17$_{FL}$	Ni_1, SenP2 + dialysis, Ni_2 y Q	Hp300+TCEP
pMLG135	His-Sumo3-SNX17$_{N108}$	Ni_1, Senp2 + dialysis, Ni_2, Q, S200 16/60	Hp300+TCEP
pMLG1	His-Sumo3-SNX17$_{FERM\text{-}CT}$	Ni_1, Senp2 + dialysis, Ni_2, Q, S200 16/60	Hp300+TCEP
pMLG14	His-Sumo3-EGFP-SNX17	Ni_1, Senp2 + dialysis, Ni_2, Q, S200 16/60	Hp300+TCEP
pMLG159	His-MBP-SNX17	Ni_1, TEV + dialysis, Ni_2, S200 16/60	Hp300+TCEP
pMLG179	His-MBP-SNX17$_{D467X}$	Ni_1, TEV + dialysis, Ni_2, S200 16/60	Hp300+TCEP
pMLG178	His-MBP-SNX17$_{L470G}$	Ni_1, TEV + dialysis, Ni_2, S200 16/60	Hp300+TCEP
pMLG203	His-MBP-SNX17$_{W321A}$	Ni_1, TEV + dialysis, Ni_2, S200 16/60	Hp300+TCEP
pMLG204	His-MBP-SNX17$_{V380D}$	Ni_1, TEV + dialysis, Ni_2, S200 16/60	Hp300+TCEP
pMLG205	His-MBP-SNX17$_{H457A}$	Ni_1, TEV + dialysis, Ni_2, S200 16/60	Hp300+TCEP
pMLG206	His-MBP-SNX17$_{N459A+F462A}$	Ni_1, TEV + dialysis, Ni_2, S200 16/60	Hp300+TCEP
pMLG18	GST-LRP1$_{ICD}$	GST_1, S200 16/60	Hp150+TCEP
pMLG275	GST-TEV-LRP1$_{ICD\text{-}mut\ (N4470A+Y4473A)}$	GST_1, S200 16/60	Hp150+TCEP
pMLG173	MBP-His$_{10}$-LRP1$_{ICD}$	Ni_1, TEV + dialysis, Ni_2, Q, S200 16/60	Hp200+TCEP
pMLG49	GST-APP$_{ICD}$	GST_1, S200 16/60	Hp150+TCEP
pMLG168	GST-TEV-ITGB1$_{ICD}$	GST_1, S200 16/60	Hp150+TCEP
pMLG28	GST-L2$_{FBR}$	GST_1, S200 16/60	Hp150+TCEP
pMLG276	GST-TEV-L2$_{FBR\text{-}mut\ (N254A+Y257A)}$	GST_1, S200 16/60	Hp150+TCEP
pMLG131	GST-3C-His$_{10}$-L2$_{FBR}$	GST_1, HRV 3C in roller, GST_2, Ni_1, S75 10/300	Hp200+TCEP
pMLG85	His-VPS35L-TwinStrep-VPS26C-VPS29	Ni_1, $Strept_1$, TEV in roller, Ni_2, $Strept_2$, S200 10/300	Hp300+TCEP
pMLG122	His-VPS35L-TwinStrep-VPS26C-GST-VPS29	Ni_1, TEV + dialysis, GST_1, S200 16/60	Hp300+TCEP
pMLG118	His-VPS35L$_{110\text{-}963}$-TwinStrep-VPS26C-VPS29	$Strept_1$, Ni_1, TEV + dialysis/in roller, Ni_2, S200 10/300	Hp300+TCEP
pMLG116	His-VPS35L$_{110\text{-}598}$-TwinStrep-VPS26C-VPS29	$Strept_1$, Ni_1, TEV + dialysis, Ni_2, S200 10/300	Hp300+TCEP
pMLG138	His-VPS35L$_{1\text{-}598}$-TwinStrep-VPS26C-VPS29	$Strept_1$, Ni_1, TEV + dialysis, Ni_2, S200 10/300	Hp200+TCEP
pMLG144	His-VPS35L$_{1\text{-}436}$-TwinStrep-VPS26C-VPS29	$Strept_1$, Ni_1, TEV + dialysis, Ni_2, S200 10/300	Hp200+TCEP
pMLG151	His-VPS35L-GST-VPS29	Ni_1, TEV + dialysis, Ni_2, GST_1, HRV 3C in roller, GST_2, S200 10/300	Hp200+TCEP
pMLG231	His-VPS35L$_{R248E+W280D}$-TwinStrep-VPS26C-VPS29	Ni_1, $Strept_1$, TEV in roller, Ni_2, S200 10/300	Hp300+TCEP
pMLG232	His-VPS35L$_{K157E+R161E}$-TwinStrep-VPS26C-VPS29	Ni_1, $Strept_1$, TEV in roller, Ni_2, S200 10/300	Hp300+TCEP
pMLG120	His-VPS35L-TwinStrep-VPS26C-VPS29-mKate2	Ni_1, $Strept_1$, TEV in roller, $Strept_2$, S200 10/300	Hp200+TCEP

Ni_1: Nickel-based affinity chromatography of His-tagged-protein.
Ni_2: Nickel-based affinity chromatography of His-tagged-protein after protease incubation.
$Strept_1$: Strep-Tactin XT-based affinity chromatography of TwinStrep-tagged-protein.
$Strept_2$: Strep-Tactin XT-based affinity chromatography of TwinStrep-tagged-protein after protease incubation.
GST_1: Glutathione S-transferase (GST)-based affinity chromatography of GST-tagged-protein.
GST_2: GST-based affinity chromatography of GST-tagged-protein after protease incubation.
$Amylose_1$: Amylose-based affinity chromatography of Maltose-Binding Protein (MBP)-tagged-protein.
TEV: Incubation with Tobacco Etch Virus (TEV) protease.
Senp2: Incubation with Sentrin-specific protease 2 (Senp2) protease.
HRV 3C: Incubation with Human Rhinovirus (HRV) 3C protease.
Q: Ion exchange chromatography using a gradient of NaCl.

S75 10/300: Size exclusion chromatography with HiLoad 10/300 Superdex 75 column.
S200 10/300: Size exclusion chromatography with HiLoad 10/300 Superdex 200 column.
S200 16/60: Size exclusion chromatography with HiLoad 16/60 Superdex 200 column
Hp200+TCEP: 25 mM Hepes pH 7.5, 200 mM NaCl and 1mM TCEP
Hp300+TCEP: 25 mM Hepes pH 7.5, 300 mM NaCl and 1mM TCEP
Tr200+TCEP: 50 mM Tris pH 7.5, 200 mM NaCl and 1mM TCEP

Appendix Table S6. DNA oligos used for RT-PCR.

RNA	Identifier	Sequence
$L2_{FBR}$	369	AAATTCGAACGCCAGCACAT
	370	ACATCAATGCCTTCGTACGC
$L2_{FBR(N254A+Y257A)}$	371	ACAAGAAAGAAACCGCTGCT
	372	ATCAGTTTGGTCGGGGTGG
L2 / $L2_{N254A+Y257A}$	373	ACACCGTTACTAGCAGCACT
	374	GCAGCGCTACGATATCAAGG
EGFP	375	AAGGGCATCGACTTCAAGGA
	376	CTTCTCGTTGGGGTCTTTGC
Actin	ACTB Fw	CATGTACGTTGCTATCCAGGC
	ACTB Rv	CTCCTTAATGTCACGCACGAT

References

Gammoh N, Florey O, Overholtzer M & Jiang X (2013) Interaction between FIP200 and ATG16L1 distinguishes ULK1 complex-dependent and -independent autophagy. *Nat Struct Mol Biol* 20: 144–149

Pei J & Grishin N V (2001) AL2CO: calculation of positional conservation in a protein sequence alignment

Romano-Moreno M, Rojas AL, Williamson CD, Gershlick DC, Lucas M, Isupov MN, Bonifacino JS, MacHner MP & Hierro A (2017) Molecular mechanism for the subversion of the retromer coat by the Legionella effector RidL. *Proc Natl Acad Sci U S A* 114: E11151–E11160

Sheffield P, Garrard S & Derewenda Z (1999) Overcoming expression and purification problems of RhoGDI using a family of 'parallel' expression vectors. *Protein Expr Purif* 15: 34–39

Weissmann F, Petzold G, VanderLinden R, Huis In't Veld PJ, Brown NG, Lampert F, Westermann S, Stark H, Schulman BA & Peters JM (2016) biGBac enables rapid gene assembly for the expression of large multisubunit protein complexes. *Proc Natl Acad Sci U S A* 113: E2564–E2569
