# Supplementary figures and images for: Selective cargo and membrane recognition by SNX17 regulates its interaction with Retriever
Source: EMBO Rep. 2024 Dec 9;26(2):470–93. doi: 10.1038/s44319-024-00340-1 (PMC11772769; doi:10.1038/s44319-024-00340-1)

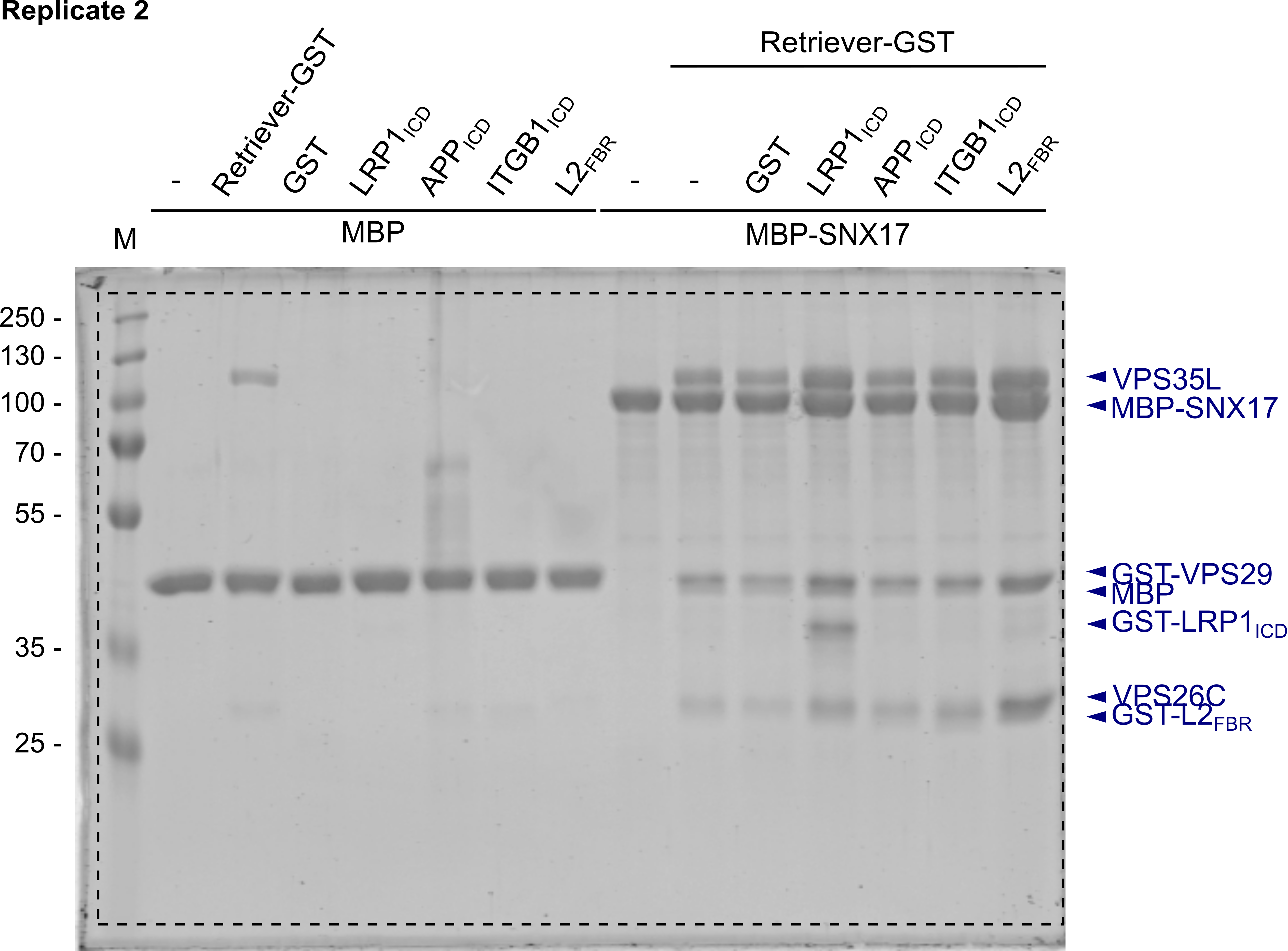

Supplement: Supplementary file 4 — Source data Fig. 2 [file 44319_2024_340_MOESM4_ESM.zip › Figure 2/2D/2D replicate 2.png]

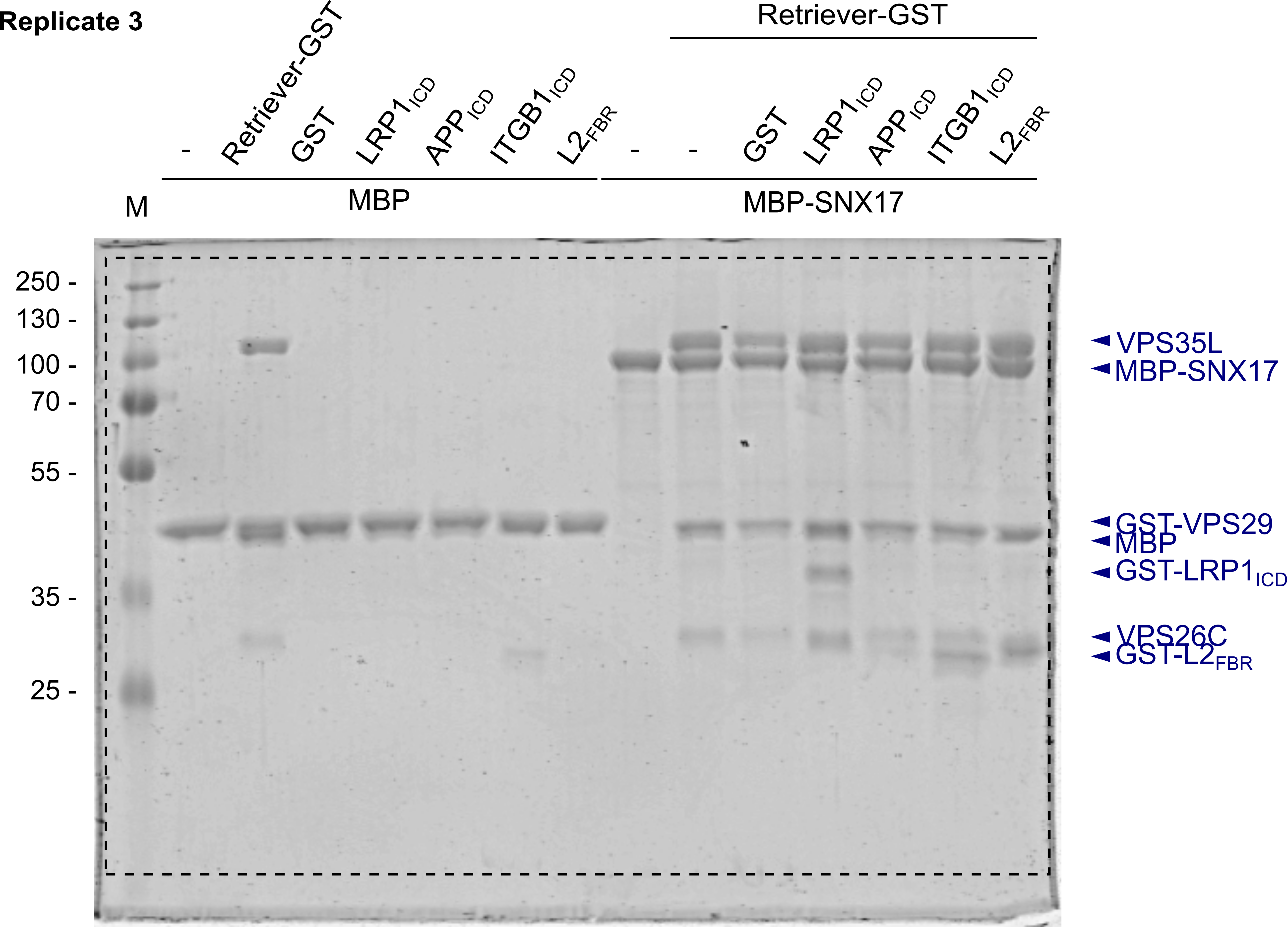

Supplement: Supplementary file 4 — Source data Fig. 2 [file 44319_2024_340_MOESM4_ESM.zip › Figure 2/2D/2D replicate 3.png]

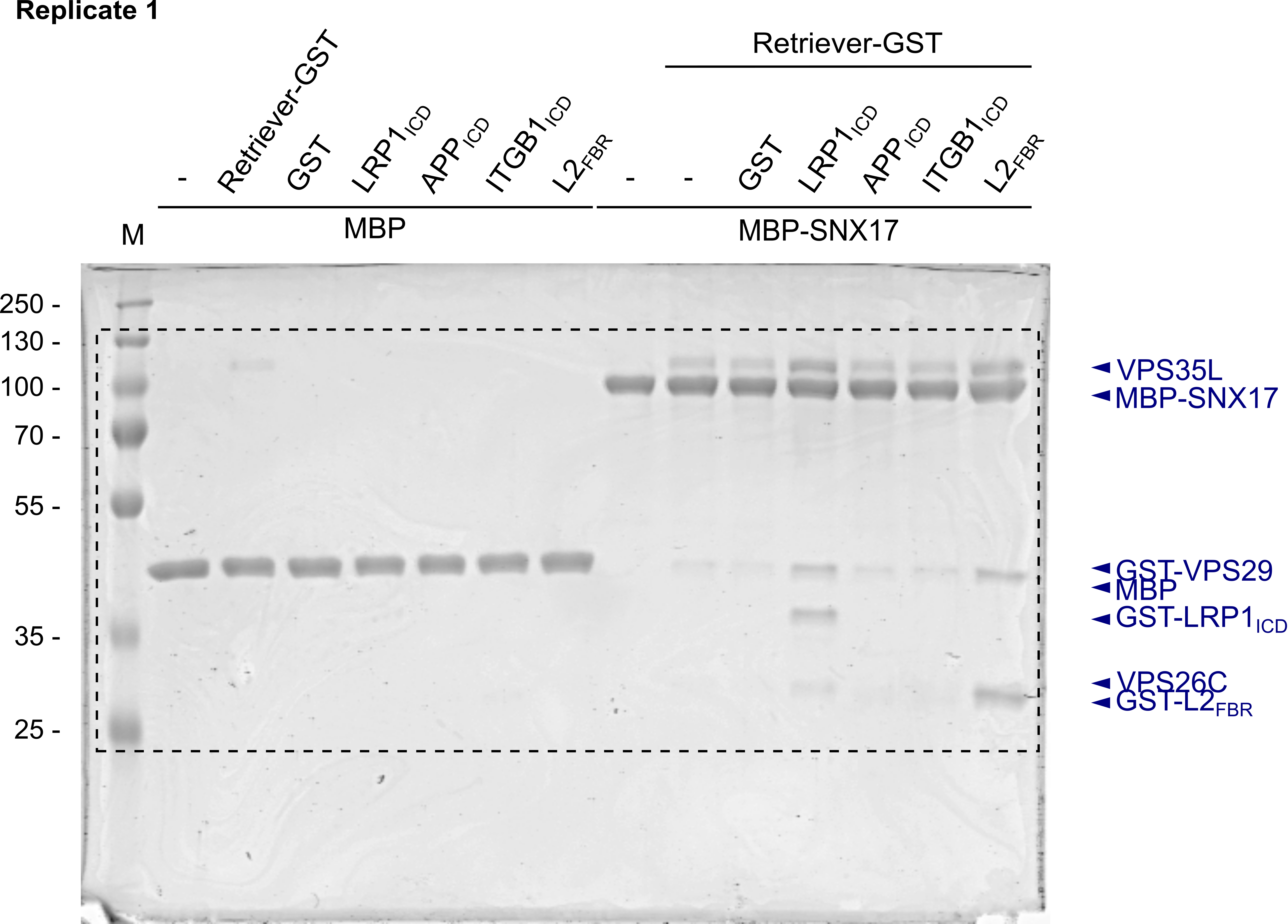

Supplement: Supplementary file 4 — Source data Fig. 2 [file 44319_2024_340_MOESM4_ESM.zip › Figure 2/2D/2D replicate 1.png]

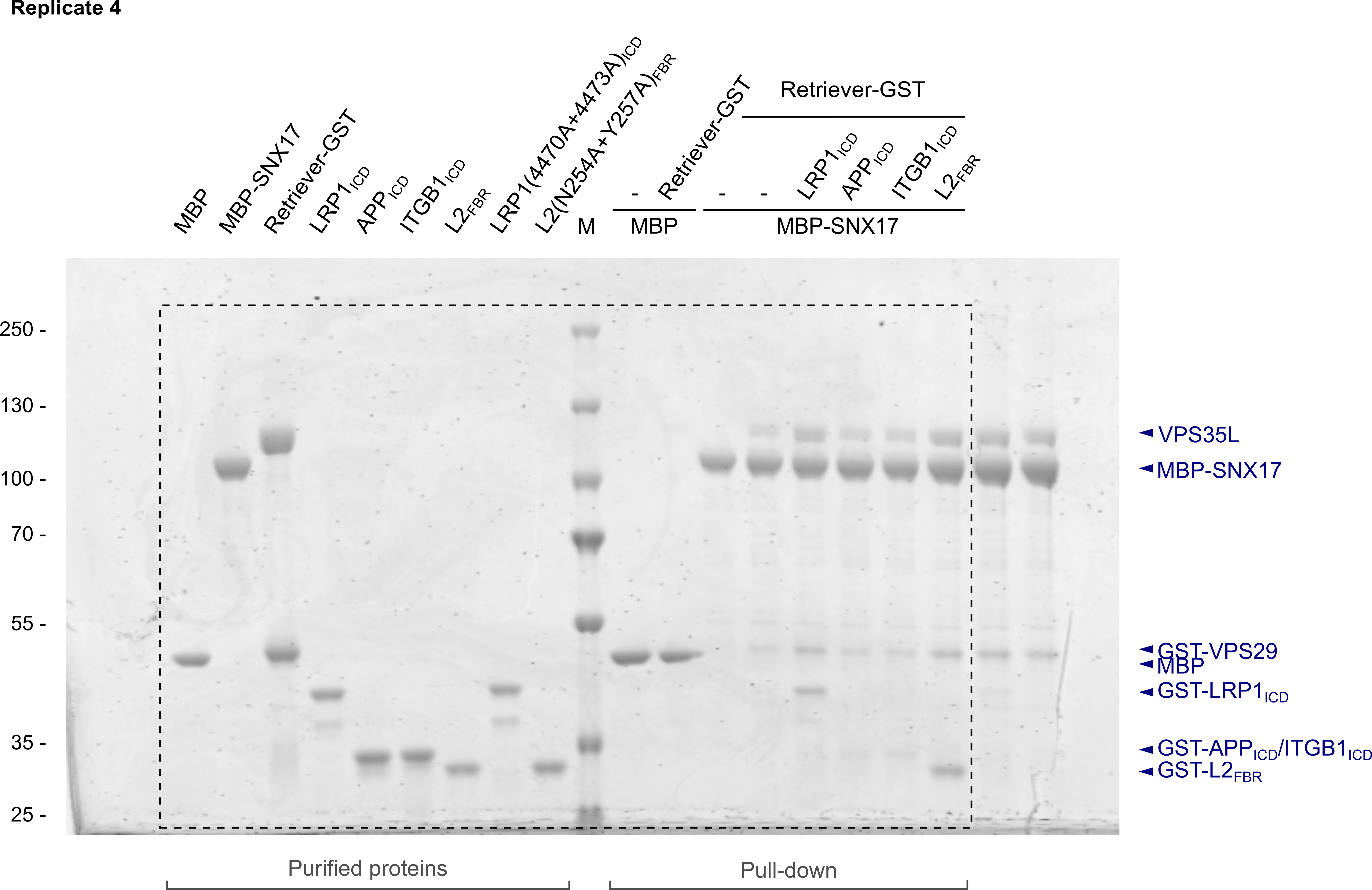

Supplement: Supplementary file 4 — Source data Fig. 2 [file 44319_2024_340_MOESM4_ESM.zip › Figure 2/2D/2D replicate 4.png]

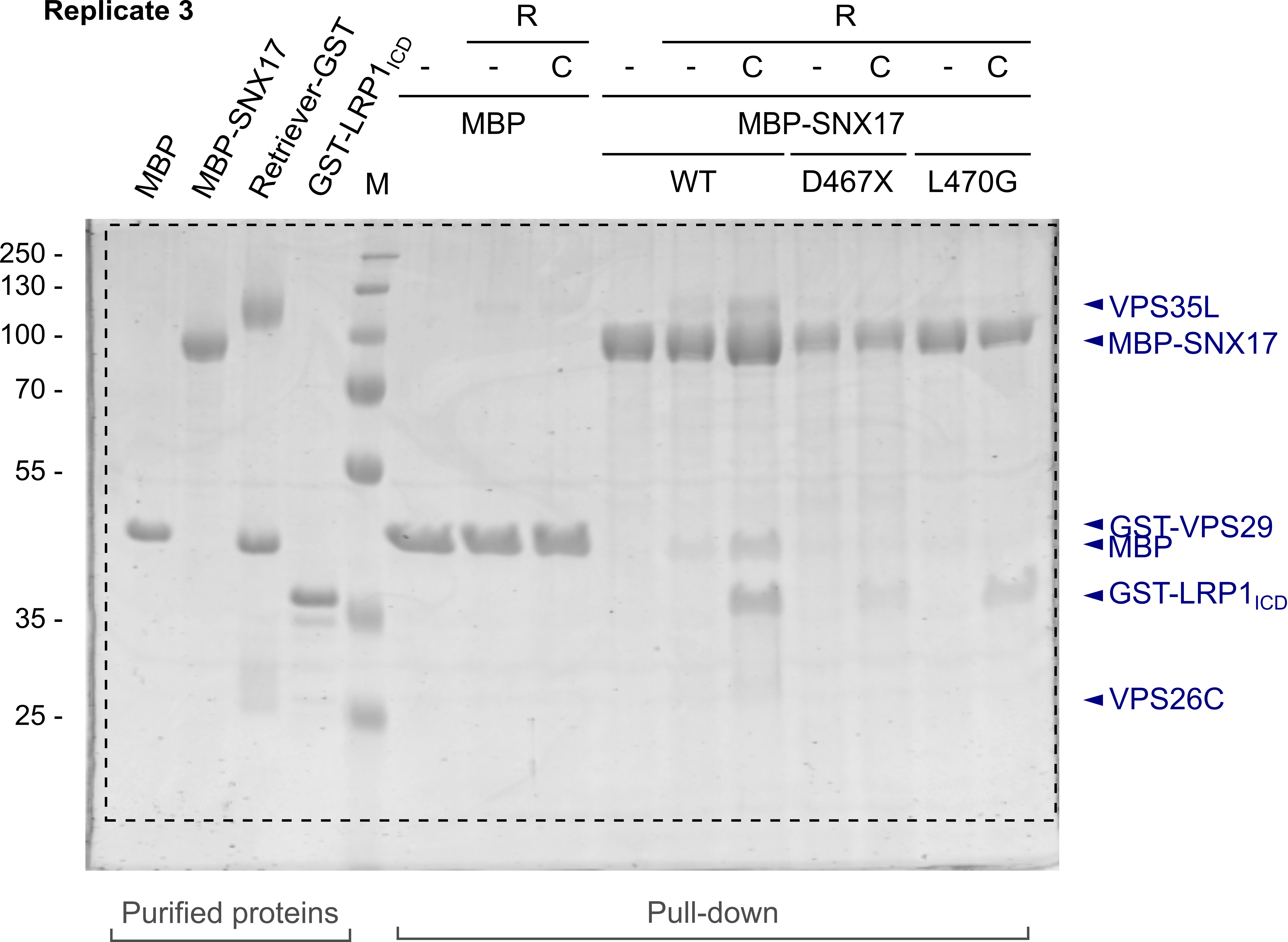

Supplement: Supplementary file 5 — Source data Fig. 3 [file 44319_2024_340_MOESM5_ESM.zip › Figure 3/3C/3C LRP1/3C LRP1_Replicate3.png]

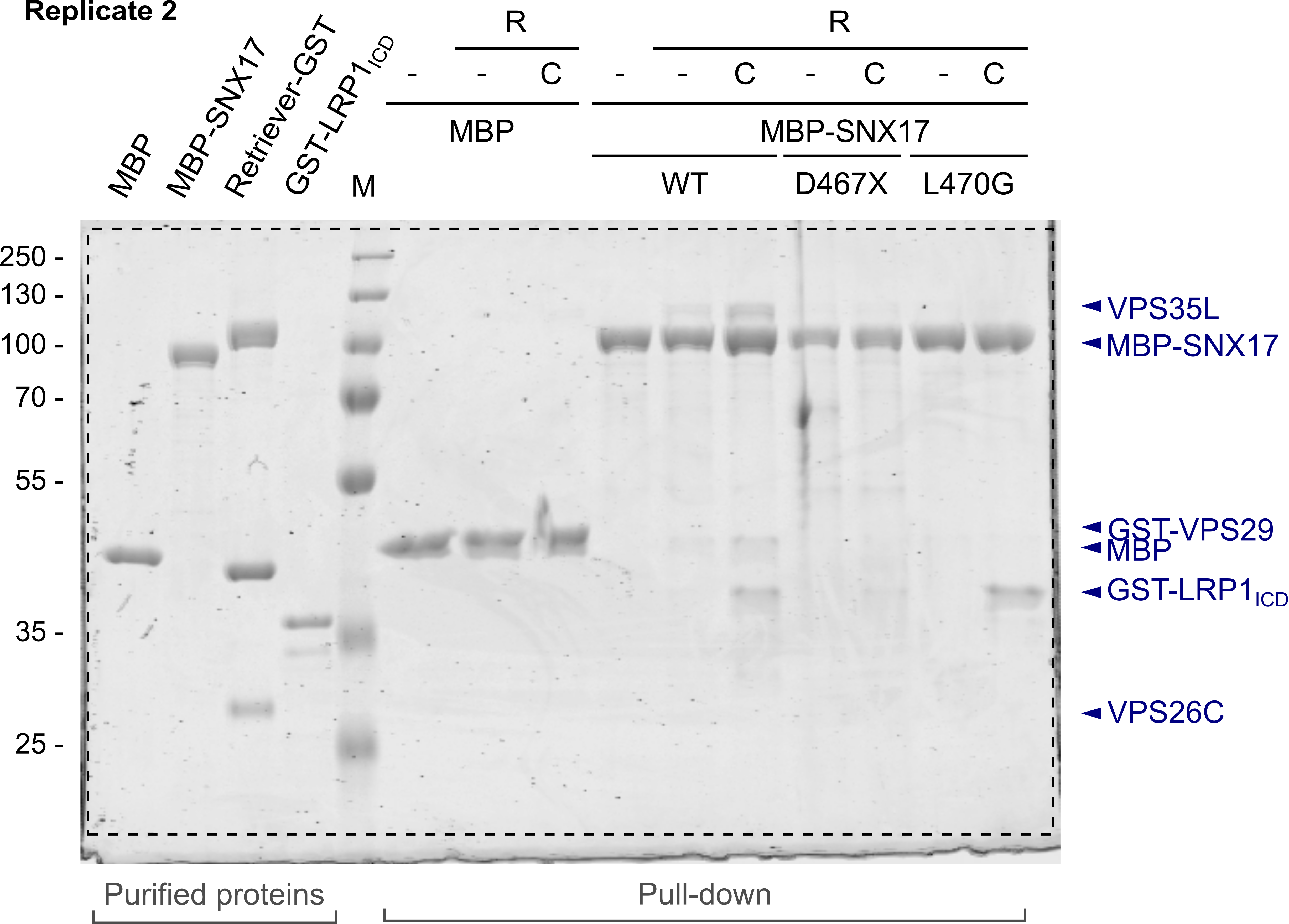

Supplement: Supplementary file 5 — Source data Fig. 3 [file 44319_2024_340_MOESM5_ESM.zip › Figure 3/3C/3C LRP1/3C LRP1_Replicate2.png]

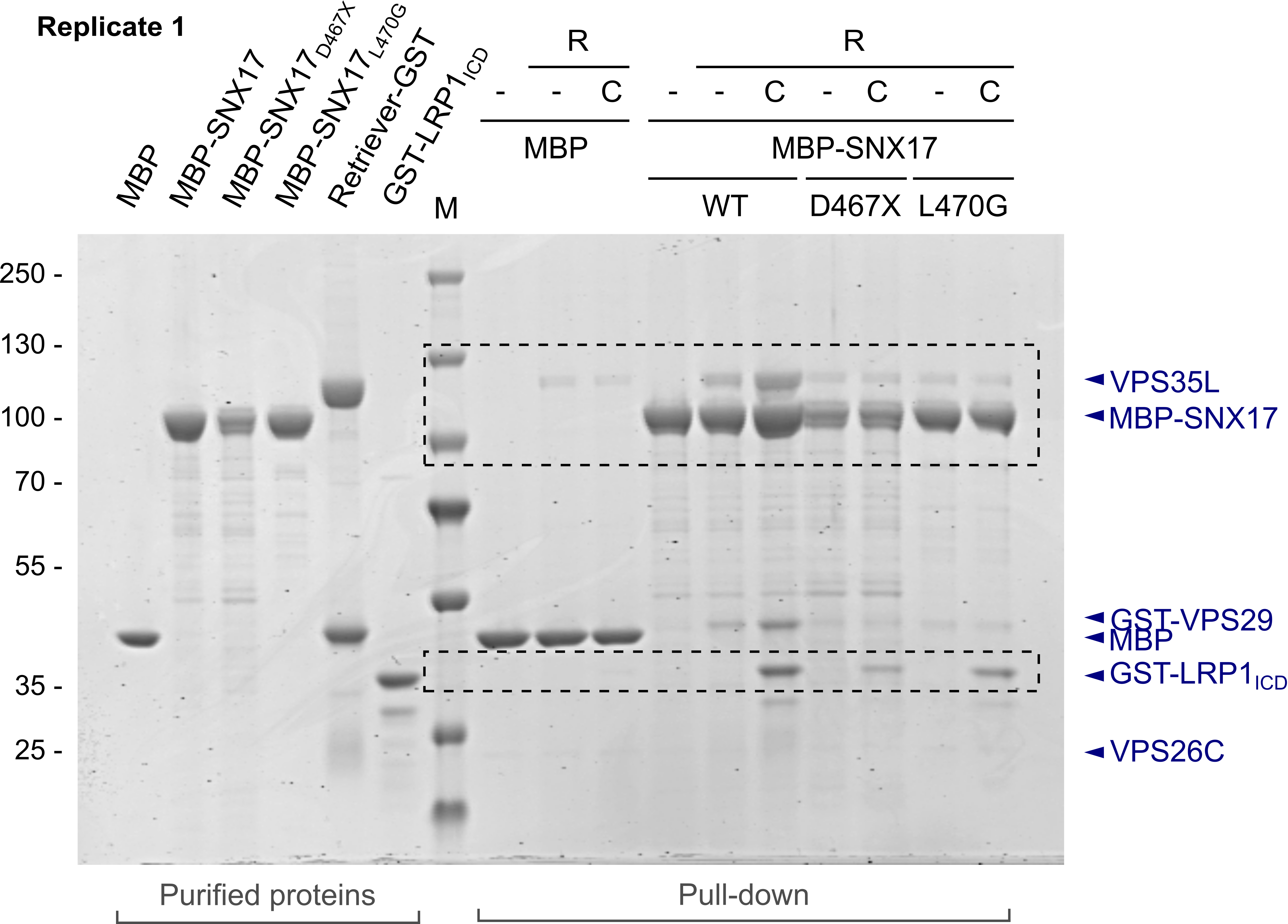

Supplement: Supplementary file 5 — Source data Fig. 3 [file 44319_2024_340_MOESM5_ESM.zip › Figure 3/3C/3C LRP1/3C LRP1_Replicate1.png]

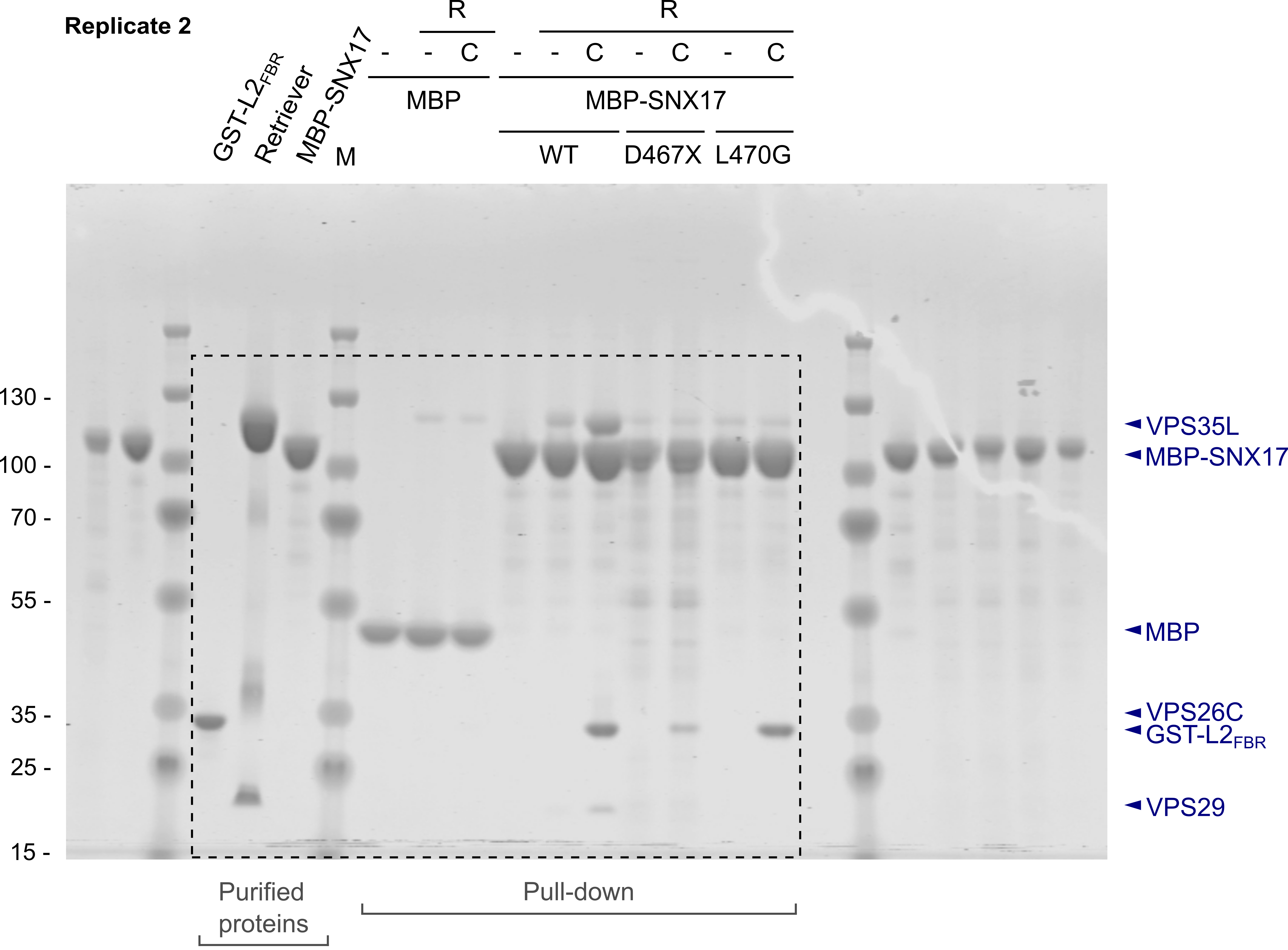

Supplement: Supplementary file 5 — Source data Fig. 3 [file 44319_2024_340_MOESM5_ESM.zip › Figure 3/3C/3C L2/3C L2_Replicate2.png]

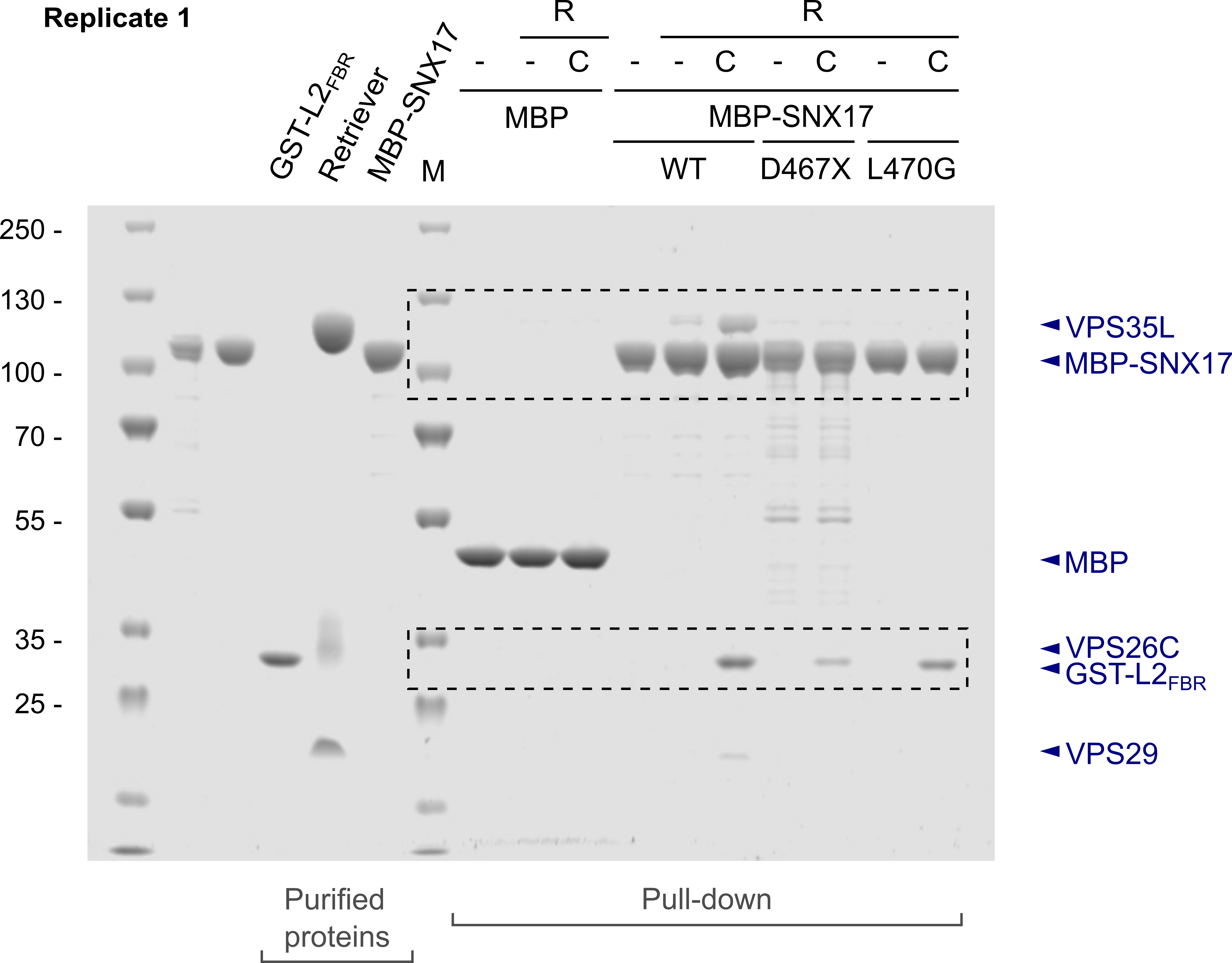

Supplement: Supplementary file 5 — Source data Fig. 3 [file 44319_2024_340_MOESM5_ESM.zip › Figure 3/3C/3C L2/3C L2_Replicate1.png]

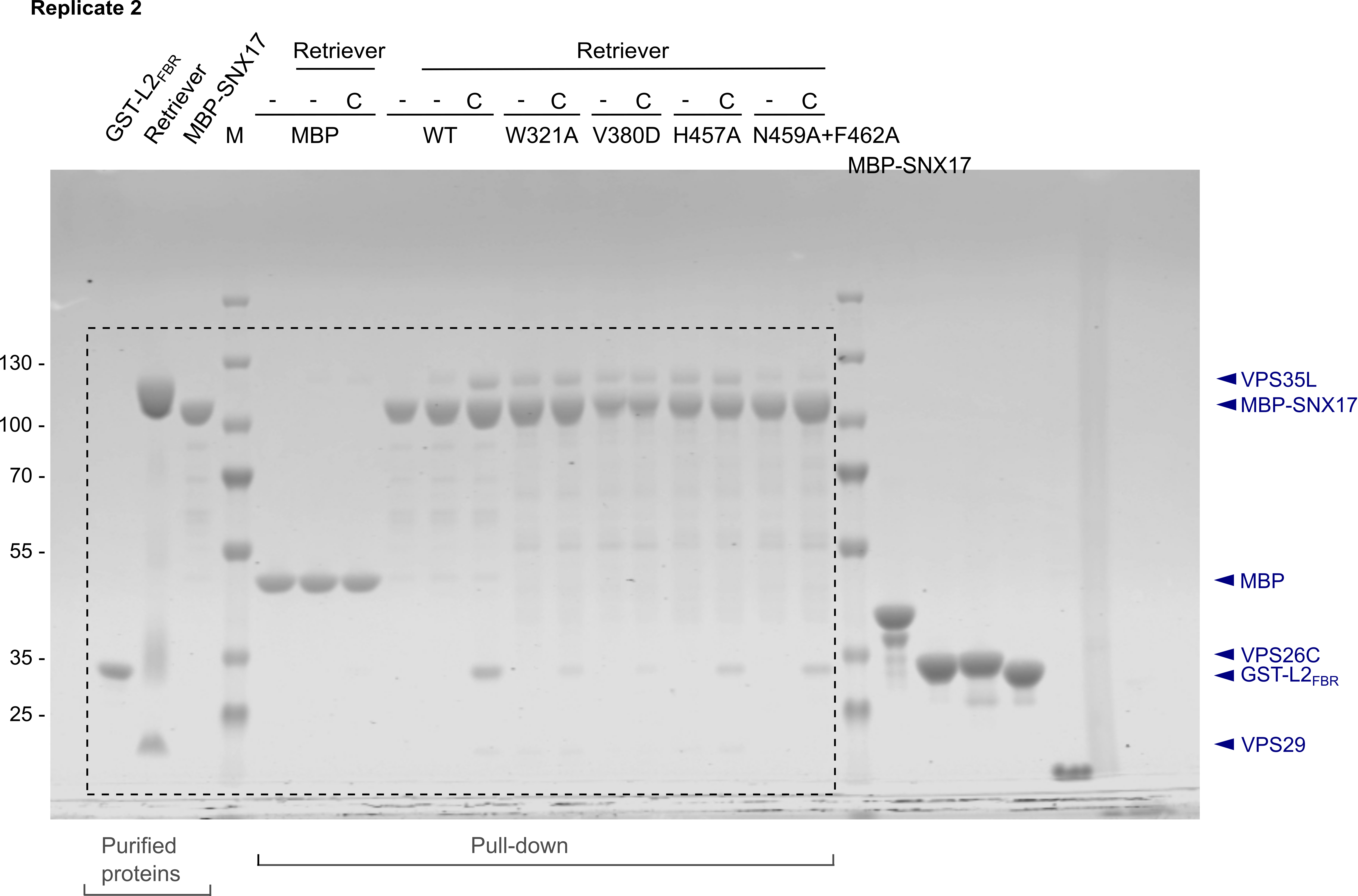

Supplement: Supplementary file 6 — Source data Fig. 4 [file 44319_2024_340_MOESM6_ESM.zip › Figure 4/4F/4F L2/4F L2_Replicate2.png]

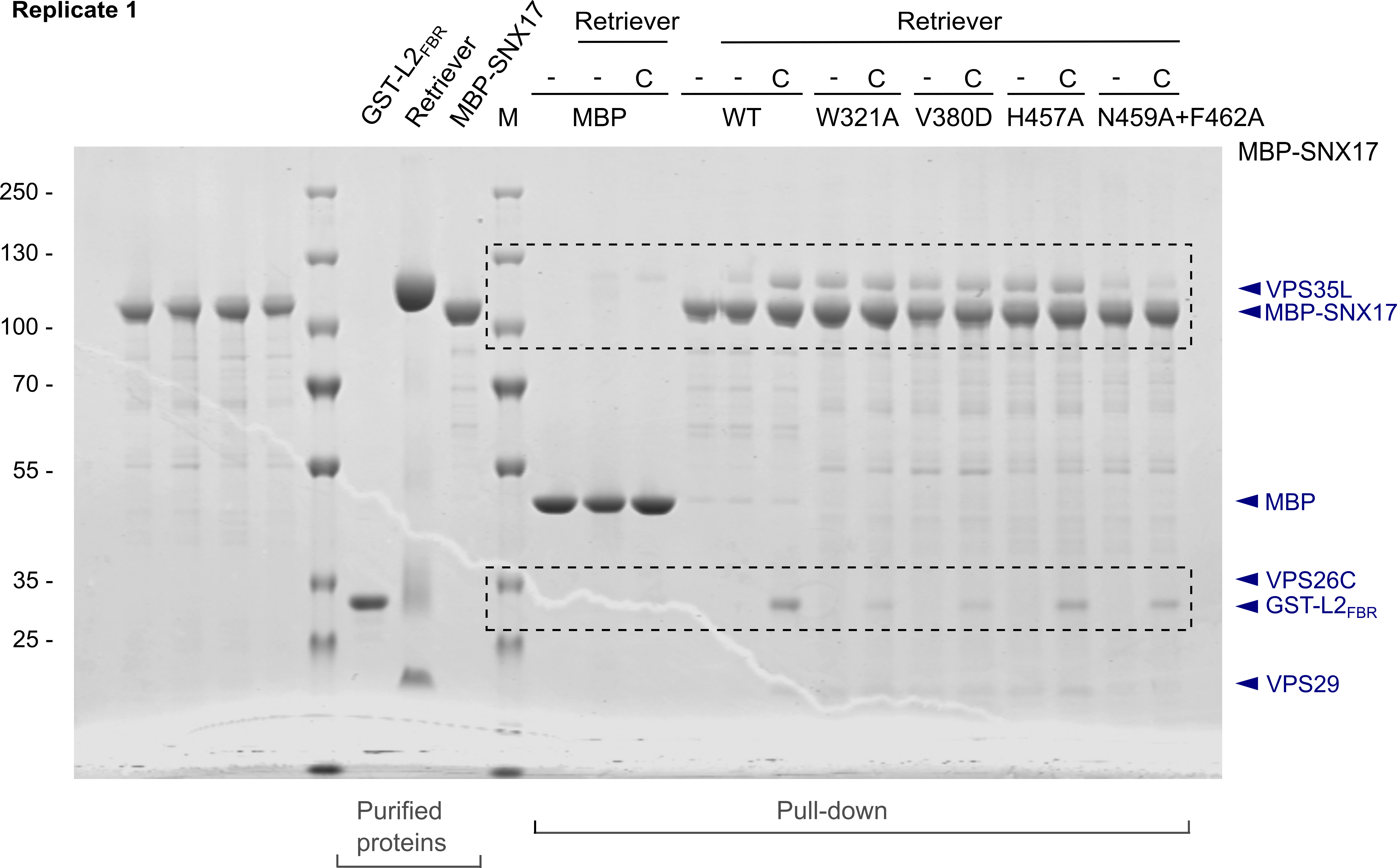

Supplement: Supplementary file 6 — Source data Fig. 4 [file 44319_2024_340_MOESM6_ESM.zip › Figure 4/4F/4F L2/4F L2_Replicate1.png]

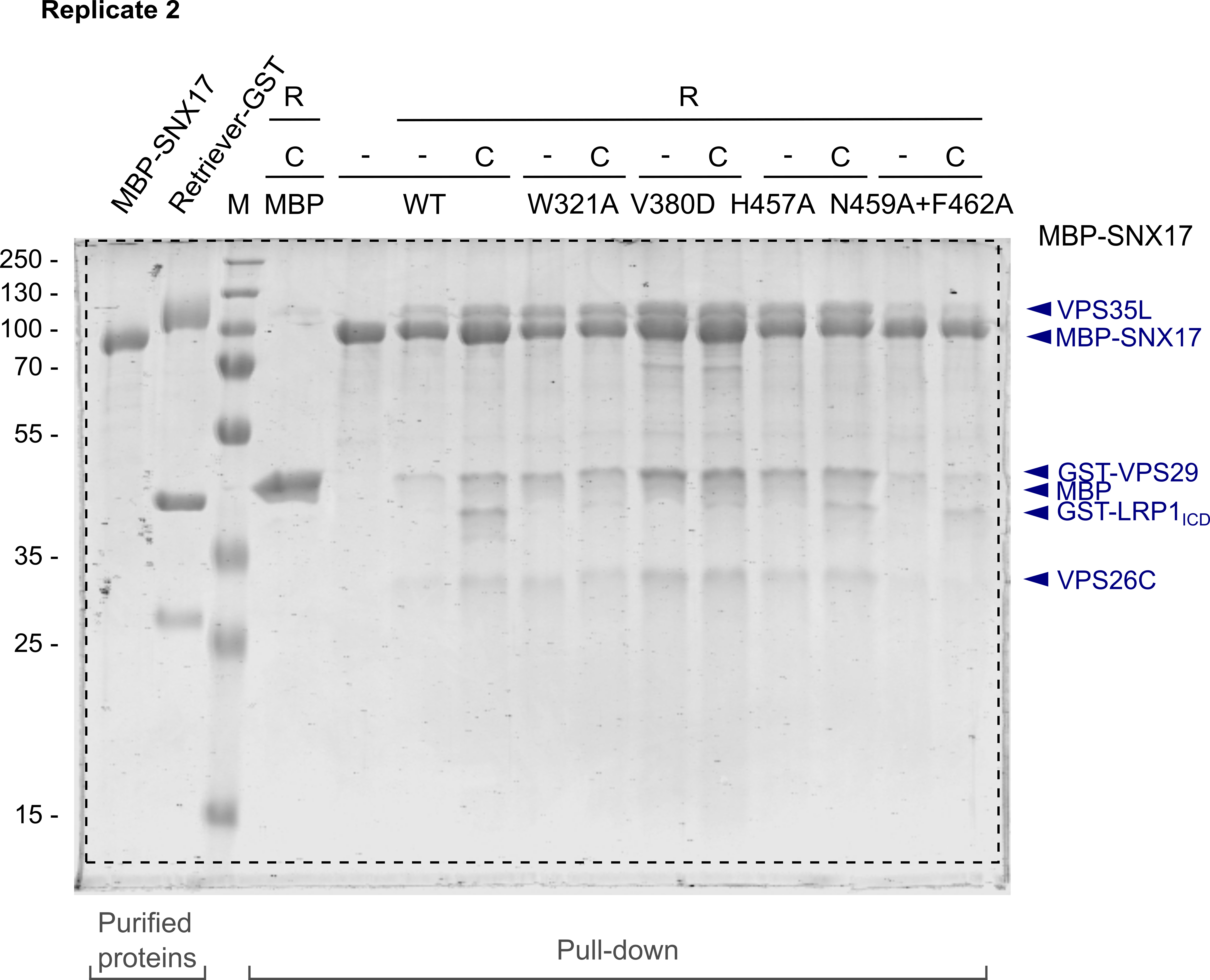

Supplement: Supplementary file 6 — Source data Fig. 4 [file 44319_2024_340_MOESM6_ESM.zip › Figure 4/4F/4F LRP1/4F LRP1_Replicate2.png]

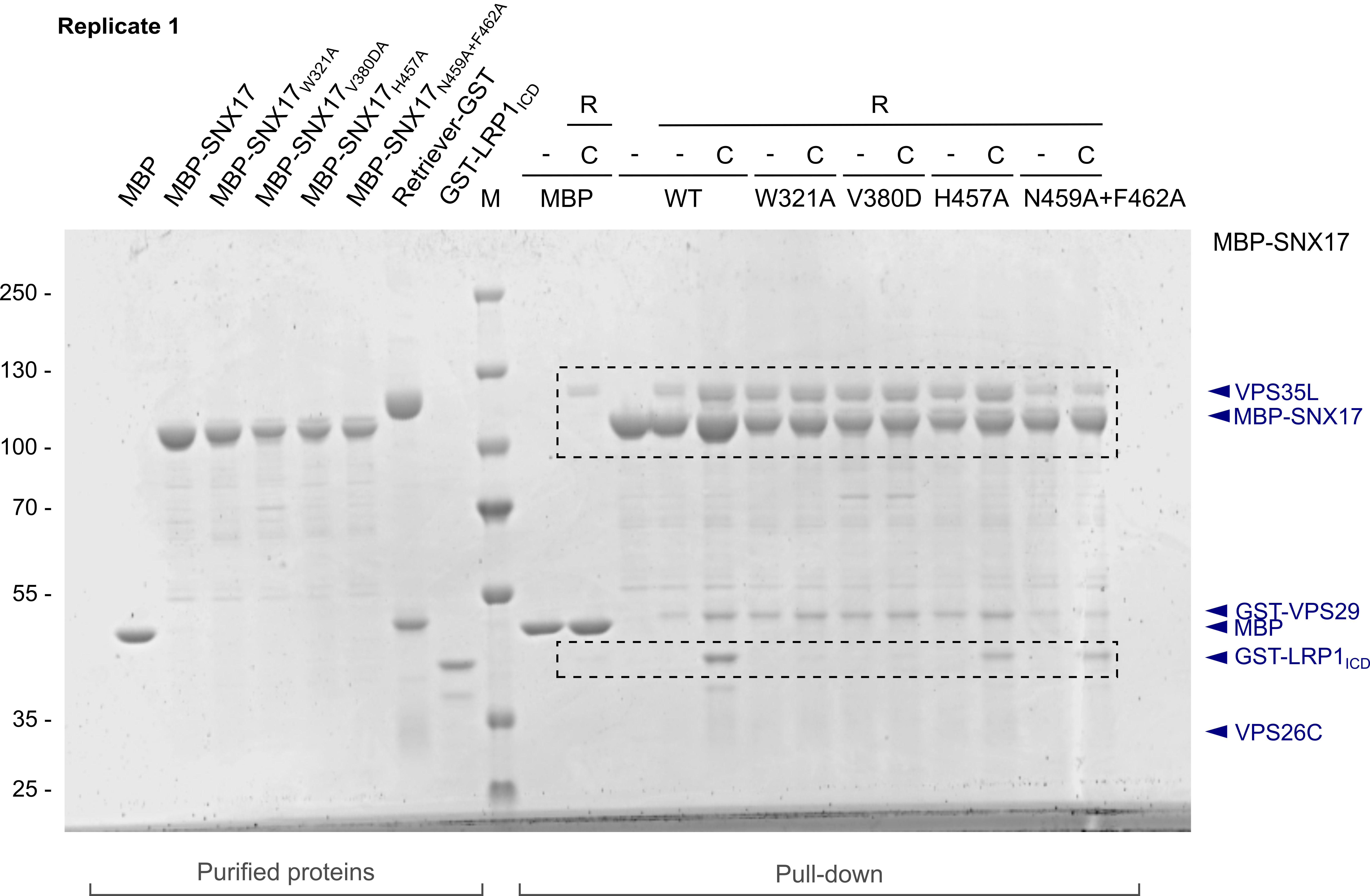

Supplement: Supplementary file 6 — Source data Fig. 4 [file 44319_2024_340_MOESM6_ESM.zip › Figure 4/4F/4F LRP1/4F LRP1_Replicate1.png]

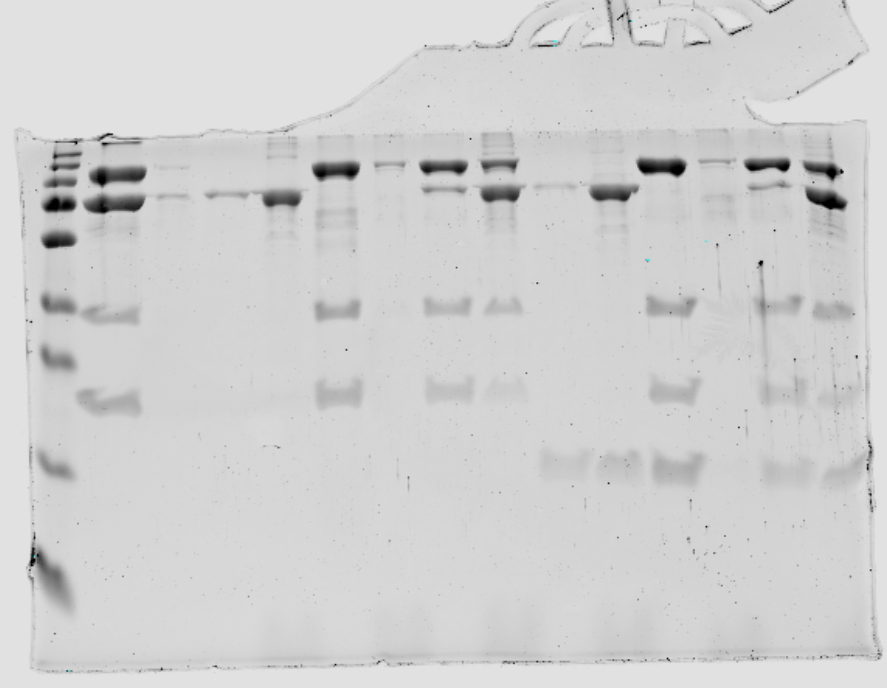

Supplement: Supplementary file 7 — Source data Fig. 5 [file 44319_2024_340_MOESM7_ESM.zip › Figure 5/5C/5C replicate1.png]

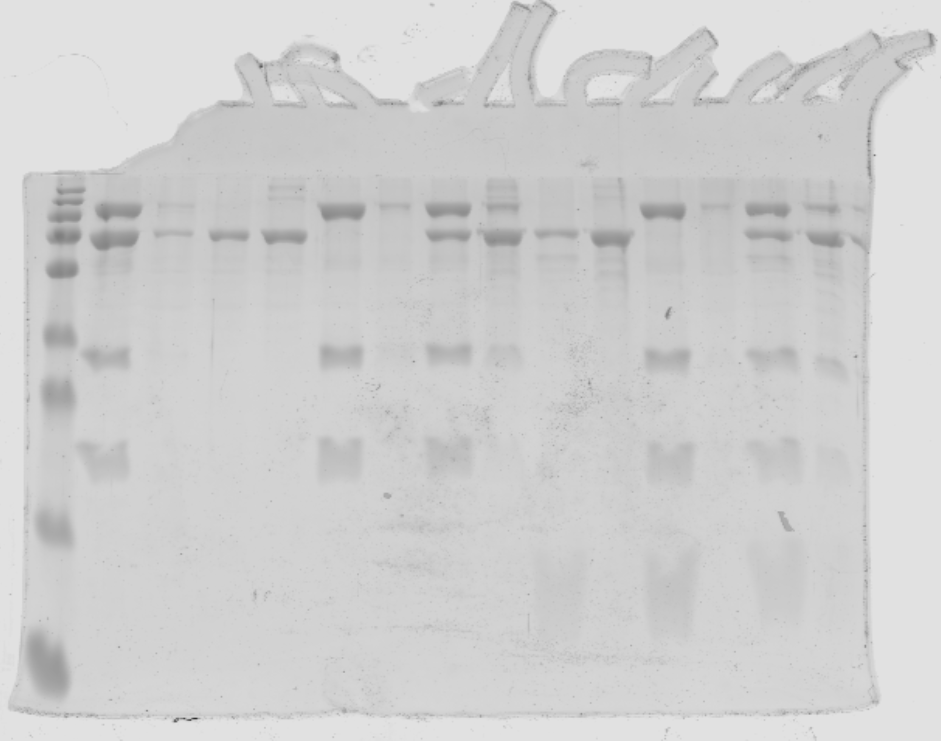

Supplement: Supplementary file 7 — Source data Fig. 5 [file 44319_2024_340_MOESM7_ESM.zip › Figure 5/5C/5C replicate3.png]

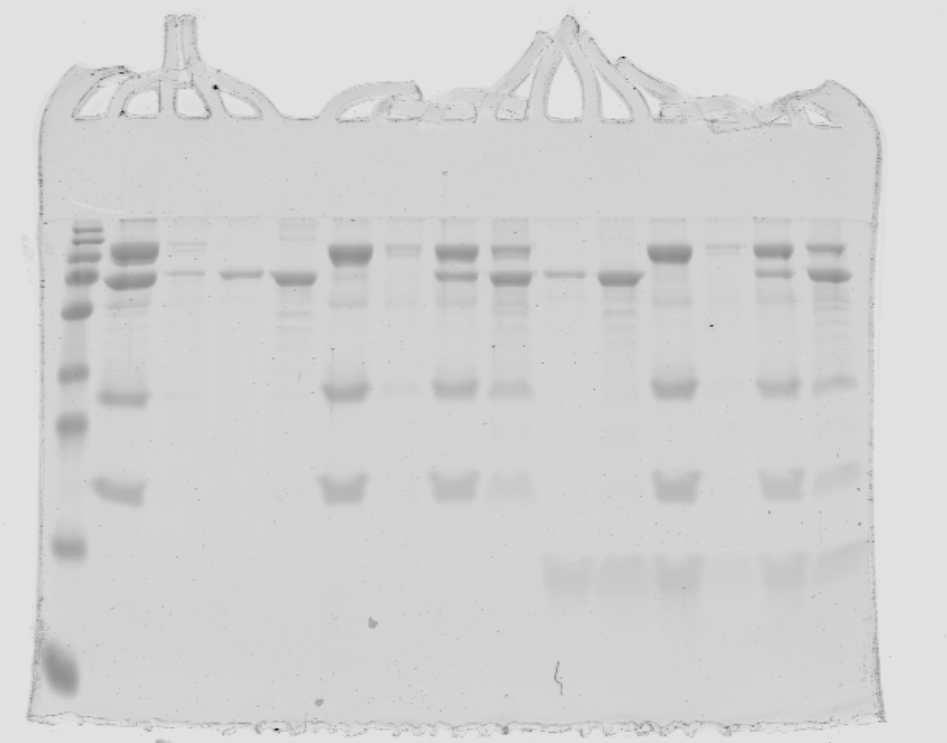

Supplement: Supplementary file 7 — Source data Fig. 5 [file 44319_2024_340_MOESM7_ESM.zip › Figure 5/5C/5C replicate2.png]

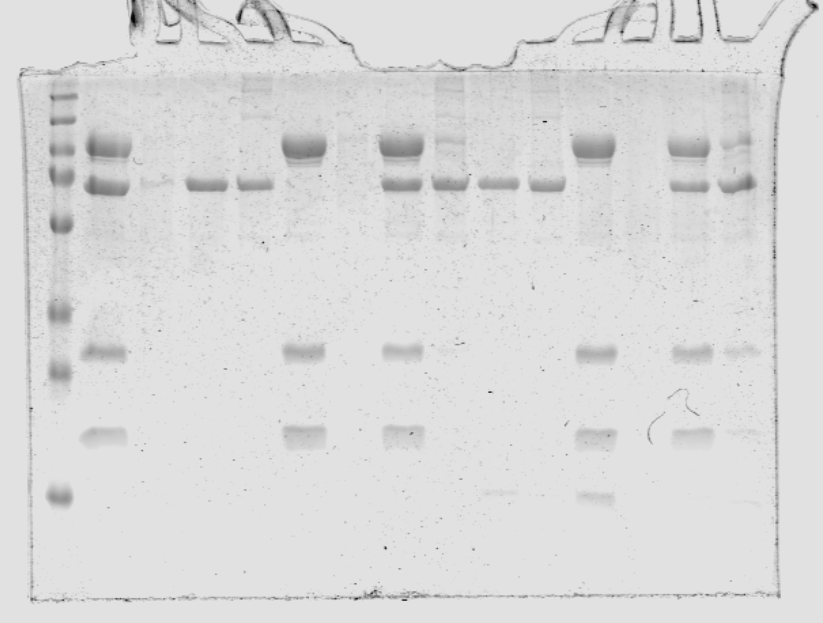

Supplement: Supplementary file 7 — Source data Fig. 5 [file 44319_2024_340_MOESM7_ESM.zip › Figure 5/5C/5C replicate6.png]

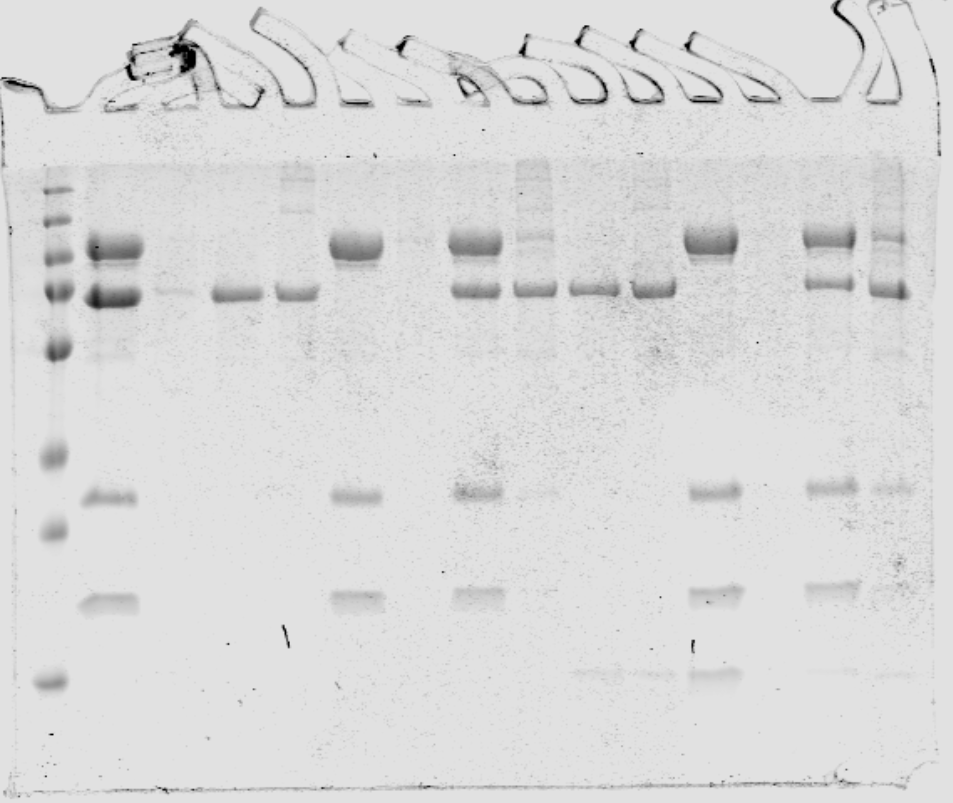

Supplement: Supplementary file 7 — Source data Fig. 5 [file 44319_2024_340_MOESM7_ESM.zip › Figure 5/5C/5C replicate5.png]

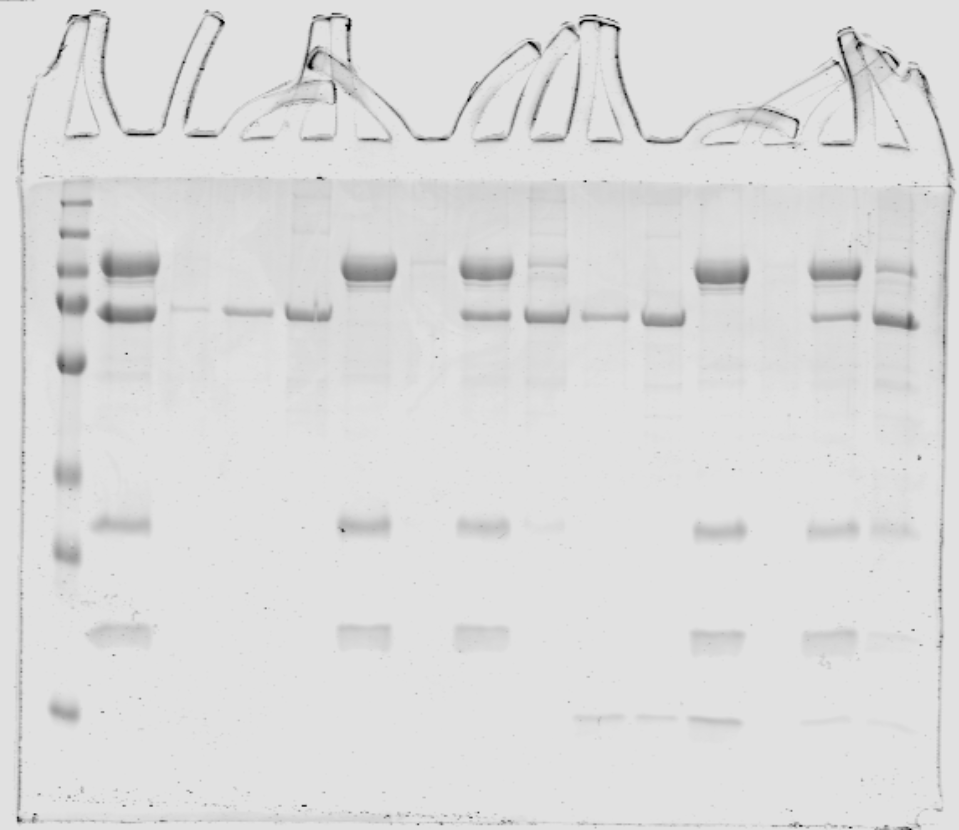

Supplement: Supplementary file 7 — Source data Fig. 5 [file 44319_2024_340_MOESM7_ESM.zip › Figure 5/5C/5C replicate4.png]

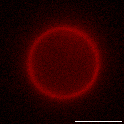

Supplement: Supplementary file 7 — Source data Fig. 5 [file 44319_2024_340_MOESM7_ESM.zip › Figure 5/5A/GUVs + GFP-SNX17 + Retriever-mKate2 + 10His-L2/GUVs+GFP-SNX17+Retriever-mKate2+10His-L2 red.png]

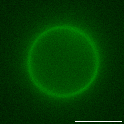

Supplement: Supplementary file 7 — Source data Fig. 5 [file 44319_2024_340_MOESM7_ESM.zip › Figure 5/5A/GUVs + GFP-SNX17 + Retriever-mKate2 + 10His-L2/GUVs+GFP-SNX17+Retriever-mKate2+10His-L2 green.png]

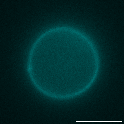

Supplement: Supplementary file 7 — Source data Fig. 5 [file 44319_2024_340_MOESM7_ESM.zip › Figure 5/5A/GUVs + GFP-SNX17 + Retriever-mKate2 + 10His-L2/GUVs+GFP-SNX17+Retriever-mKate2+10His-L2 cyan.png]

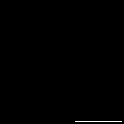

Supplement: Supplementary file 7 — Source data Fig. 5 [file 44319_2024_340_MOESM7_ESM.zip › Figure 5/5A/GUVs + Retriever-mKate2/GUVs+Retriever-mKate2 green.png]

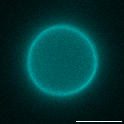

Supplement: Supplementary file 7 — Source data Fig. 5 [file 44319_2024_340_MOESM7_ESM.zip › Figure 5/5A/GUVs + Retriever-mKate2/GUVs+Retriever-mKate2 cyan.png]

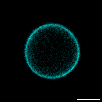

Supplement: Supplementary file 7 — Source data Fig. 5 [file 44319_2024_340_MOESM7_ESM.zip › Figure 5/5A/GUVs + 10His-L2/GUVs+10His-L2 blue.png]

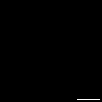

Supplement: Supplementary file 7 — Source data Fig. 5 [file 44319_2024_340_MOESM7_ESM.zip › Figure 5/5A/GUVs + 10His-L2/GUVs+10His-L2 green.png]

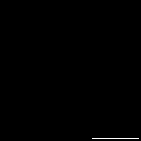

Supplement: Supplementary file 7 — Source data Fig. 5 [file 44319_2024_340_MOESM7_ESM.zip › Figure 5/5A/GUVs + Retriever-mKate2 + 10His-L2/GUVs+Retriever-mKate2+10His-L2 red.png]

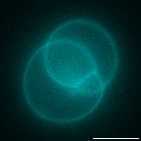

Supplement: Supplementary file 7 — Source data Fig. 5 [file 44319_2024_340_MOESM7_ESM.zip › Figure 5/5A/GUVs + Retriever-mKate2 + 10His-L2/GUVs+Retriever-mKate2+10His-L2 cyan.png]

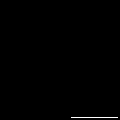

Supplement: Supplementary file 7 — Source data Fig. 5 [file 44319_2024_340_MOESM7_ESM.zip › Figure 5/5A/GUVs + GFP-SNX17 + 10His-L2/GUVs+GFP-SNX17+10His-L2 red.png]

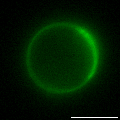

Supplement: Supplementary file 7 — Source data Fig. 5 [file 44319_2024_340_MOESM7_ESM.zip › Figure 5/5A/GUVs + GFP-SNX17 + 10His-L2/GUVs+GFP-SNX17+10His-L2 green.png]

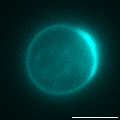

Supplement: Supplementary file 7 — Source data Fig. 5 [file 44319_2024_340_MOESM7_ESM.zip › Figure 5/5A/GUVs + GFP-SNX17 + 10His-L2/GUVs+GFP-SNX17+10His-L2 cyan.png]

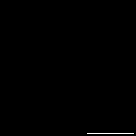

Supplement: Supplementary file 7 — Source data Fig. 5 [file 44319_2024_340_MOESM7_ESM.zip › Figure 5/5A/GUVs/GUVs green.png]

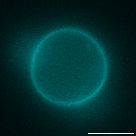

Supplement: Supplementary file 7 — Source data Fig. 5 [file 44319_2024_340_MOESM7_ESM.zip › Figure 5/5A/GUVs/GUVs cyan.png]

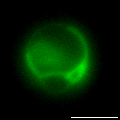

Supplement: Supplementary file 7 — Source data Fig. 5 [file 44319_2024_340_MOESM7_ESM.zip › Figure 5/5A/GUVs + GFP-SNX17/GUVs+GFP-SNX17 green.png]

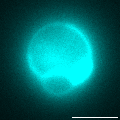

Supplement: Supplementary file 7 — Source data Fig. 5 [file 44319_2024_340_MOESM7_ESM.zip › Figure 5/5A/GUVs + GFP-SNX17/GUVs+GFP-SNX17 cyan.png]

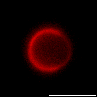

Supplement: Supplementary file 7 — Source data Fig. 5 [file 44319_2024_340_MOESM7_ESM.zip › Figure 5/5A/GUVs + GFP-SNX17 + Retriever-mKate2/GUVs+GFP-SNX17+Retriever-mKate2 red.png]

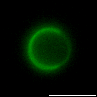

Supplement: Supplementary file 7 — Source data Fig. 5 [file 44319_2024_340_MOESM7_ESM.zip › Figure 5/5A/GUVs + GFP-SNX17 + Retriever-mKate2/GUVs+GFP-SNX17+Retriever-mKate2 green.png]

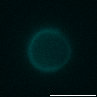

Supplement: Supplementary file 7 — Source data Fig. 5 [file 44319_2024_340_MOESM7_ESM.zip › Figure 5/5A/GUVs + GFP-SNX17 + Retriever-mKate2/GUVs+GFP-SNX17+Retriever-mKate2 cyan.png]

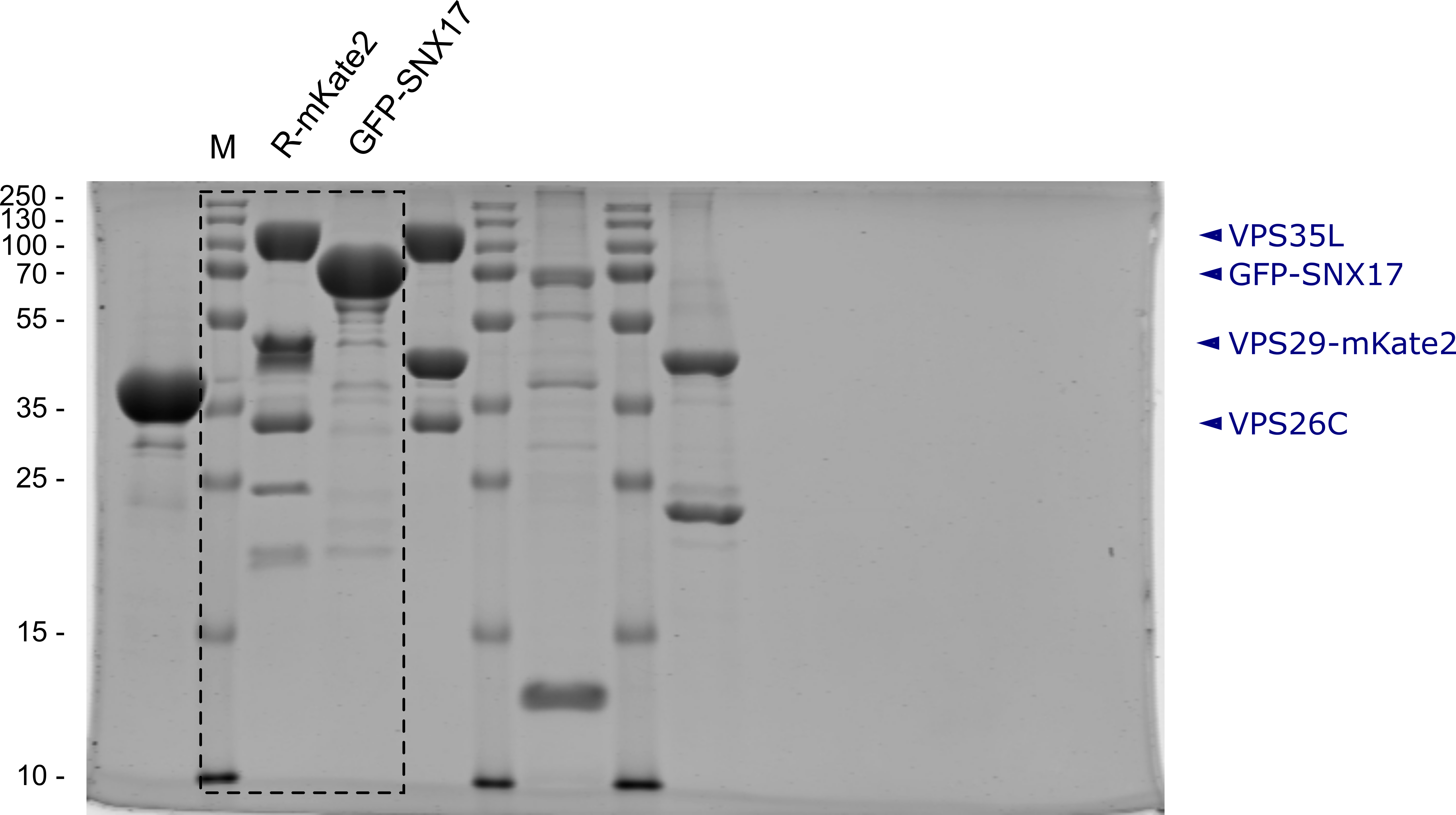

Supplement: Supplementary file 8 — EV and Appendix Figures Source Data [file 44319_2024_340_MOESM8_ESM.zip › EMBOR-2024-59048V3_SourceDataForExpandedView+Appendix/Figure S12/S12A/S12A gel.png]

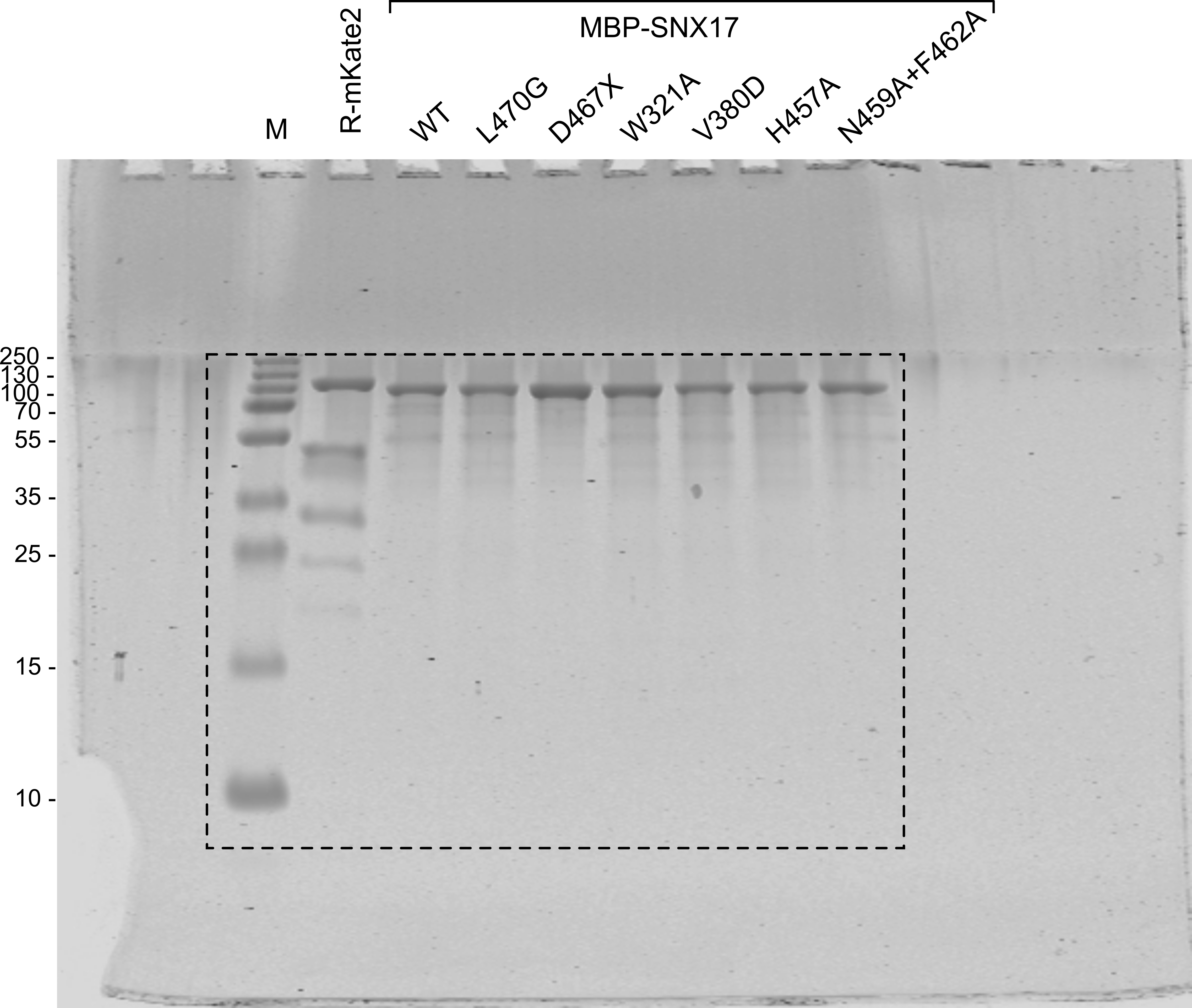

Supplement: Supplementary file 8 — EV and Appendix Figures Source Data [file 44319_2024_340_MOESM8_ESM.zip › EMBOR-2024-59048V3_SourceDataForExpandedView+Appendix/Figure S12/S12B/S12B gel.png]

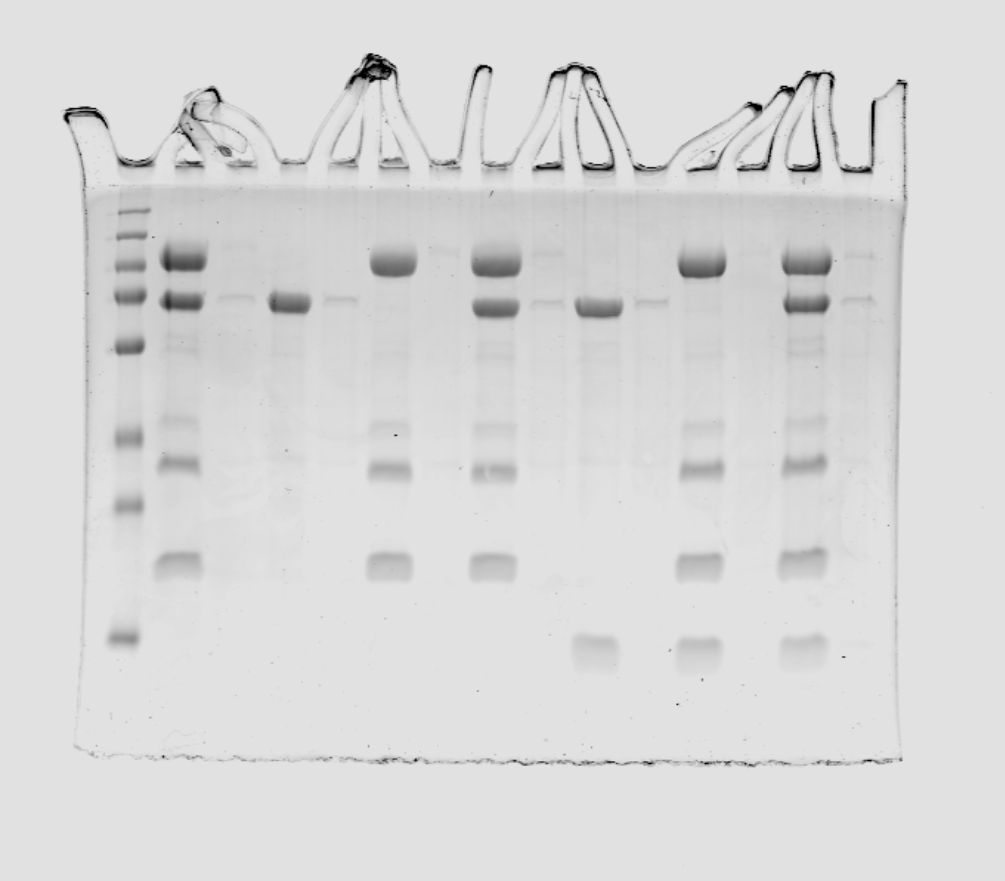

Supplement: Supplementary file 8 — EV and Appendix Figures Source Data [file 44319_2024_340_MOESM8_ESM.zip › EMBOR-2024-59048V3_SourceDataForExpandedView+Appendix/Figure EV4/EV4D/EV4D Replicate 1.png]

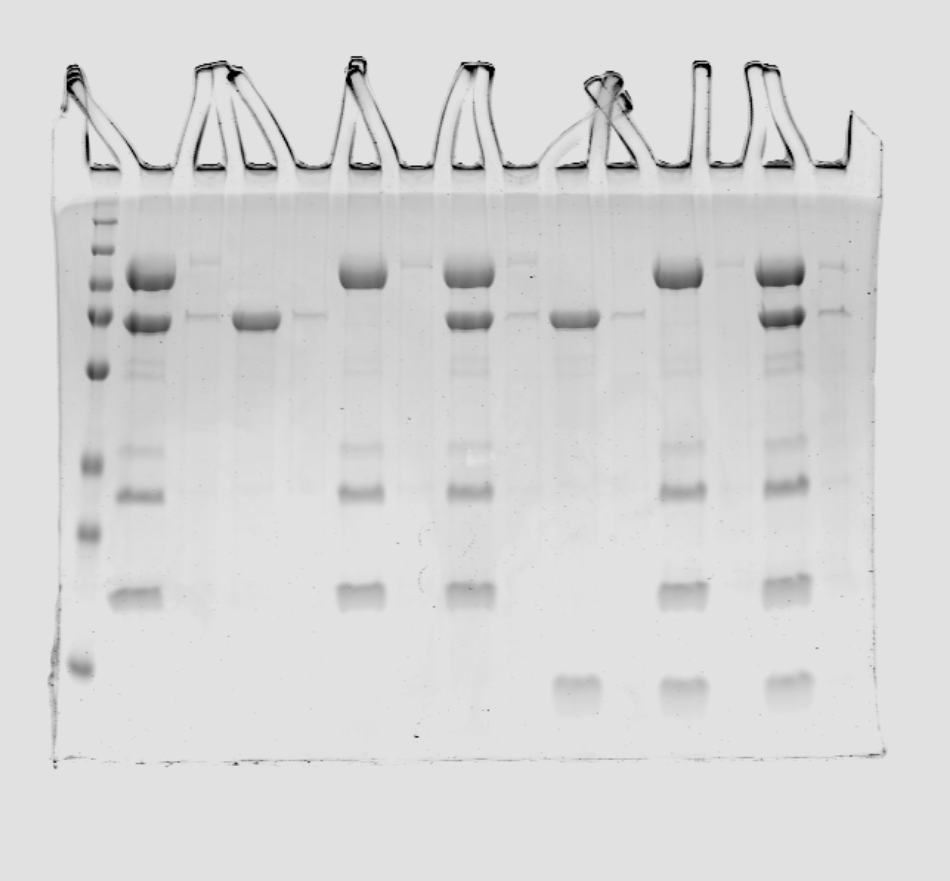

Supplement: Supplementary file 8 — EV and Appendix Figures Source Data [file 44319_2024_340_MOESM8_ESM.zip › EMBOR-2024-59048V3_SourceDataForExpandedView+Appendix/Figure EV4/EV4D/EV4D Replicate 2.png]

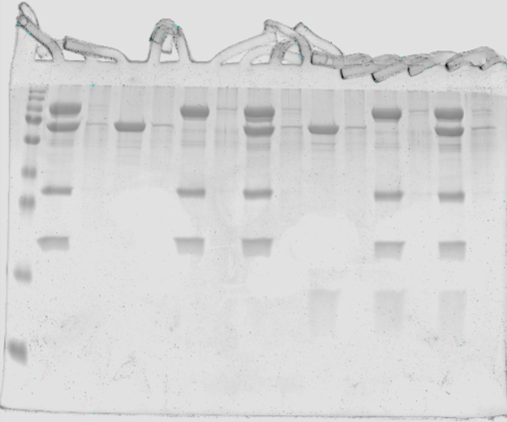

Supplement: Supplementary file 8 — EV and Appendix Figures Source Data [file 44319_2024_340_MOESM8_ESM.zip › EMBOR-2024-59048V3_SourceDataForExpandedView+Appendix/Figure EV4/EV4C/EV4C Replicate 2.png]

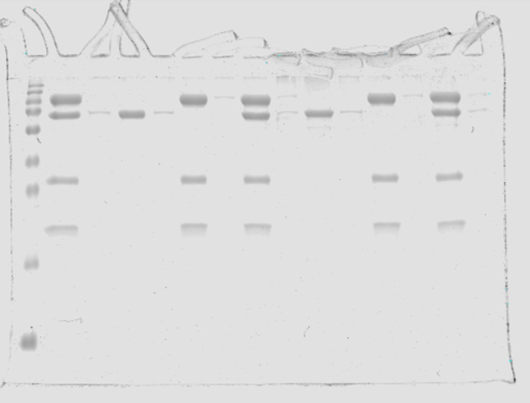

Supplement: Supplementary file 8 — EV and Appendix Figures Source Data [file 44319_2024_340_MOESM8_ESM.zip › EMBOR-2024-59048V3_SourceDataForExpandedView+Appendix/Figure EV4/EV4C/EV4C Replicate 1.png]

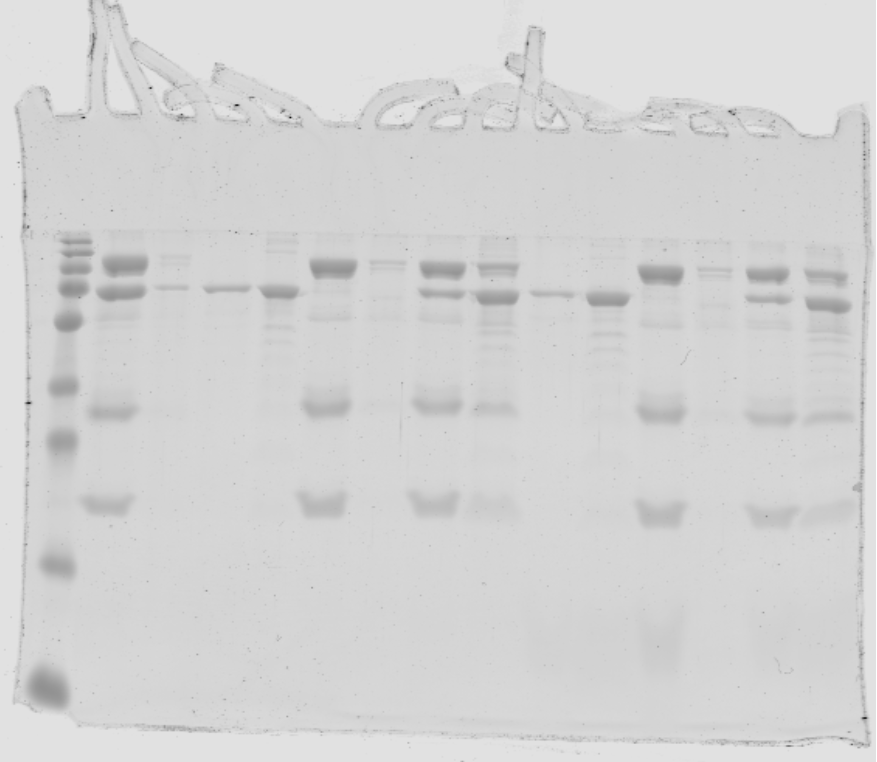

Supplement: Supplementary file 8 — EV and Appendix Figures Source Data [file 44319_2024_340_MOESM8_ESM.zip › EMBOR-2024-59048V3_SourceDataForExpandedView+Appendix/Figure EV4/EV4B/EV4B Replicate 2.png]

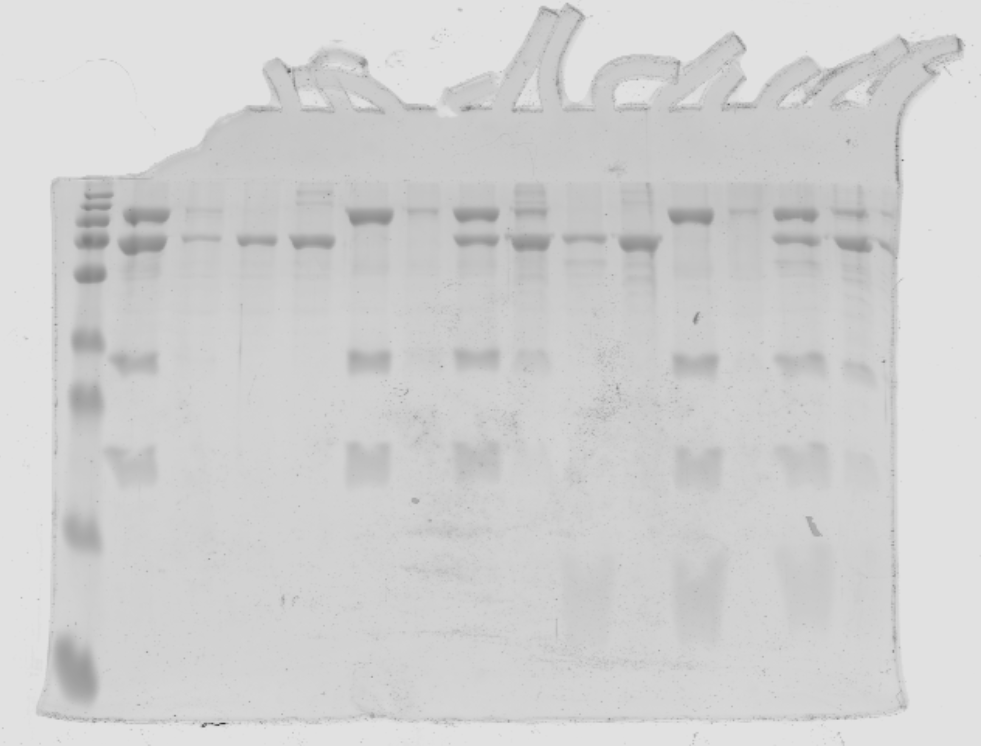

Supplement: Supplementary file 8 — EV and Appendix Figures Source Data [file 44319_2024_340_MOESM8_ESM.zip › EMBOR-2024-59048V3_SourceDataForExpandedView+Appendix/Figure EV4/EV4B/EV4B Replicate 3.png]

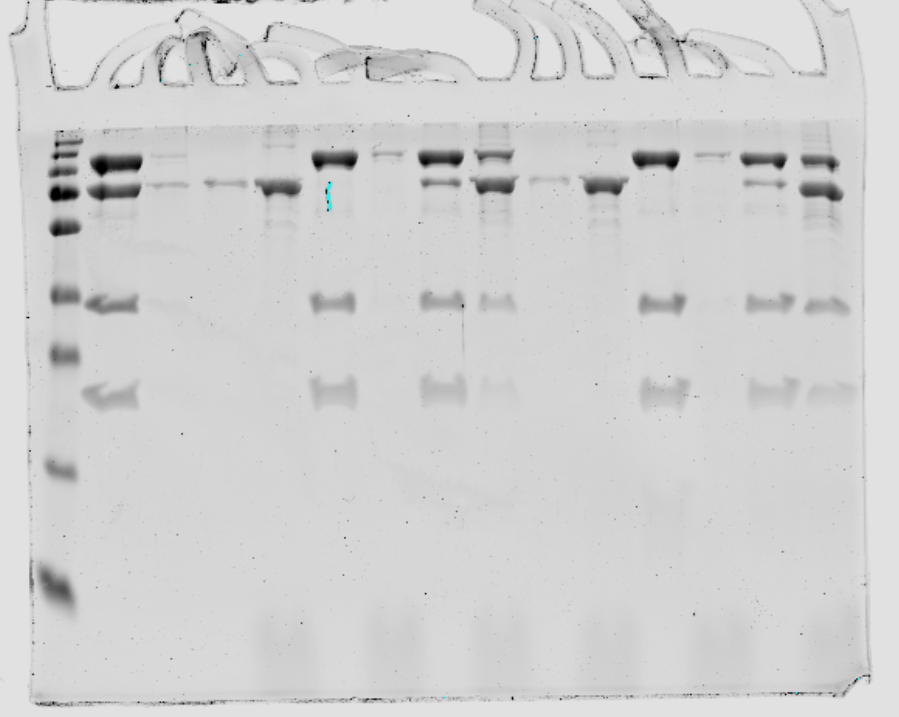

Supplement: Supplementary file 8 — EV and Appendix Figures Source Data [file 44319_2024_340_MOESM8_ESM.zip › EMBOR-2024-59048V3_SourceDataForExpandedView+Appendix/Figure EV4/EV4B/EV4B Replicate 1.png]

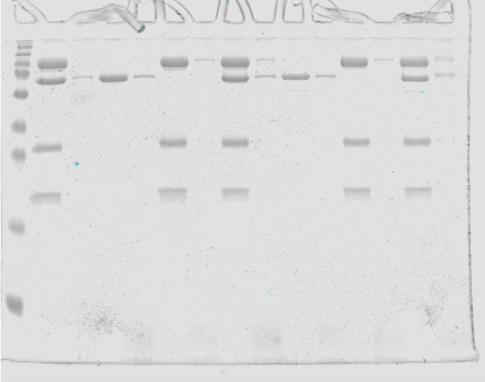

Supplement: Supplementary file 8 — EV and Appendix Figures Source Data [file 44319_2024_340_MOESM8_ESM.zip › EMBOR-2024-59048V3_SourceDataForExpandedView+Appendix/Figure EV4/EV4E/EV4E Replicate 1.png]

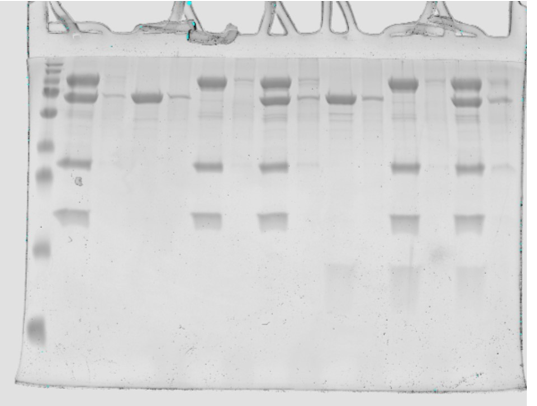

Supplement: Supplementary file 8 — EV and Appendix Figures Source Data [file 44319_2024_340_MOESM8_ESM.zip › EMBOR-2024-59048V3_SourceDataForExpandedView+Appendix/Figure EV4/EV4E/EV4E Replicate 2.png]

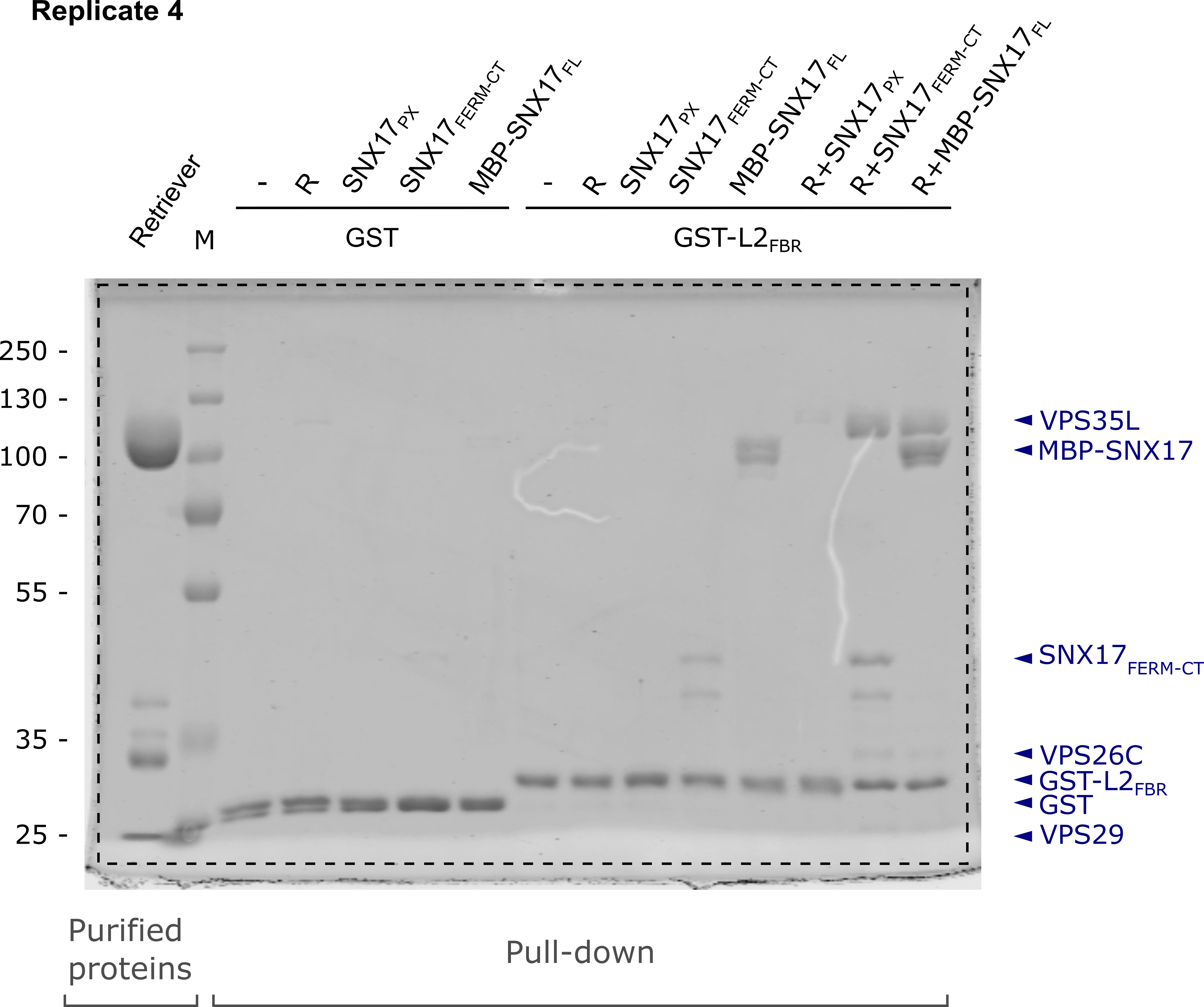

Supplement: Supplementary file 8 — EV and Appendix Figures Source Data [file 44319_2024_340_MOESM8_ESM.zip › EMBOR-2024-59048V3_SourceDataForExpandedView+Appendix/Figure EV3/EV3A/EV3A replicate 4.png]

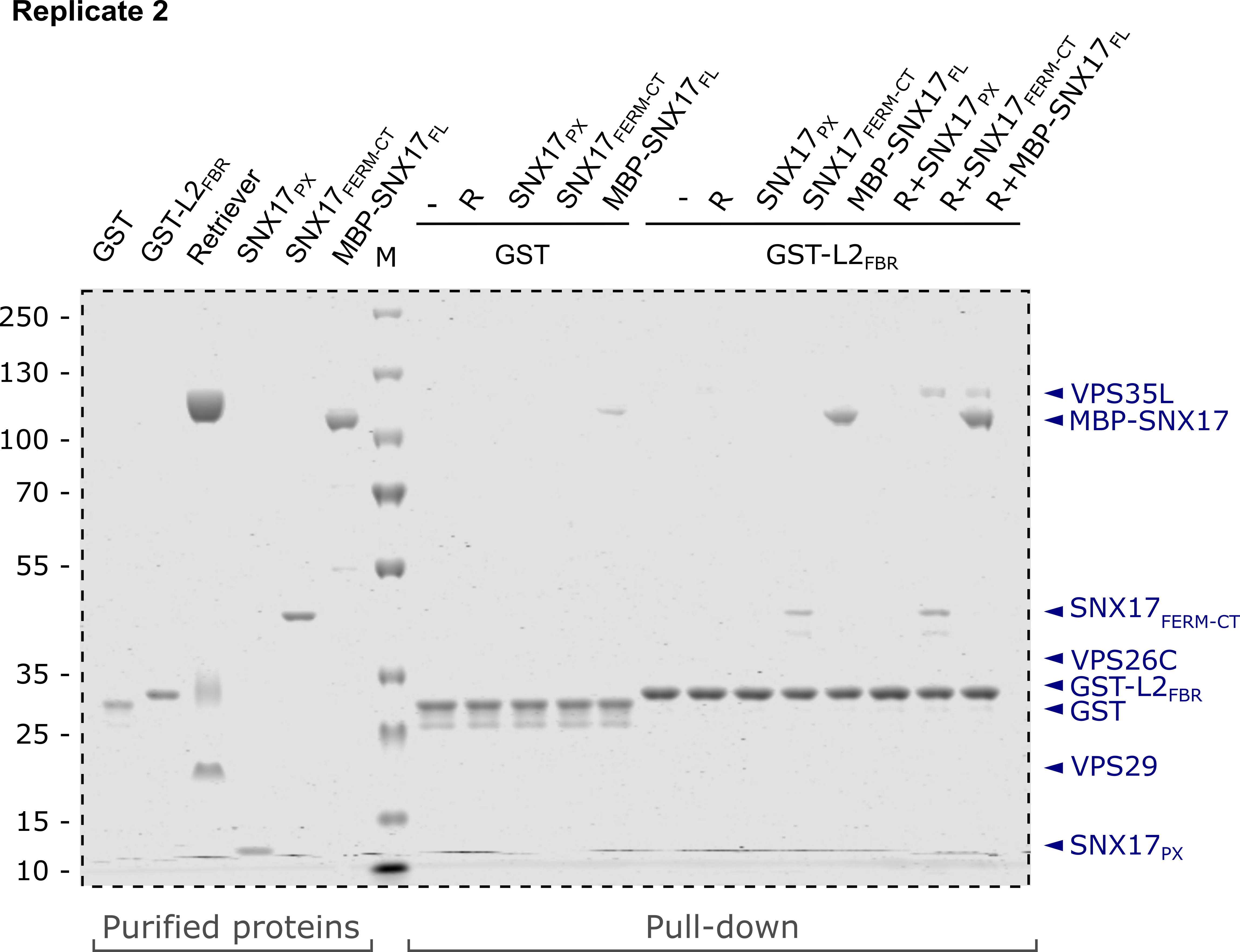

Supplement: Supplementary file 8 — EV and Appendix Figures Source Data [file 44319_2024_340_MOESM8_ESM.zip › EMBOR-2024-59048V3_SourceDataForExpandedView+Appendix/Figure EV3/EV3A/EV3A replicate 2.png]

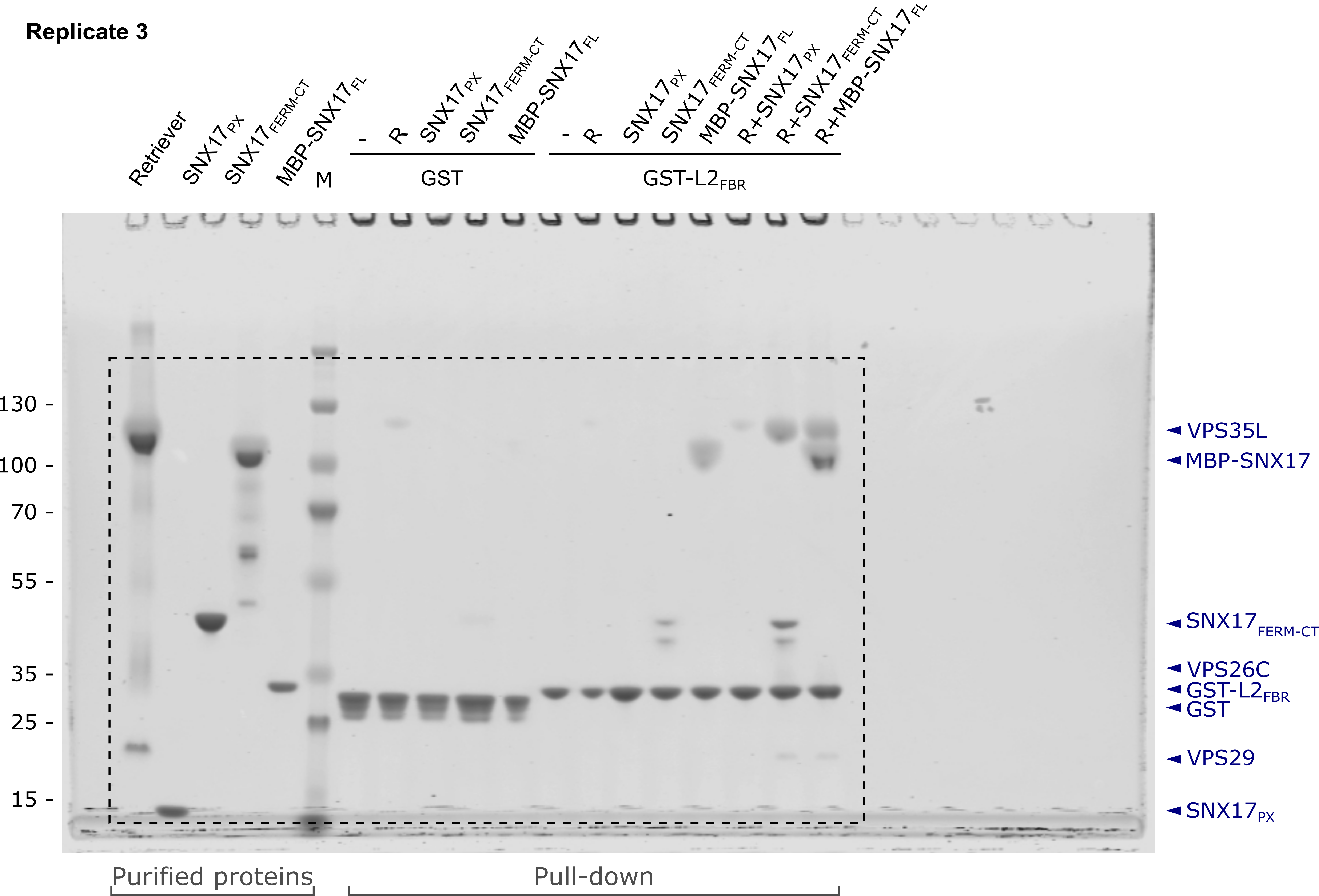

Supplement: Supplementary file 8 — EV and Appendix Figures Source Data [file 44319_2024_340_MOESM8_ESM.zip › EMBOR-2024-59048V3_SourceDataForExpandedView+Appendix/Figure EV3/EV3A/EV3A replicate 3.png]

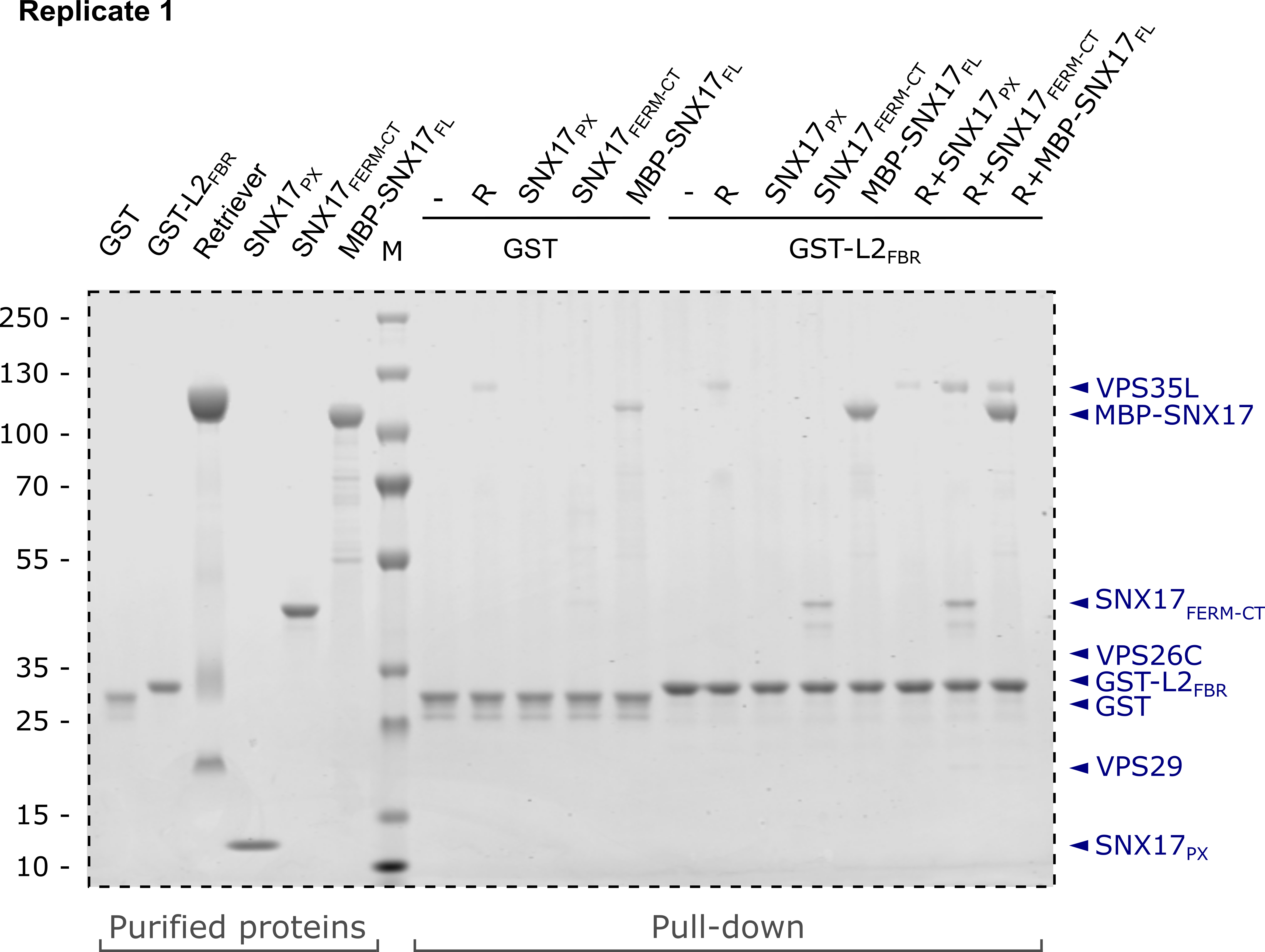

Supplement: Supplementary file 8 — EV and Appendix Figures Source Data [file 44319_2024_340_MOESM8_ESM.zip › EMBOR-2024-59048V3_SourceDataForExpandedView+Appendix/Figure EV3/EV3A/EV3A replicate 1.png]

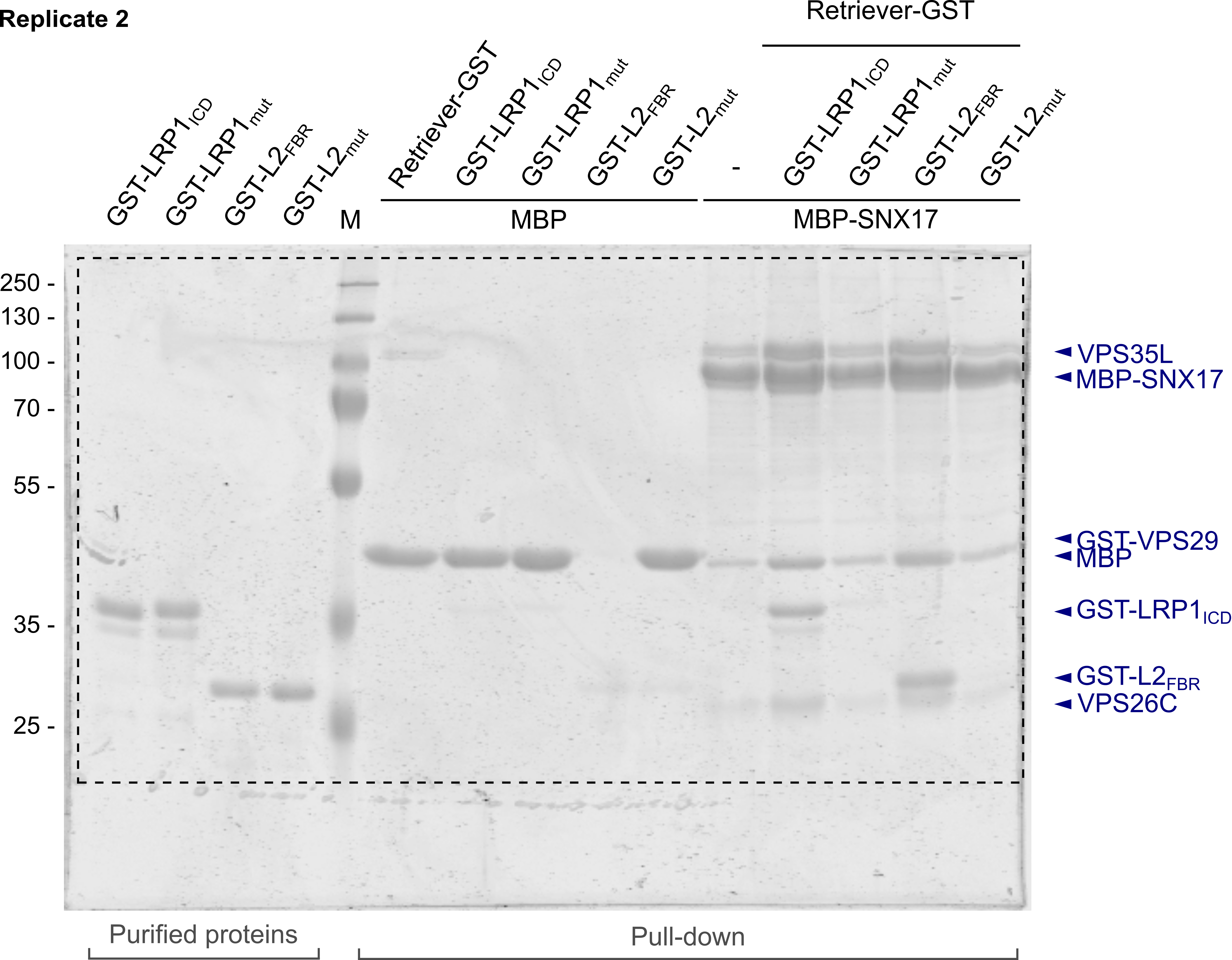

Supplement: Supplementary file 8 — EV and Appendix Figures Source Data [file 44319_2024_340_MOESM8_ESM.zip › EMBOR-2024-59048V3_SourceDataForExpandedView+Appendix/Figure EV2/EV2B/EV2B replicate 2.png]

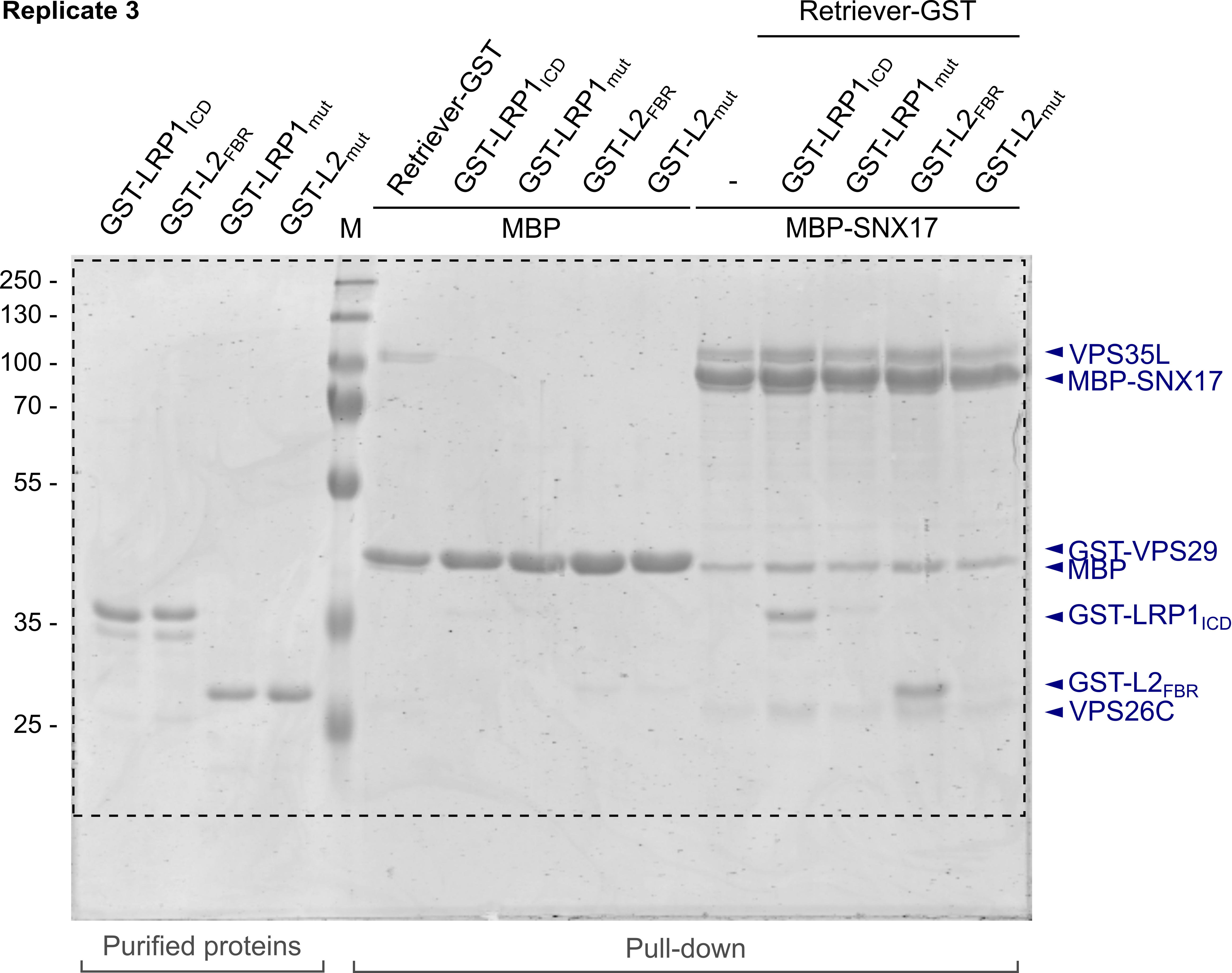

Supplement: Supplementary file 8 — EV and Appendix Figures Source Data [file 44319_2024_340_MOESM8_ESM.zip › EMBOR-2024-59048V3_SourceDataForExpandedView+Appendix/Figure EV2/EV2B/EV2B replicate 3.png]

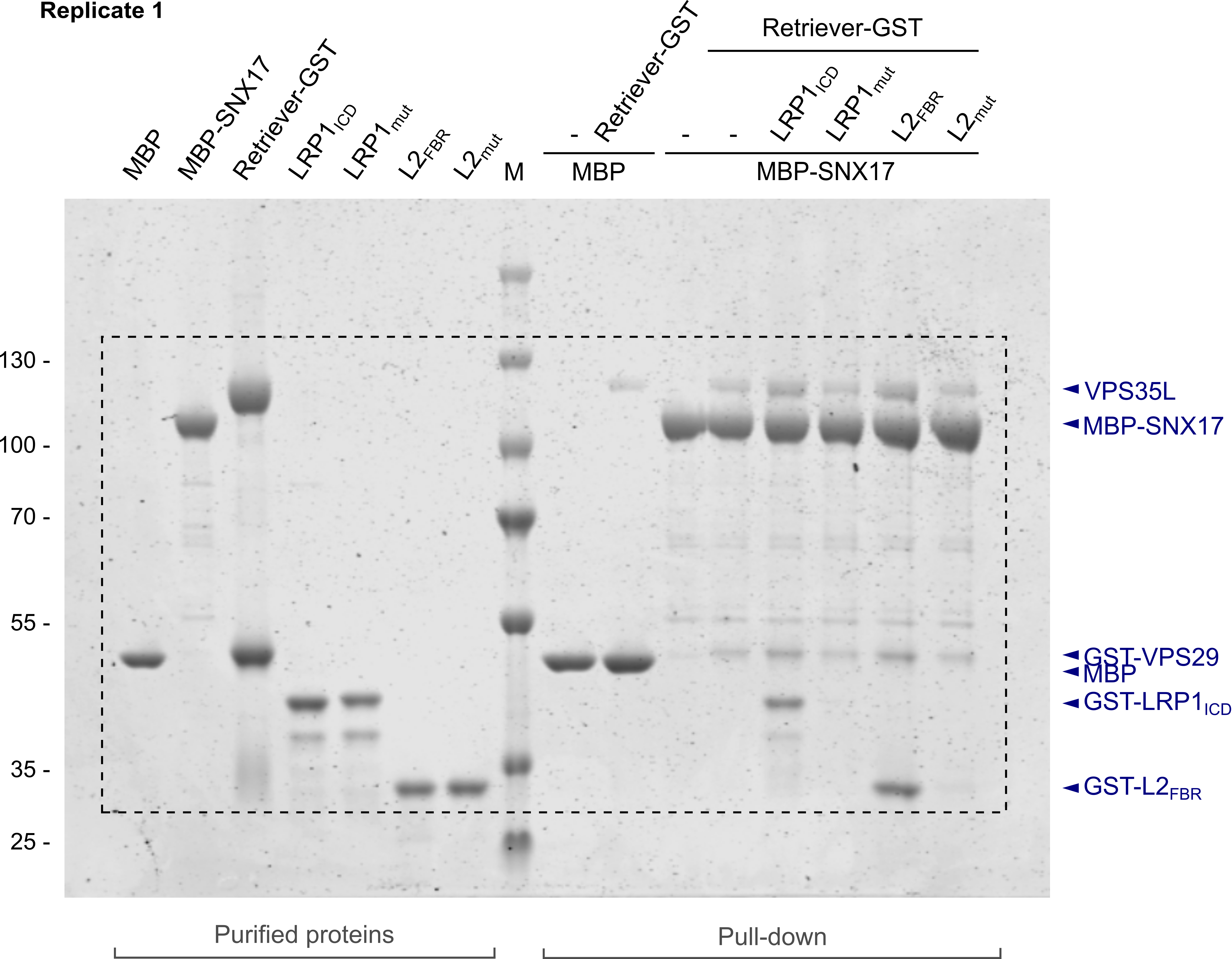

Supplement: Supplementary file 8 — EV and Appendix Figures Source Data [file 44319_2024_340_MOESM8_ESM.zip › EMBOR-2024-59048V3_SourceDataForExpandedView+Appendix/Figure EV2/EV2B/EV2B replicate 1.png]

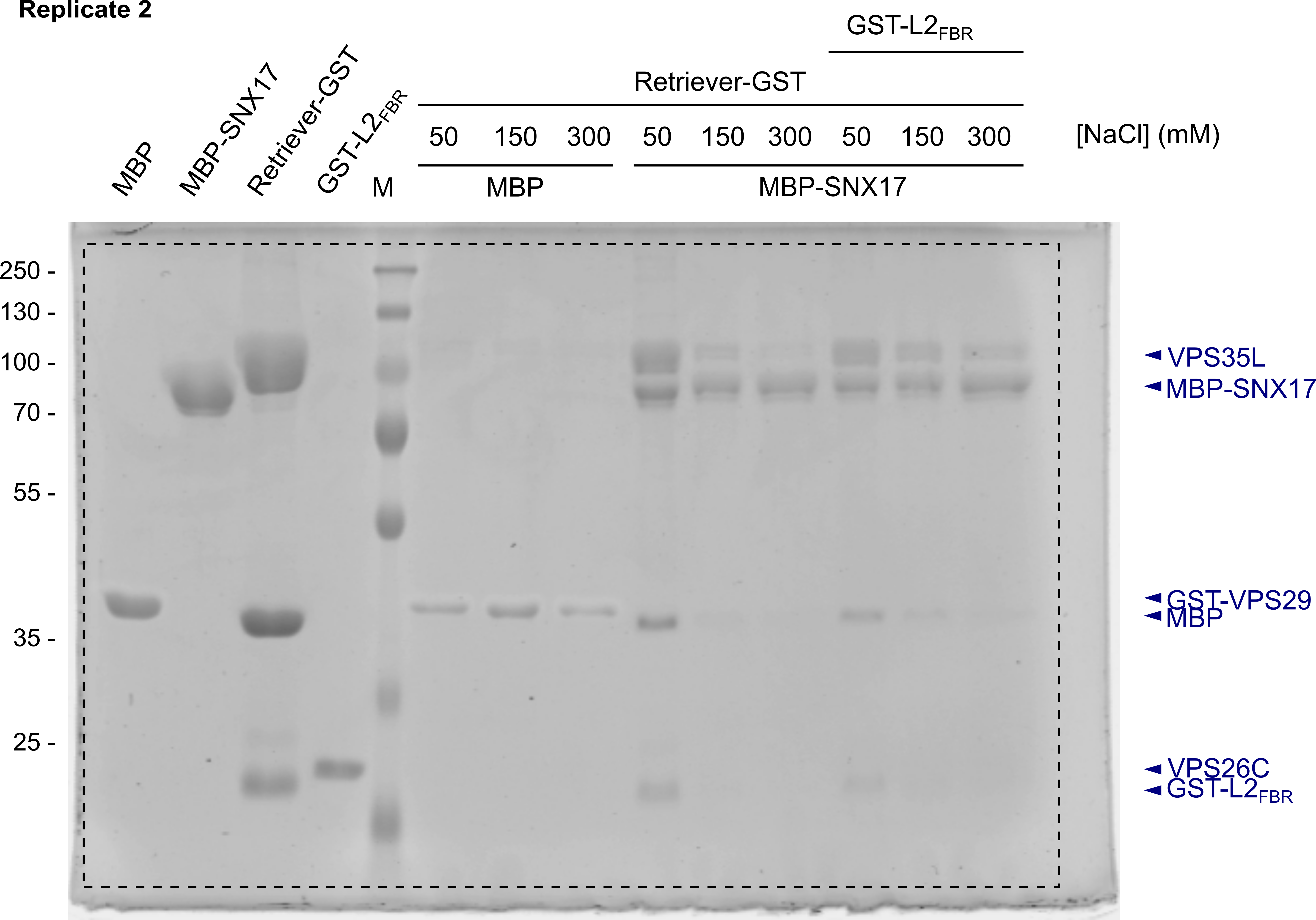

Supplement: Supplementary file 8 — EV and Appendix Figures Source Data [file 44319_2024_340_MOESM8_ESM.zip › EMBOR-2024-59048V3_SourceDataForExpandedView+Appendix/Figure EV2/EV2C/EV2C replicate 2.png]

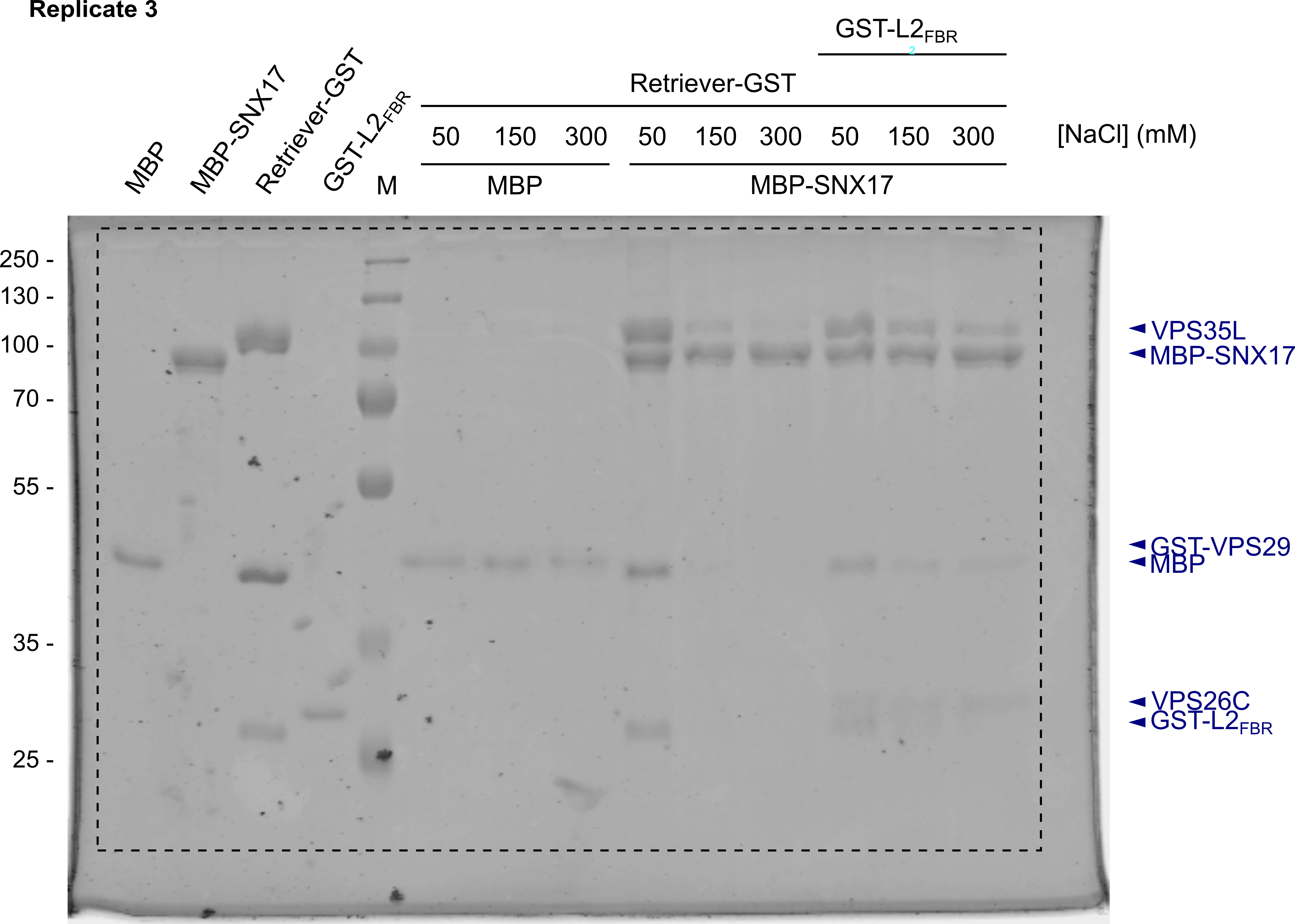

Supplement: Supplementary file 8 — EV and Appendix Figures Source Data [file 44319_2024_340_MOESM8_ESM.zip › EMBOR-2024-59048V3_SourceDataForExpandedView+Appendix/Figure EV2/EV2C/EV2C replicate 3.png]

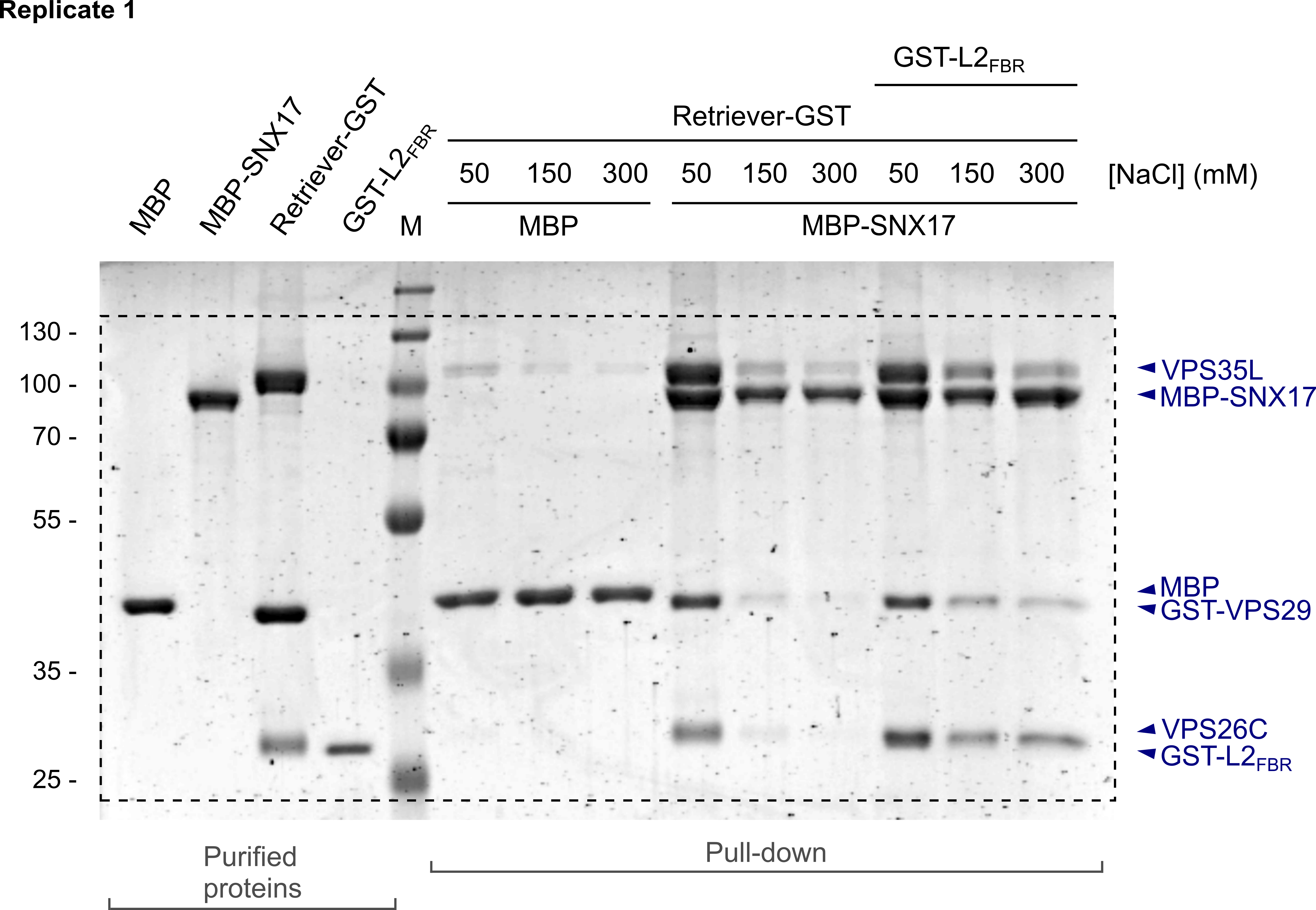

Supplement: Supplementary file 8 — EV and Appendix Figures Source Data [file 44319_2024_340_MOESM8_ESM.zip › EMBOR-2024-59048V3_SourceDataForExpandedView+Appendix/Figure EV2/EV2C/EV2C replicate 1.png]

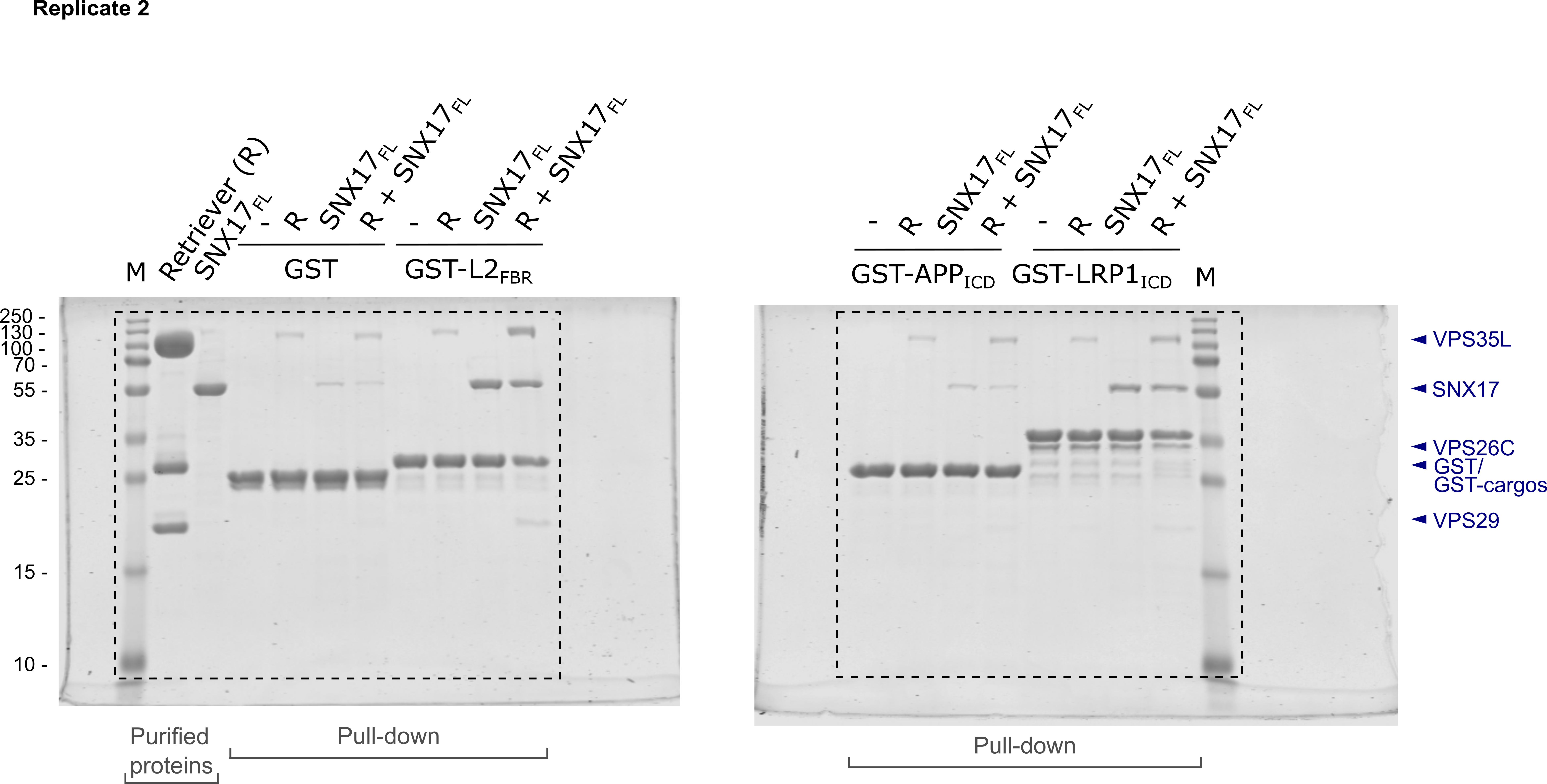

Supplement: Supplementary file 8 — EV and Appendix Figures Source Data [file 44319_2024_340_MOESM8_ESM.zip › EMBOR-2024-59048V3_SourceDataForExpandedView+Appendix/Figure EV2/EV2A/EV2A replicate 2.png]

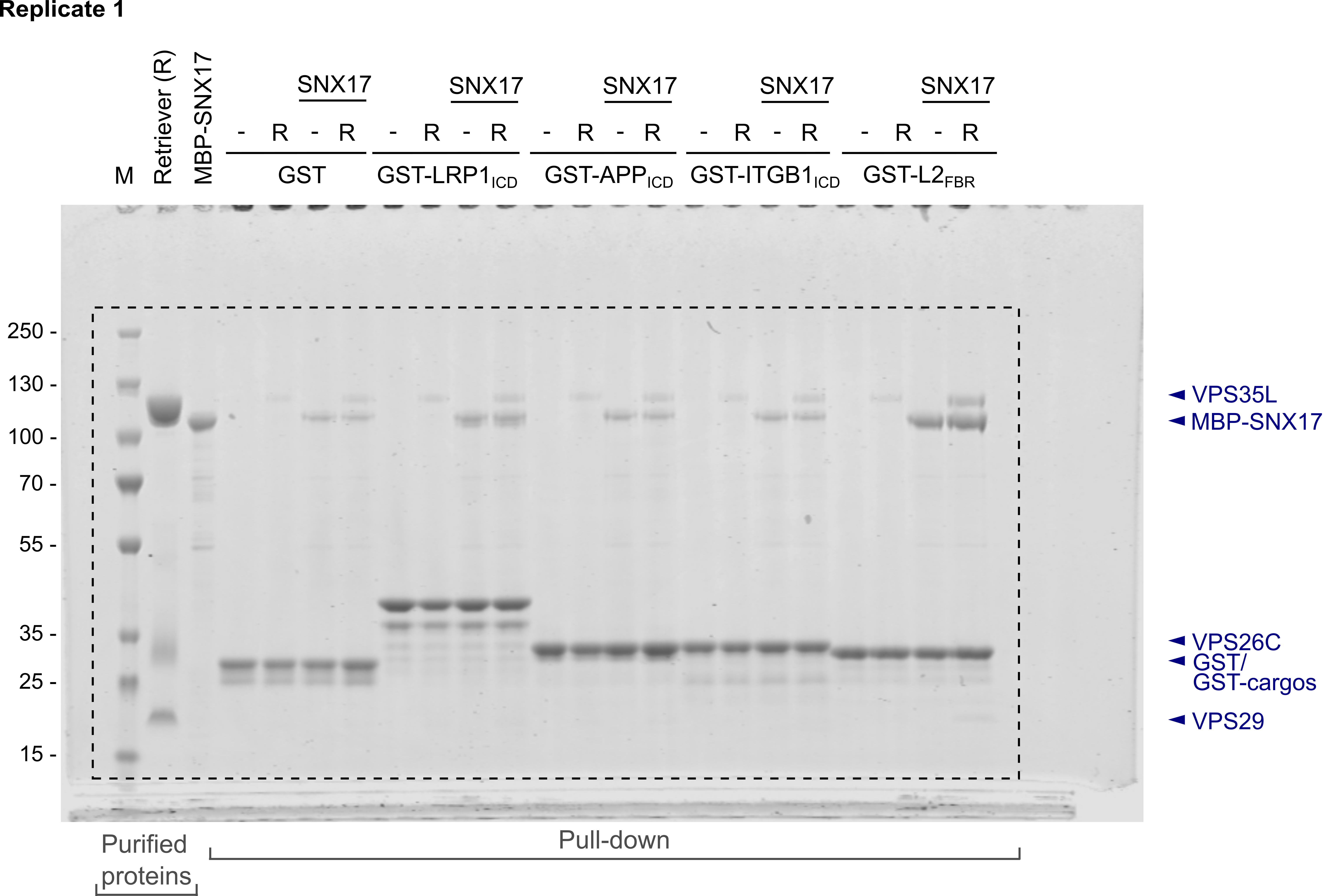

Supplement: Supplementary file 8 — EV and Appendix Figures Source Data [file 44319_2024_340_MOESM8_ESM.zip › EMBOR-2024-59048V3_SourceDataForExpandedView+Appendix/Figure EV2/EV2A/EV2A replicate 1.png]

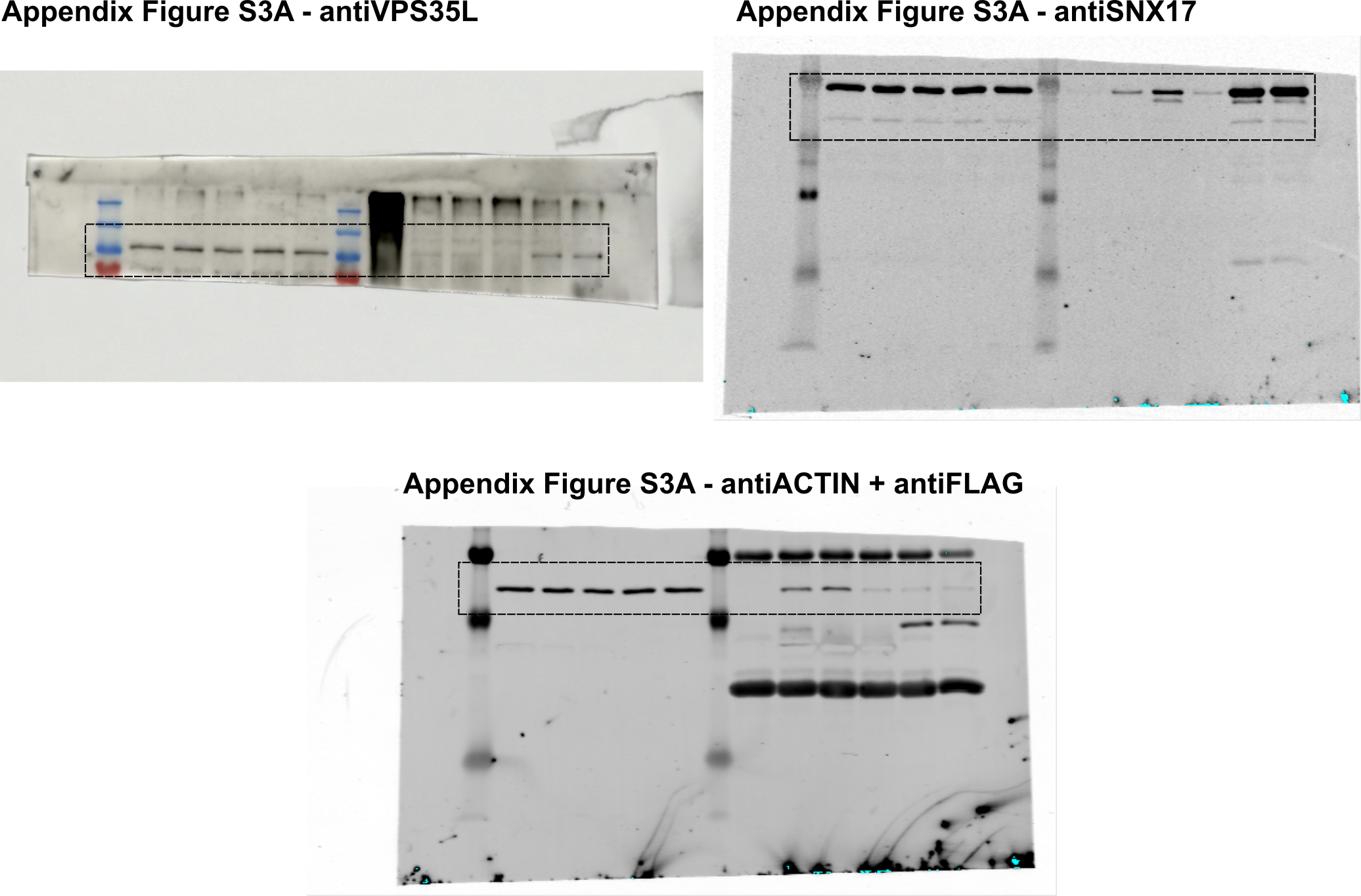

Supplement: Supplementary file 8 — EV and Appendix Figures Source Data [file 44319_2024_340_MOESM8_ESM.zip › EMBOR-2024-59048V3_SourceDataForExpandedView+Appendix/Figure S3/S3A/S3A.png]

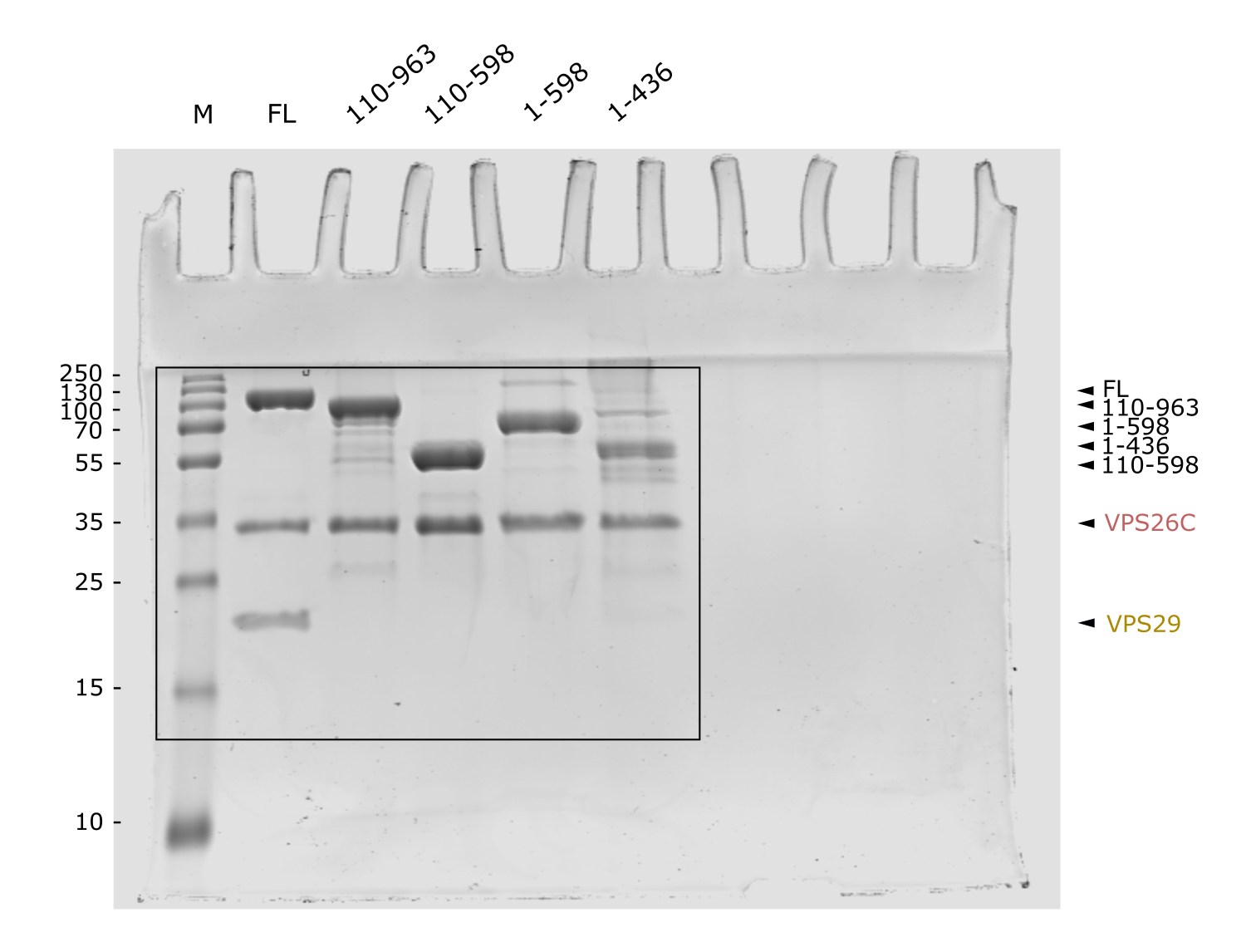

Supplement: Supplementary file 8 — EV and Appendix Figures Source Data [file 44319_2024_340_MOESM8_ESM.zip › EMBOR-2024-59048V3_SourceDataForExpandedView+Appendix/Figure EV1/EV1B/EV1B_gel.png]

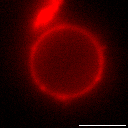

Supplement: Supplementary file 8 — EV and Appendix Figures Source Data [file 44319_2024_340_MOESM8_ESM.zip › EMBOR-2024-59048V3_SourceDataForExpandedView+Appendix/Figure EV4/EV4A/GUVS + Retriever-mKate + MBP-SNX17_WT/GUVS+Retriever-mKate+MBP-SNX17_WT red.png]

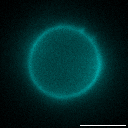

Supplement: Supplementary file 8 — EV and Appendix Figures Source Data [file 44319_2024_340_MOESM8_ESM.zip › EMBOR-2024-59048V3_SourceDataForExpandedView+Appendix/Figure EV4/EV4A/GUVS + Retriever-mKate + MBP-SNX17_WT/GUVS+Retriever-mKate+MBP-SNX17_WT cyan.png]

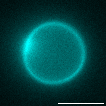

Supplement: Supplementary file 8 — EV and Appendix Figures Source Data [file 44319_2024_340_MOESM8_ESM.zip › EMBOR-2024-59048V3_SourceDataForExpandedView+Appendix/Figure EV4/EV4A/GUVS + Retriever-mKate + MBP-SNX17_L470G/GUVS+Retriever-mKate+MBP-SNX17_L470G cyan.png]

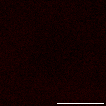

Supplement: Supplementary file 8 — EV and Appendix Figures Source Data [file 44319_2024_340_MOESM8_ESM.zip › EMBOR-2024-59048V3_SourceDataForExpandedView+Appendix/Figure EV4/EV4A/GUVS + Retriever-mKate + MBP-SNX17_L470G/GUVS+Retriever-mKate+MBP-SNX17_L470G red.png]

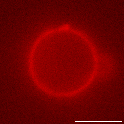

Supplement: Supplementary file 8 — EV and Appendix Figures Source Data [file 44319_2024_340_MOESM8_ESM.zip › EMBOR-2024-59048V3_SourceDataForExpandedView+Appendix/Figure EV4/EV4A/GUVS + Retriever-mKate + MBP-SNX17_W321A/GUVS+Retriever-mKate+MBP-SNX17_W321A red.png]

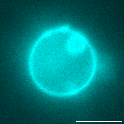

Supplement: Supplementary file 8 — EV and Appendix Figures Source Data [file 44319_2024_340_MOESM8_ESM.zip › EMBOR-2024-59048V3_SourceDataForExpandedView+Appendix/Figure EV4/EV4A/GUVS + Retriever-mKate + MBP-SNX17_W321A/GUVS+Retriever-mKate+MBP-SNX17_W321A cyan.png]

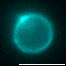

Supplement: Supplementary file 8 — EV and Appendix Figures Source Data [file 44319_2024_340_MOESM8_ESM.zip › EMBOR-2024-59048V3_SourceDataForExpandedView+Appendix/Figure EV4/EV4A/GUVS + Retriever-mKate + MBP-SNX17_N459A+F462A/GUVS+Retriever-mKate+MBP-SNX17_N459A+F462A cyan.png]

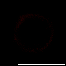

Supplement: Supplementary file 8 — EV and Appendix Figures Source Data [file 44319_2024_340_MOESM8_ESM.zip › EMBOR-2024-59048V3_SourceDataForExpandedView+Appendix/Figure EV4/EV4A/GUVS + Retriever-mKate + MBP-SNX17_N459A+F462A/GUVS+Retriever-mKate+MBP-SNX17_N459A+F462A red.png]

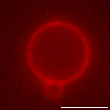

Supplement: Supplementary file 8 — EV and Appendix Figures Source Data [file 44319_2024_340_MOESM8_ESM.zip › EMBOR-2024-59048V3_SourceDataForExpandedView+Appendix/Figure EV4/EV4A/GUVS + Retriever-mKate + MBP-SNX17_V380D/GUVS+Retriever-mKate+MBP-SNX17_V380D red.png]

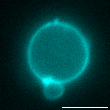

Supplement: Supplementary file 8 — EV and Appendix Figures Source Data [file 44319_2024_340_MOESM8_ESM.zip › EMBOR-2024-59048V3_SourceDataForExpandedView+Appendix/Figure EV4/EV4A/GUVS + Retriever-mKate + MBP-SNX17_V380D/GUVS+Retriever-mKate+MBP-SNX17_V380D cyan.png]

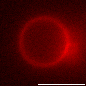

Supplement: Supplementary file 8 — EV and Appendix Figures Source Data [file 44319_2024_340_MOESM8_ESM.zip › EMBOR-2024-59048V3_SourceDataForExpandedView+Appendix/Figure EV4/EV4A/GUVS + Retriever-mKate + MBP-SNX17_H457A/GUVS+Retriever-mKate+MBP-SNX17_H457A red.png]

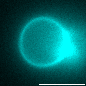

Supplement: Supplementary file 8 — EV and Appendix Figures Source Data [file 44319_2024_340_MOESM8_ESM.zip › EMBOR-2024-59048V3_SourceDataForExpandedView+Appendix/Figure EV4/EV4A/GUVS + Retriever-mKate + MBP-SNX17_H457A/GUVS+Retriever-mKate+MBP-SNX17_H457A cyan.png]

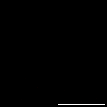

Supplement: Supplementary file 8 — EV and Appendix Figures Source Data [file 44319_2024_340_MOESM8_ESM.zip › EMBOR-2024-59048V3_SourceDataForExpandedView+Appendix/Figure EV4/EV4A/GUVS + Retriever-mKate + MBP-SNX17_D467X/GUVS+Retriever-mKate+MBP-SNX17_D467X red.png]

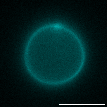

Supplement: Supplementary file 8 — EV and Appendix Figures Source Data [file 44319_2024_340_MOESM8_ESM.zip › EMBOR-2024-59048V3_SourceDataForExpandedView+Appendix/Figure EV4/EV4A/GUVS + Retriever-mKate + MBP-SNX17_D467X/GUVS+Retriever-mKate+MBP-SNX17_D467X cyan.png]
