# Supplementary material for: Selective cargo and membrane recognition by SNX17 regulates its interaction with Retriever
Source: EMBO Rep. 2024 Dec 9;26(2):470–93. doi: 10.1038/s44319-024-00340-1 (PMC11772769; doi:10.1038/s44319-024-00340-1)
Supplement: Supplementary file 9 — Expanded View Figures [file 44319_2024_340_MOESM9_ESM.pdf]

Expanded View Figures

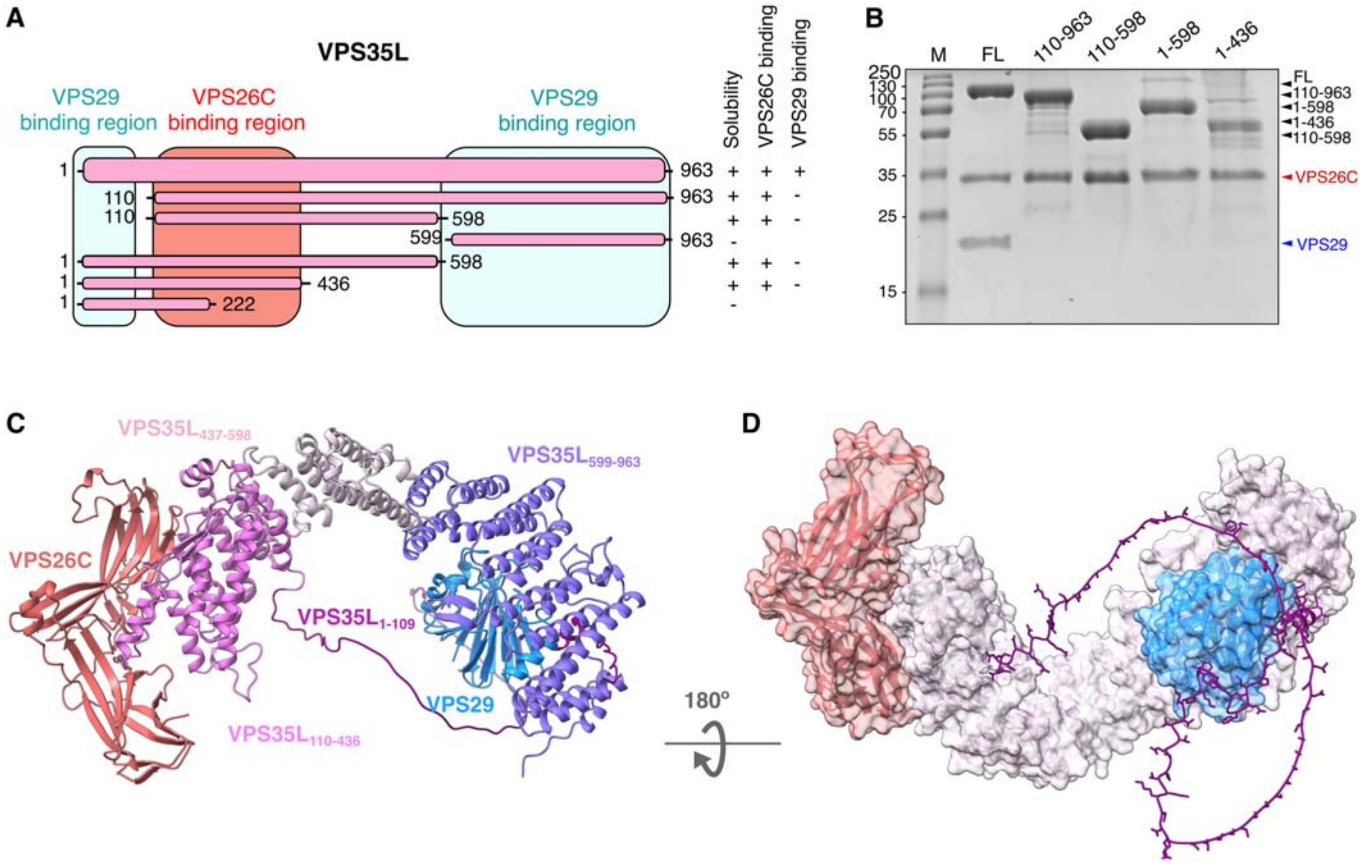

Figure EV1. Purification and stability assessment of the Retriever complex.

(**A**) Summary of the following features of purified Retriever complexes containing VPS26C, VPS29, and different length constructs of VPS35L: solubility, VPS26C binding, and VPS29 binding. The experimentally observed interaction region of VPS35L with VPS26C is highlighted with a red box and with VPS29 with two cyan boxes.
(**B**) Coomassie-stained SDS-PAGE gel of purified Retriever constructs with different VPS35L truncations. (**C**) AF2 model of the Retriever complex with the experimentally observed regions of interaction highlighted. The interaction region of VPS35L with VPS26C ($VPS35L_{110-436}$) is in pink, the C-terminal interaction region of VPS35L with VPS29 ($VPS35L_{599-963}$) is in violet, and the N-terminal ($VPS35L_{1-109}$) is in dark purple. Model Archive ID: ma-3cag5. (**D**) Detail of the intramolecular interaction of the amino and carboxy-terminal regions of VPS35L. VPS26C, VPS29, and $VPS35L_{111-963}$ are represented by a ribbon diagram with a transparent surface. $VPS35L_{1-110}$ is displayed in sticks. Source data are available online for this figure.

 © The Author(s)

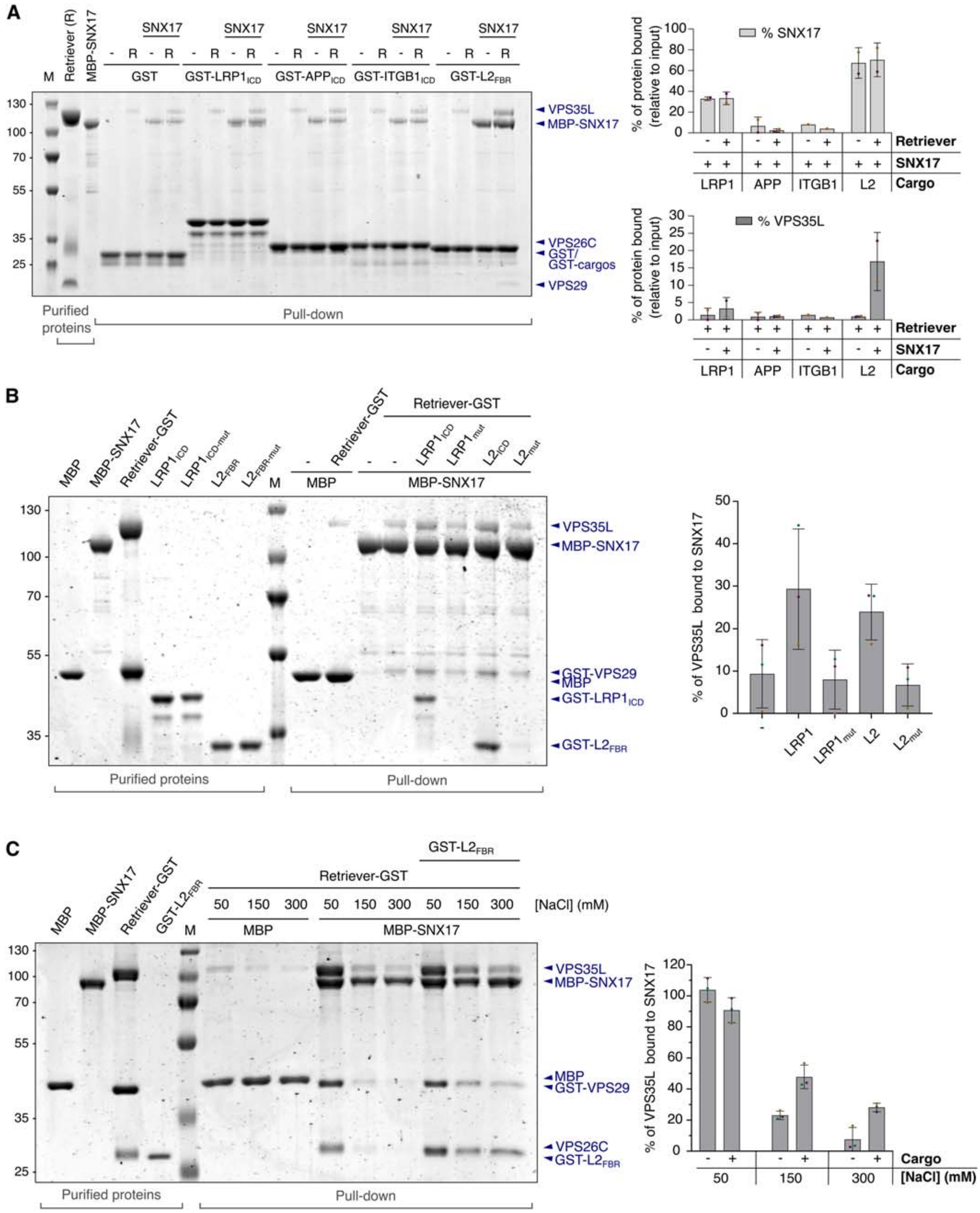
A
M
Retriever (R)
MBP-SNX17
SNX17
- R - R
GST
GST-LRP1ICD
GST-APPICD
GST-ITGB1ICD
GST-L2FBR
130
100
70
55
35
25
VPS35L
MBP-SNX17
VPS26C
GST/
GST-cargos
VPS29
Purified proteins
Pull-down
% of protein bound (relative to input)
% SNX17
Retriever
SNX17
Cargo
LRP1
APP
ITGB1
L2
% VPS35L
B
MBP
MBP-SNX17
Retriever-GST
LRP1ICD
LRP1ICD-mut
L2FBR
L2FBR-mut
M
Retriever-GST
LRP1ICD
LRP1mut
L2ICD
L2mut
MBP
MBP-SNX17
VPS35L
MBP-SNX17
GST-VPS29
MBP
GST-LRP1ICD
GST-L2FBR
Purified proteins
Pull-down
% of VPS35L bound to SNX17
LRP1
LRP1mut
L2
L2mut
C
MBP
MBP-SNX17
Retriever-GST
GST-L2FBR
M
Retriever-GST
GST-L2FBR
50 150 300 50 150 300 50 150 300
[NaCl] (mM)
MBP
MBP-SNX17
VPS35L
MBP-SNX17
MBP
GST-VPS29
VPS26C
GST-L2FBR
Purified proteins
Pull-down
% of VPS35L bound to SNX17
Cargo
[NaCl] (mM)

Figure EV2. Cargo-dependent interaction of SNX17 with Retriever.

(**A**) The Retriever complex was incubated with MBP-SNX17 in the presence of GST-LRP1$_{ICD}$, GST-APP$_{ICD}$, GST-ITGB1$_{ICD}$, and GST-L2$_{FBR}$ in GST pull-down assays. Non-fused GST protein was used as a negative control. Purified proteins and pull-down samples were separated by SDS-PAGE and visualized by Coomassie Blue staining (a representative gel shown). The right panel presents the densitometry-based quantification of the amount of SNX17 or Retriever retained in the cargo-GST pull-down assays. VPS35L was used as a representative band of the Retriever complex. The band intensities of SNX17 and VPS35L were normalized to the GST or GST-cargo band intensity. Non-specific binding to GST was subtracted. The percentage of SNX17 or VPS35L binding to GST-cargos was calculated as the ratio of the pull-down protein to the input protein (lanes 2 and 3). Values represent the mean ± SD of two independent experiments. (**B**) The effect of mutating the conserved NPxY motif to APxA in LRP1 and L2 on the cargo-dependent Retriever-SNX17 interaction. Coomassie-stained SDS-PAGE gel of pull-down assays with MBP-SNX17 and Retriever in the presence of GST-LRP1$_{ICD}$, GST-LRP1$_{ICD\text{-}mut}$ (N4470A + Y4473A), GST-L2$_{FBR}$, and GST-L2$_{FBR\text{-}mut}$ (N254A + Y257A). Retriever binding to MBP-SNX17 was quantified as described in Fig. 2D. Values represent the mean ± SD of three independent experiments. (**C**) MBP pull-down assays to examine the impact of salt concentration on the SNX17-Retriever interaction in the presence or absence of cargo. The Coomassie-stained SDS-PAGE gel shown is a representative image of three independent experiments. MBP was included as a control for nonspecific binding. Retriever binding to MBP-SNX17 was quantified as described in Fig. 2D. Error bars represent the standard deviation of three technical replicates. M protein marker, R Retriever. Source data are available online for this figure.

 © The Author(s)

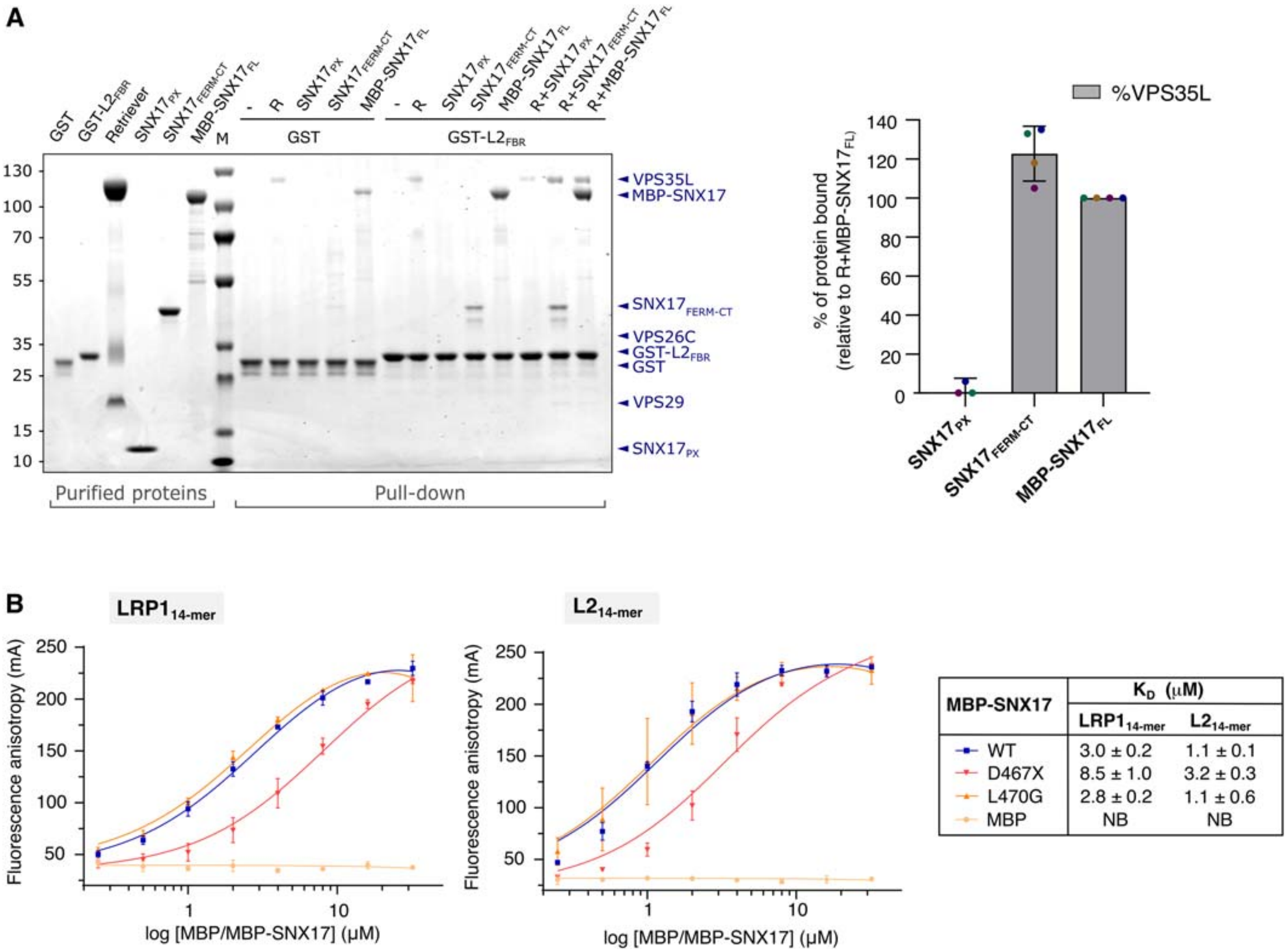

Figure EV3. Mapping the interaction between SNX17 and the Retriever complex.

(**A**) GST pull-down assays to map the region of SNX17 that interacts with Retriever. GST-L2$_{FBR}$ was incubated with the indicated combinations of SNX17$_{PX}$, SNX17$_{FERM\text{-}CT}$ and MBP-SNX17$_{FL}$. Non-fused GST protein was used as a negative control. Samples were loaded onto an SDS-PAGE gel and stained with Coomassie Blue. Densitometry-based quantification was carried out with ImageJ, measuring VPS35L as a representative band of the Retriever complex. The band intensities of VPS35L were normalized to the GST or GST-cargo band intensity. Non-specific binding to GST was subtracted. The percentage of VPS35L bound in the presence of MBP-SNX17$_{FL}$ and GST-L2$_{FBR}$ was set to 100%, and the values for the other conditions were calculated relative to this. Values represent mean ± SD based on four technical replicates. M protein marker, R Retriever complex, FL full-length. (**B**) Effect of SNX17 mutants of the Retriever-binding region on cargo binding affinity. Fluorescence anisotropy binding curves of 5-FAM-labeled LRP1$_{14\text{-}mer}$ or L2$_{14\text{-}mer}$ peptide titrated with indicated SNX17 mutants. Data points are the mean ± SD of two biological replicates, with MBP-SNX17 and its mutants obtained from two independent protein purifications. The estimated K_D ± SD of each mutant is listed in the right panel. MBP is used as a negative control. NB no detectable binding. Source data are available online for this figure.

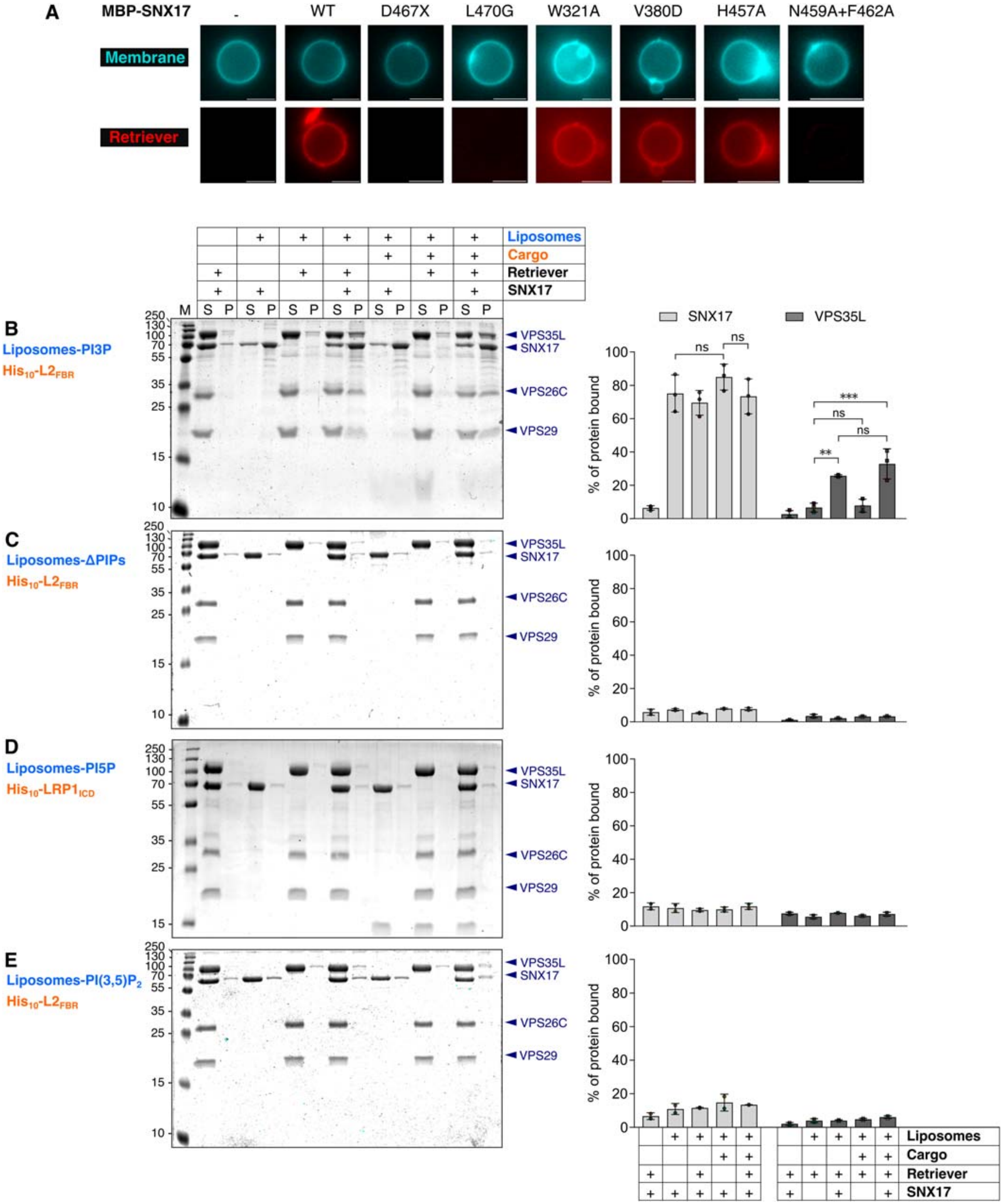

A
MBP-SNX17
-
WT
D467X
L470G
W321A
V380D
H457A
N459A+F462A
Membrane
Retriever
B
Liposomes-PI3P
His10-L2FBR
C
Liposomes-ΔPIPs
His10-L2FBR
D
Liposomes-PI5P
His10-LRP1ICD
E
Liposomes-PI(3,5)P2
His10-L2FBR
Liposomes
Cargo
Retriever
SNX17
M S P S P S P S P S P S P S P
VPS35L
SNX17
VPS26C
VPS29
SNX17
VPS35L
% of protein bound
ns
**
***

 © The Author(s)

Figure EV4. Analysis of the requirements for Retriever recruitment to membranes.

(**A**) Fluorescent imaging of GUVs stained with Marina Blue DHPE lipid dye (shown in cyan) to study the interaction of Retriever-mKate2 (red) with SNX17 WT or mutants on membranes. Scale bar: 5 µm. (**B–E**) Study of Retriever recruitment onto liposome membranes of various compositions in the presence of His-Sumo3-SNX17 and the cargo His_{10}-$L2_{FBR}$ or His_{10}-$LRP1_{ICD}$. Liposomes lacking phosphatidylinositol (**C**) or containing PI3P (**B**), PI5P (**D**), or PI(3,5)P_2 (**E**) were analyzed. Supernatant (S) and pellet (P) fractions were separated and visualized via SDS-PAGE followed by Coomassie staining (left). The binding of SNX17 and Retriever to liposomes was quantified as the percentage of total protein bound to the pellet in each condition, with VPS35L serving as a representative band of the Retriever complex (right). Bars represent mean ± SD from three (**B**) or two (**C–E**) biological replicates, derived from independent liposomes preparations and two separate protein purifications of Retriever and His-Sumo3-SNX17. One-way ANOVA followed by Tukey's test for multiple comparisons was performed for statistical analysis in (**B**). $^{**}p = 0.004$, $^{***}p = 0.0003$, ns not significant.
Source data are available online for this figure.

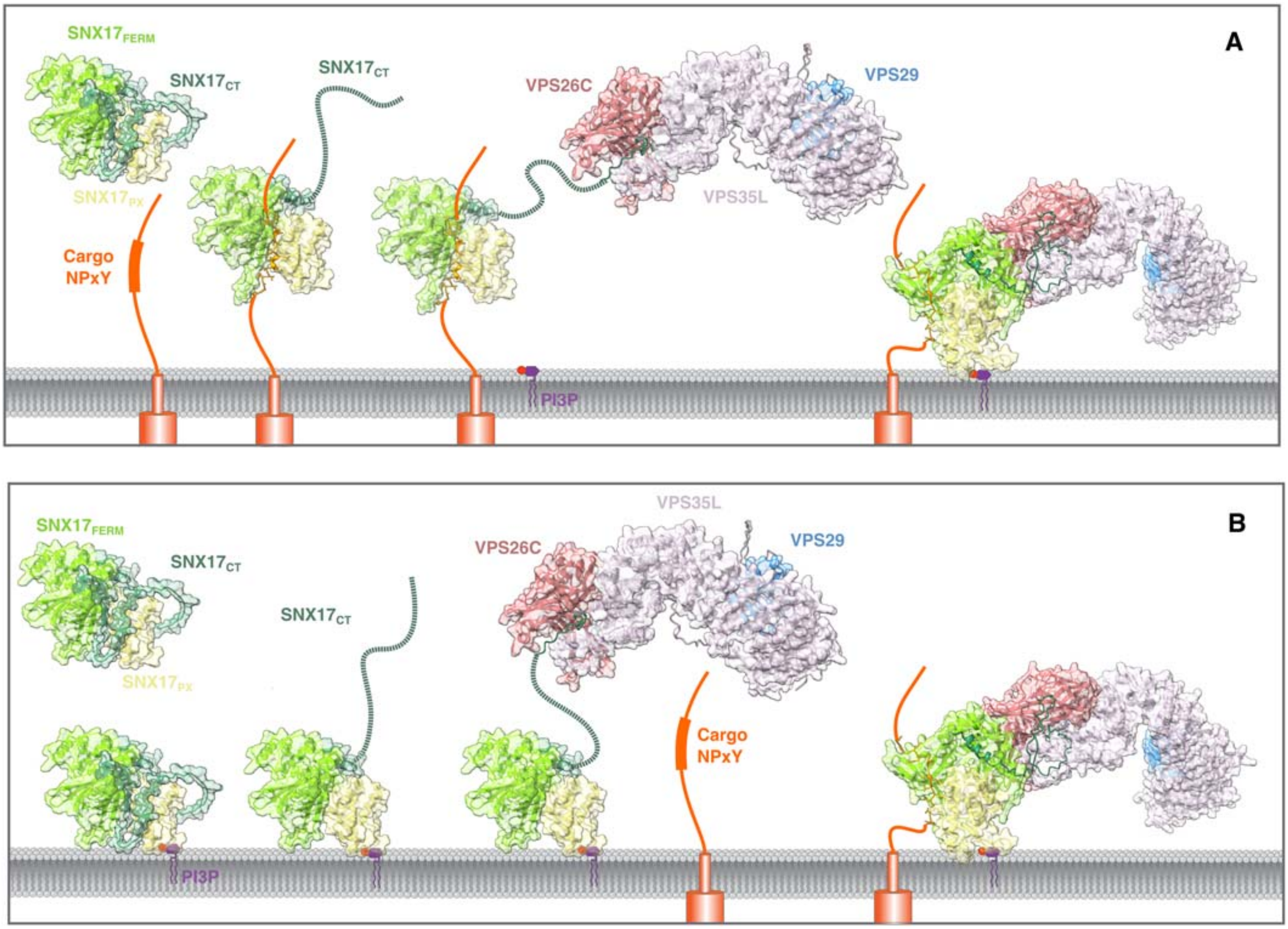

Figure EV5. Proposed activation mechanisms for the Retriever-SNX17 interaction.

(**A**) Cargo-mediated activation: SNX17 encounters its cargo, and this interaction through the FERM domain triggers the release of the SNX17 C-terminal region. With the C-terminal residues exposed, SNX17 binds and recruits Retriever. Subsequently, SNX17 binding to PI3P at the membrane through the PX domain promotes the attachment of the complex to the membrane. (**B**) Membrane-mediated activation: SNX17 initially binds to PI3P, leading to its attachment to the membrane and subsequent exposure of the Retriever-binding motif. The movement of the C-terminal residues of SNX17 enables Retriever recruitment and cargo binding. The predicted interaction between VPS26C and SNX17, observed in the AF2-multimer model for the complex SNX17:L2$_{17mer}$:VPS26C:VPS35L$_{110\text{-}598}$, was used to illustrate the proposed approach of Retriever to the membrane in (**A**, **B**).

 © The Author(s)
